# Triplet state homoaromaticity: concept, computational validation and experimental relevance†
†Electronic supplementary information (ESI) available. See DOI: 10.1039/c7sc05009g


**DOI:** 10.1039/c7sc05009g

**Published:** 2018-02-19

**Authors:** Kjell Jorner, Burkhard O. Jahn, Patrick Bultinck, Henrik Ottosson

**Affiliations:** a Department of Chemistry – Ångström Laboratory , Uppsala University , Box 523 , 751 20 Uppsala , Sweden . Email: henrik.ottosson@kemi.uu.se; b SciClus GmbH & Co. KG , Moritz-von-Rohr-Str. 1a , 07745 Jena , Germany; c Department of Chemistry , Ghent University , Krijgslaan 281 (S3) , 9000 Gent , Belgium . Email: Patrick.Bultinck@UGent.be

## Abstract

Cyclic conjugation that occurs through-space and leads to aromatic properties is called homoaromaticity. Here we formulate the homoaromaticity concept for the triplet excited state (T_1_) based on Baird's 4*n* rule and validate it through extensive quantum-chemical calculations on a range of different species (neutral, cationic and anionic). By comparison to well-known ground state homoaromatic molecules we reveal that five of the investigated compounds show strong T_1_ homoaromaticity, four show weak homoaromaticity and two are non-aromatic. Two of the compounds have previously been identified as excited state intermediates in photochemical reactions and our calculations indicate that they are also homoaromatic in the first singlet excited state. Homoaromaticity should therefore have broad implications in photochemistry. We further demonstrate this by computational design of a photomechanical “lever” that is powered by relief of homoantiaromatic destabilization in the first singlet excited state.

## Abstract 2

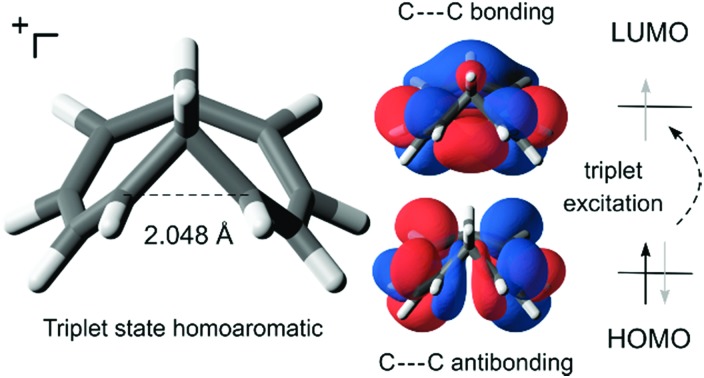
Conjugation through space can give rise to aromaticity in the lowest excited triplet state, with impact for photochemistry.

## Introduction

Excited-state aromaticity is a concept describing the energetic stabilization of annulenes with 4*n* π electrons in the lowest ππ* electronically excited states of singlet (S_1_) and/or triplet (T_1_) multiplicity. Originally conceived on a theoretical basis by Baird in 1972 1 based on preliminary work by Dewar and Zimmerman,^2^ the concept has lately been applied experimentally to rationalize various excited state properties and reactivity.^3^ The findings on excited-state aromaticity are now conveniently summarized in Baird's rule: 4*n* π-electron annulenes are aromatic in their S_1_ and T_1_ states while (4*n* + 2)π-electron annulenes are antiaromatic. Thus, Baird's rule is the excited state counterpart of Hückel's rule for the electronic ground state (S_0_), yet, the electron counts for aromaticity and antiaromaticity in the T_1_ and S_1_ states are the exact opposite to those in the S_0_ state. Excited state aromaticity has been used to explain acid–base properties of polycyclic conjugated hydrocarbons,^4^ excitation energies of substituted fulvenes,^5^ and spectroscopic properties of expanded porphyrinoids.^6^–^8^ Recently, the concept has been applied to the development of new photochemical reactions such as the formation of benzofulvenes from enynes,^9^ and photohydrogenation and photo(hydro)silylation of small polycyclic aromatic hydrocarbons and graphene.^10^ Large conformational changes have been observed upon excitation of annulenes, leading to planarization for excited-state aromatic and puckering for antiaromatic molecules.^7^,^11^

**Fig. 1:**
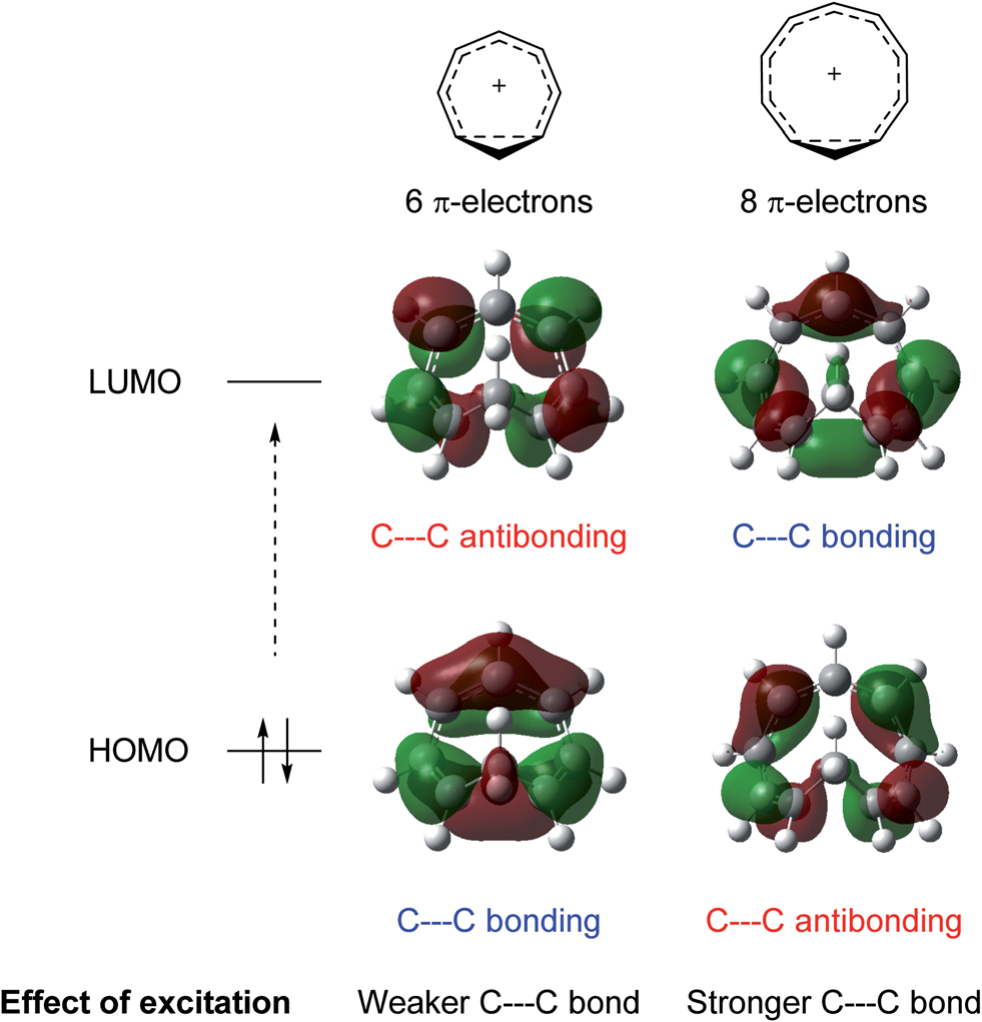
Effect of excitation on cyclically homoconjugated systems with six and eight π-electrons. Molecular orbitals obtained at the B2PLYP/6-311+G(d,p) level (isosurface value = 0.02).

While excited-state aromaticity is a powerful concept for understanding properties and reactivity following excitation, its influence has almost exclusively been considered for conventional planar annulenes. One exception concerns Möbius aromaticity, where computations predicted that excited (4*n*)π-electron annulenes should be antiaromatic and (4*n* + 2)π-electron annulenes aromatic,^12^ and this was recently confirmed experimentally.^8^,^13^ In this study, we focus on homoaromaticity, *i.e.*, aromaticity due to interaction of π orbitals over a formally saturated center,^14^ and show that this concept is applicable also in the excited triplet state. This can actually be anticipated from a simple analysis of the HOMO and LUMO orbitals of the aromatic homotropylium cation with six π-electrons and its larger analogue with eight π-electrons. Excitation should decrease the through-space conjugation of the six-electron system and enhance that of the eight-electron system, as electrons are excited from a bonding to an antibonding orbital for the former and from an antibonding to a bonding orbital for the latter (Fig. 1).

**Scheme 1:**
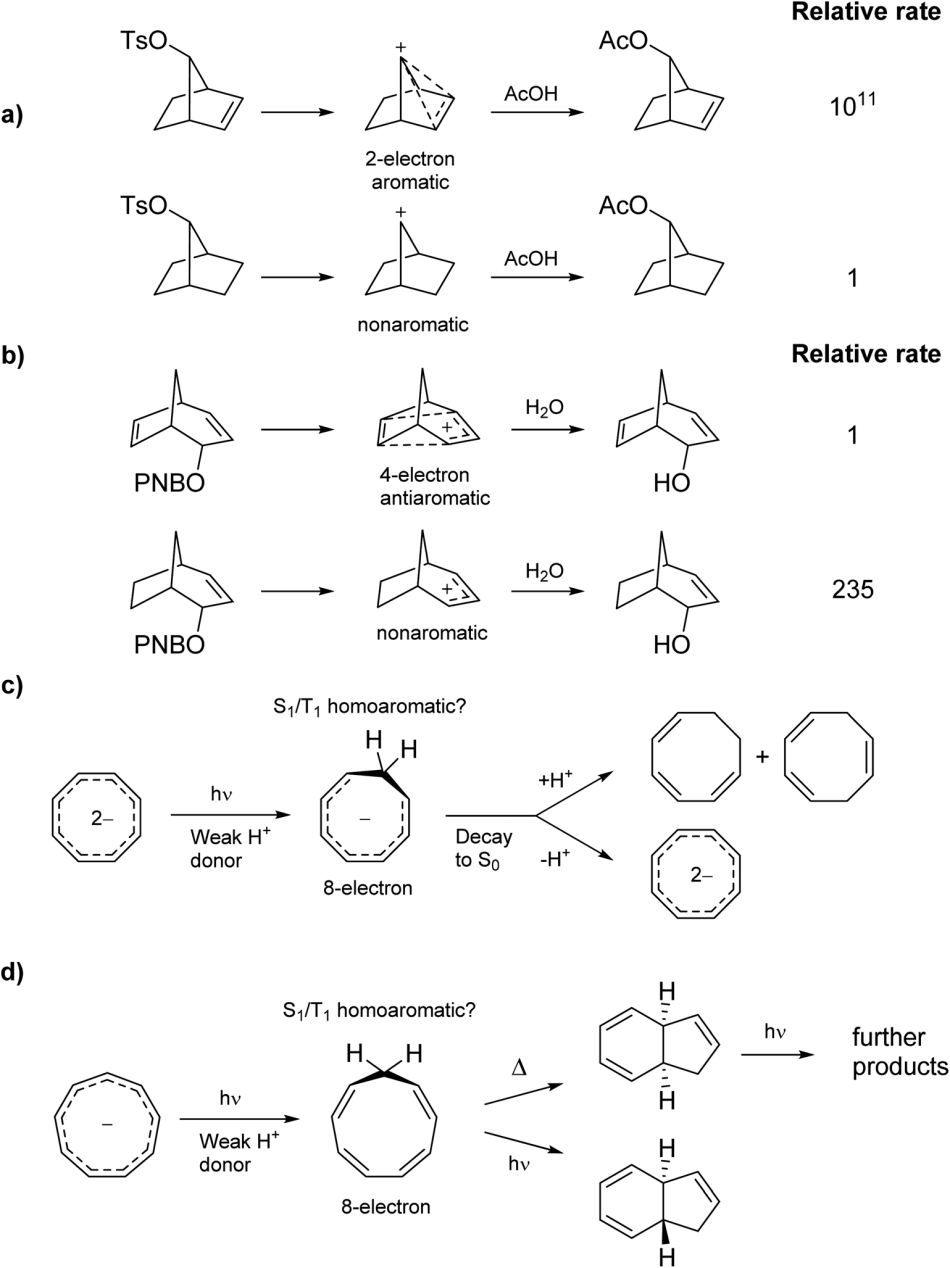
Ground state (a) homoaromatic stabilization and (b) homoantiaromatic destabilization. Tentatively excited state homoaromatic intermediates (c and d).

Homoaromaticity was coined by Winstein in 1959 to explain structures of non-classical carbocations.^15^ It refers to the appearance of aromatic properties through conjugation over saturated centers. One example is found in the acetolysis of the *p*-toluenesulfonate of 7-norborneol in which the cationic center of the intermediate conjugates through space with a double bond, creating a 2-electron aromatic cycle (Scheme 1a). The special stability of this cation is reflected in the rate enhancement of 10^11^ compared to the saturated analogue. Conversely, the special instability of homoantiaromatic 4π-electron cations was shown in the solvolysis of bicyclo[3.2.l]octa-2,6-dienyl *p*-nitrobenzoates where the saturated species reacted 235 times faster than the unsaturated (Scheme 1b).^16^,^17^ Perhaps the most well-known homoaromatic molecule is the homotropylium cation, which can be formed by protonation of cyclooctatetraene (COT) in strong acids such as H_2_SO_4_.^18^ Experimentally, homoaromaticity is also important for understanding the properties of substituted fullerenes^19^ and many inorganic molecules and clusters.^20^

While it is established that homo(anti)aromaticity can influence ground state reactions, its effect in the excited state is completely unexplored. However, we now postulate that many photochemical reactions can be influenced by homoaromaticity in S_1_/T_1_. For example, the photo-acidity of the cyclooctatetraene dianion is enhanced in the excited state, leading to the protonated cyclooctatrienyl anion as an excited state intermediate (Scheme 1c).^21^ Is this species excited-state homoaromatic? A similar reaction concerns the cyclononatetraenide anion which displays increased basicity in the excited state, being protonated to give cyclononatetraene as an intermediate to further products (Scheme 1d).^22^ In our earlier study of the norbornadiene–quadricyclane photo-switch system, we computationally found an excited-state minimum that we described as excited-state homoaromatic.^23^ These three examples indicate that excited state homoaromaticity may influence the outcome of photochemical reactions by stabilizing excited state intermediates. Now, through an extensive investigation of a number of a different species, possible in a facile manner only through quantum chemical computations, we find that homoaromaticity is indeed valid also for the excited state. A thorough understanding of excited state homoaromaticity could lead to the development of novel photochemical reactions, tuning of photophysical properties by appropriate choice of substrates and substituents, and design of new optically active molecular machinery.

## Results and discussion

**Fig. 2:**
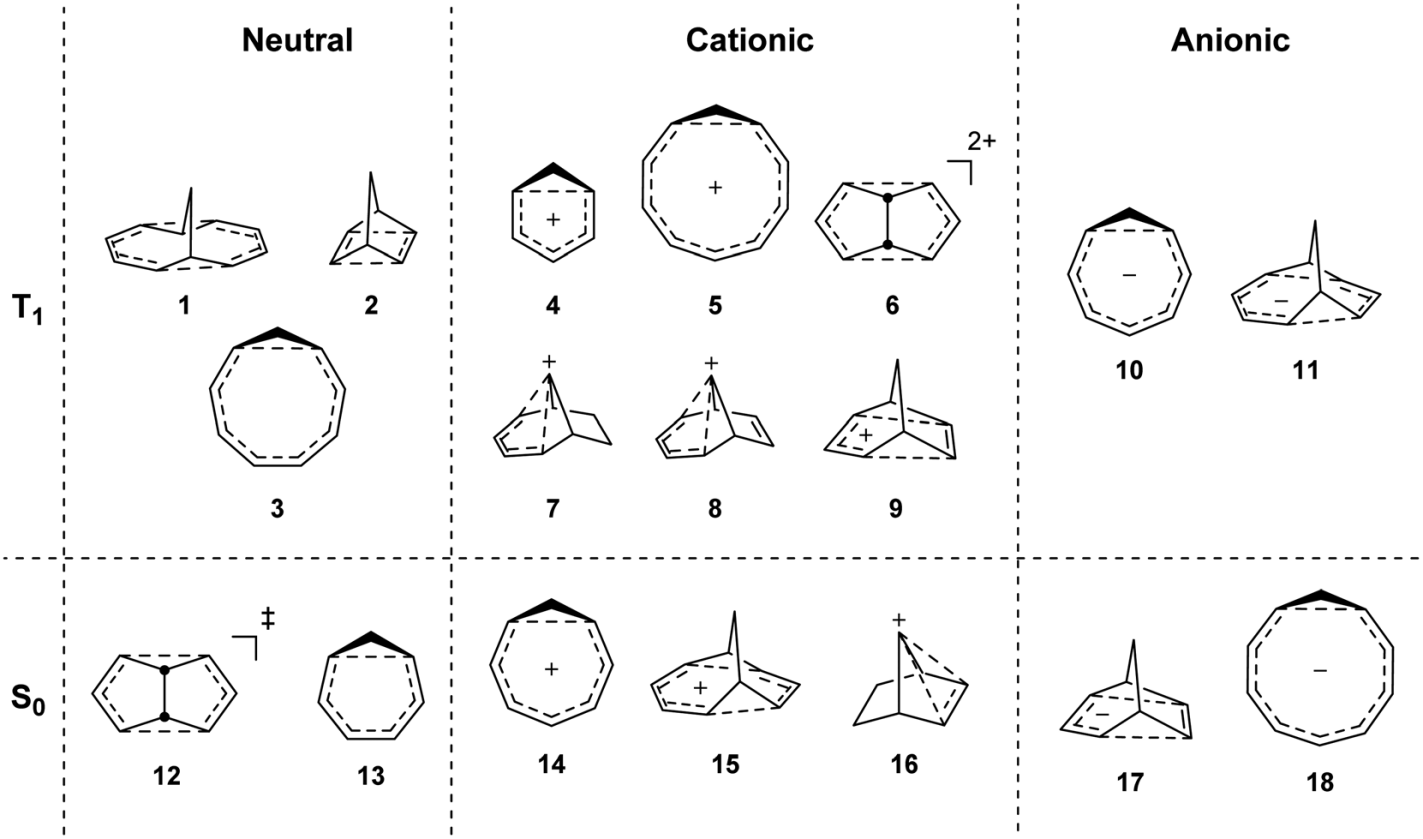
Studied compounds in T_1_ (**1–11**) and in S_0_ (**12–17**).

To investigate if homoaromaticity is influential in the T_1_ state, we computationally analyzed a series of neutral, cationic and anionic compounds (Fig. 2). We compared possible T_1_-homoaromatic structures (**1–11**) with compounds that are either experimentally established homoaromatics in S_0_ (**12–16**)^14^,^24^,^25^ or those whose homoaromaticity is so far only supported computationally (**17–18**).^26^,^27^ Although **12** is a transition state and not a stable compound, there have been extensive attempts to make derivatives in which this homoaromatic structure is a true energy minimum.^28^ While the T_1_-homoaromatic compounds all contain 4*n* π-electrons in the conjugated cycle, the S_0_ homoaromatic compounds contain (4*n* + 2)π-electrons.

Any rigorous assessment of homoaromaticity should include several different aspects.^14^ For the geometric aspect, we analyze the degree of the bond length alternation (BLA) and the distance of the though-space conjugative linkages (*r*(C···C)). For the electronic aspect, we look at the strength of the through-space and cyclic conjugation through the Wiberg bond indices^29^ and the multicenter indices,^30^ respectively. For the magnetic aspect, we use the anisotropy of the induced current density (ACID) plots^31^ and nucleus-independent shift (NICS)^32^ scans,^33^ and for the energetic aspect we look at aromatic stabilization energies using the ISE method.^34^ For the charged compounds we analyze charge delocalization and for the triplet state compounds also spin delocalization. We take care to compare the neutral, cationic and anionic compounds in S_0_ and T_1_ within each charge class as in the S_0_ state it is known that homoaromaticity is stronger in cationic than neutral or anionic compounds.^14^ Finally, we show how homoaromaticity could influence the photochemistry when extending the results to the S_1_ excited state.

### Geometries

**Fig. 3:**
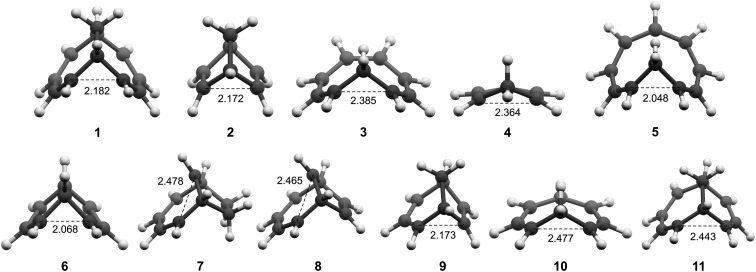
Optimized structures of **1–11** in T_1_.

All molecules were optimized at the B2PLYP/6-311+G(d,p) level, and here we include only the homoaromatic structures for each molecule. For the bicyclic systems **1**, **2**, **7** and **8**, we have found other conformers which are of similar or lower energy (see ESI,† Sections 5 and 12). For **5**, we found two homoaromatic conformers. Here we discuss only the one which is lowest in energy. The optimized structures are non-planar with short *r*(C···C) in the range 2.048–2.478 Å (Fig. 3). Note that non-planarity does not preclude aromaticity as exemplified by **14**, which is puckered and known to be strongly aromatic (see ESI,† Section 5). In particular, the optimized T_1_-structures of **1–11** show greater planarization and shorter *r*(C···C) in T_1_ than their optimized structures in S_0_.

### Bond length alternation

A low BLA is hallmark of both conventional aromaticity^35^ and homoaromaticity.^14^ The average and maximum BLAs (BLA_avg_ and BLA_max_, respectively) are given in Table 1 for the sp^2^-carbon framework of the monocyclic compounds. The BLAs for the T_1_ compounds are low and comparable in size to those in the S_0_ homoaromatic compounds (homoaromatics). Neutral **3** has a BLA_avg_ of 0.045 Å and a BLA_max_ of 0.070 Å, which are even smaller than the 0.085 Å and 0.092 Å for **13** in S_0_. Among the charged T_1_ species, BLA_avg_ and BLA_max_ are in the ranges 0.005–0.014 Å for cationic and 0.009–0.022 Å for anionic species, which is equal or lower to that of the established S_0_ homoaromatics (0.003–0.017 Å and 0.030–0.059 Å, respectively). Thus, the BLAs in the T_1_ monocyclic compounds are comparable or smaller than for the S_0_ homoaromatics, supporting their T_1_ homoaromaticity.

**Table 1:** Average and maximum bond length alternation (BLA) in Å for the monocyclic compounds. Results at the B2PLYP/6-311+G(d,p) level

Compound	Charge	Electronic state	BLA_avg_	BLA_max_
**3**	0	T_1_	0.045	0.070
**4**	+1	T_1_	0.009	0.009
**5**	+1	T_1_	0.005	0.014
**10**	–1	T_1_	0.009	0.022
**13**	0	S_0_	0.085	0.092
**14**	+1	S_0_	0.003	0.017
**18**	–1	S_0_	0.030	0.059

### C···C homoconjugative distances

**Fig. 4:**
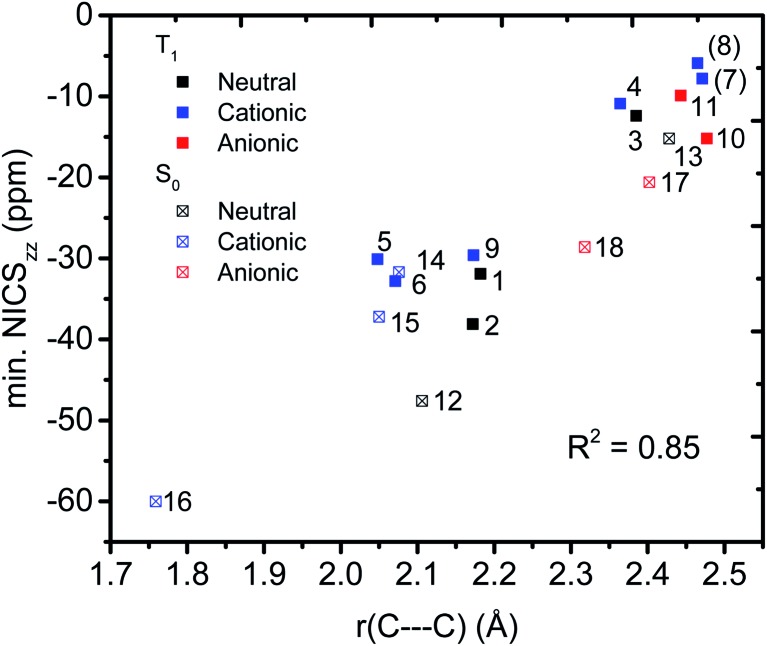
Correlation between *r*(C···C) and minimum NICS_*zz*_ values along the NICS scan.

We then evaluated the *r*(C···C) distances as a measure of the strength of the homoconjugation, with shorter distances being expected for stronger homoconjugation. In Fig. 4 we plot *r*(C···C) against minimum NICS_*zz*_ values along the NICS scan as a magnetic measure of aromaticity. A clear correlation is observed with both S_0_ and T_1_ and cationic compounds having shorter *r*(C···C) and more negative NICS_*zz*_ than anionic or neutral compounds. Still, for all compounds *r*(C···C) is smaller than the sum of two carbon van der Waals radii (3.40 Å).^36^ The T_1_ compounds are fully comparable to their S_0_ analogues, with stronger conjugation and homoaromaticity expected for **1**, **2**, **5**, **6** and **9** and weaker conjugation and homoaromaticity expected for **3**, **4**, **7**, **8**, **10** and **11**. The exceptions are **7** and **8** that show much larger *r*(C···C) than their closest homoaromatic analogue in S_0_ (**16**).

### Wiberg bond indices

**Fig. 5:**
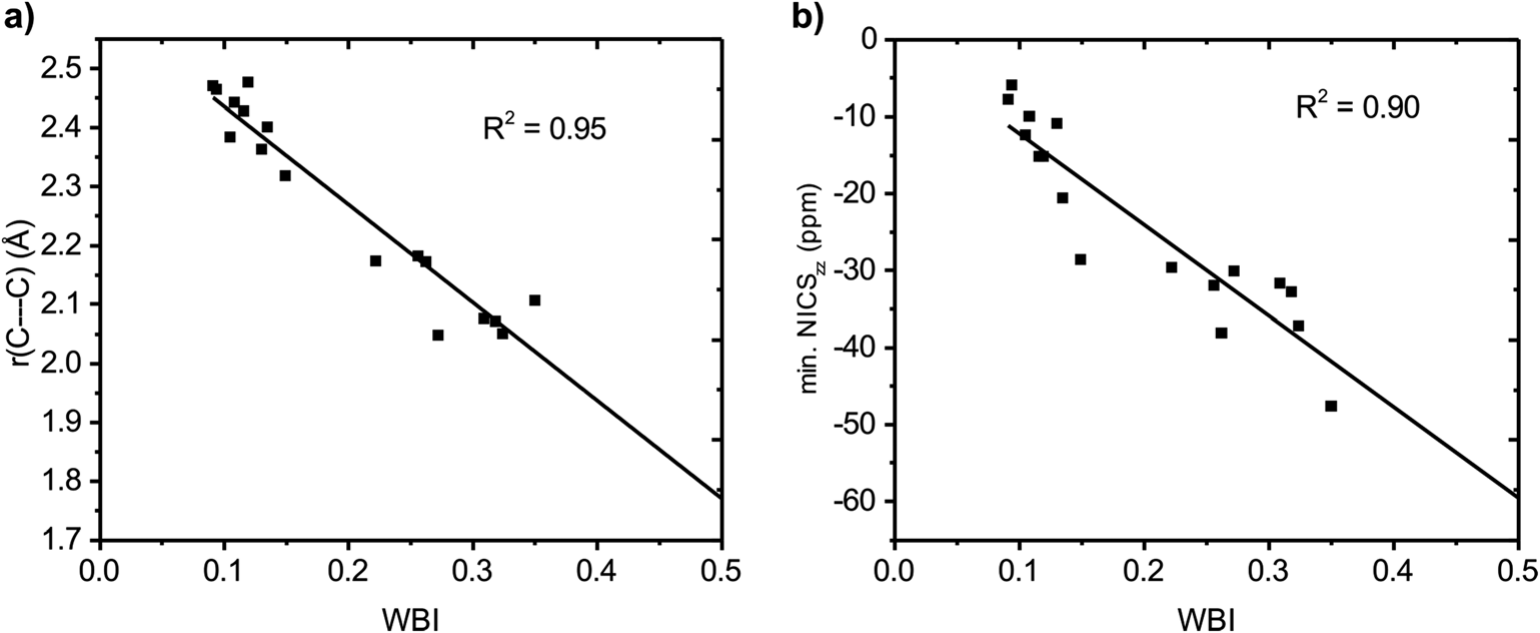
Correlation between Wiberg bond index (WBI) and (a) C···C distance and (b) minimum NICS_*zz*_ value along the NICS scan.

We further quantified the strength of the through-space conjugation by the Wiberg bond indices (WBI).^29^ The WBI values (Table 2) correlate well with *r*(C···C) for all compounds (**1–18**), showing that smaller *r*(C···C) are indeed associated with increased through-space conjugation (Fig. 5a).

**Table 2:** C···C distances, Wiberg bond indices, MCI, standard deviation of NPA charges and spin densities for C atoms (with attached hydrogens) in the homoaromatic circuit, ring current directions according to the ACID plots and minimum NICS values

	State	Charge	*r*(C···C)^*a*^ (Å)	WBI^*a*^	MCI^*a*^	*σ*_Q_ ^*a*^	*σ*_SD_ ^*a*^	Ring current^*b*^	Min. NICS^*b*^
**1**	T_1_	0	2.182	0.256	0.645	—	0.115	Diatropic	–31.9
**2**	T_1_	0	2.172	0.262	0.626	—	0.000	Diatropic	–38.1
**3**	T_1_	0	2.385	0.105	0.537	—	0.363	Diatropic	–12.4
**4**	T_1_	+1	2.364	0.130	0.568	0.116	0.248	Not clear	–10.9
**5**	T_1_	+1	2.048	0.272	0.650	0.084	0.140	Diatropic	–30.1
**6**	T_1_	+1	2.071	0.318	0.663	0.044	0.013	Diatropic	–32.8
**7**	T_1_	+1	2.471/2.478	0.091	0.207	0.138	0.373	Not clear	–7.8
**8**	T_1_	+1	2.465	0.094	0.060	0.141	0.384	Not clear	–5.9
**9**	T_1_	+1	2.173	0.222	0.597	0.083	0.222	Diatropic	–29.6/–28.6
**10**	T_1_	–1	2.477	0.119	0.565	0.090	0.264	Diatropic	–15.2
**11**	T_1_	–1	2.443	0.108	0.481	0.095	0.326	Not clear	–9.9
**12**	S_0_	0	2.106	0.350	0.675	—	—	Diatropic	–47.6
**13**	S_0_	0	2.428	0.116	0.581	—	—	Diatropic	–15.2
**14**	S_0_	+1	2.076	0.309	0.687	0.074	—	Diatropic	–31.7
**15**	S_0_	+1	2.050	0.324	0.669	0.099	—	Diatropic	–37.2
**16**	S_0_	+1	1.759	0.518	0.708	0.015	—	Diatropic	–60.0
**17**	S_0_	–1	2.402	0.135	0.525	0.135	—	Not clear	–20.6
**18**	S_0_	–1	2.318	0.149	0.611	0.062	—	Diatropic	–28.6

^*a*^At the B2PLYP/6-311+G(d,p) level.

^*b*^At the B3LYP/6-311+G(d,p)//B2PLYP/6-311+G(d,p) level.

The positively charged S_0_ state homoaromatics have WBI values in the range 0.309–0.518, indicating very strong homoconjugation, while the anionic compounds have significantly lower values of 0.135–0.149. For comparison, π-WBIs of fully aromatic bonds lie in the range 0.220–0.428 (S_0_ values; see Table S1, ESI†). For neutral **13**, a rather small value of 0.116 is obtained, indicating only weak homoconjugation. Among the positively charged T_1_ homoaromatics, **5**, **6** and **9** show strong homoconjugation on par with **14** in S_0_. Cation **4** has moderate conjugation, while that in **7** and **8** is weak. The anionic **10** and **11** show moderate homoconjugation only slightly lower than that of **17** and **18** in S_0_.

### Multicenter indices

**Fig. 6:**
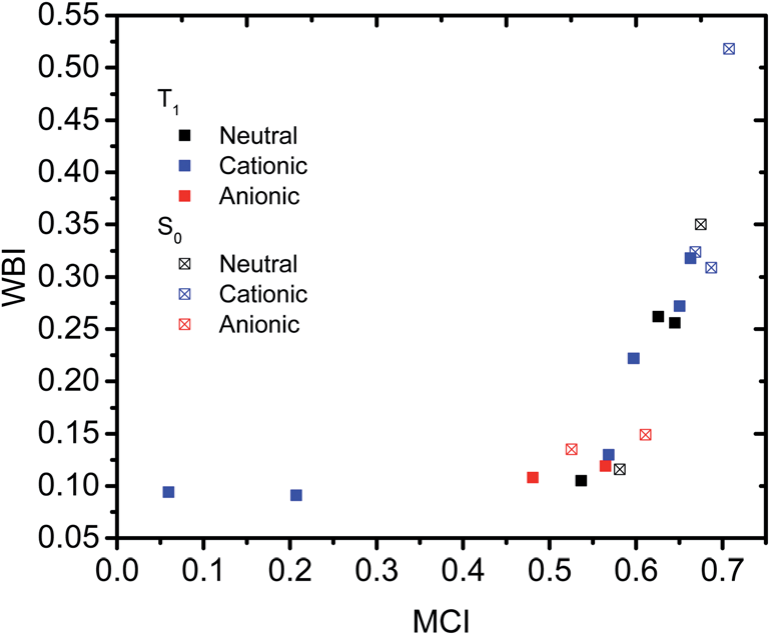
Relationship between MCI and WBI(C···C).

**Fig. 7:**
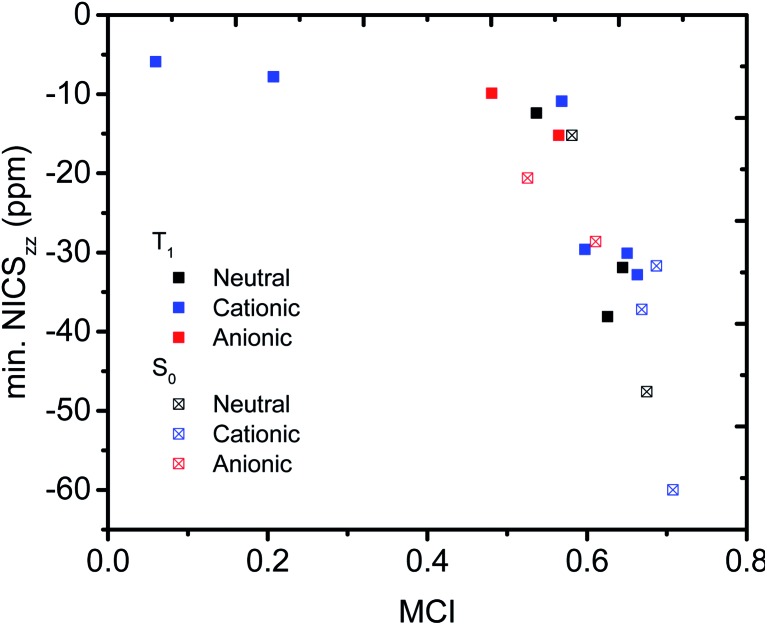
Relationship between MCI and minimum NICS_*zz*_ value along the NICS scan.

Having established that a short *r*(C···C) is indeed related to stronger through-space conjugation, as evidenced by the WBI(C···C), we went on and calculated the multicenter indices (MCI)^30^ to verify the existence of larger cyclic conjugation. The MCI quantifies the extent of delocalized cyclic bonding and has been used previously to assess homoaromatic species in the S_0_ state.^37^ To compare the homoaromaticity of rings of different size, we employed the normalization procedure by Mandado *et al.*^38^ Henceforth, MCI therefore refers to the normalized MCI index. For full MCI values, see Table S2, ESI.† The MCI values correlate with the WBI(C···C) ones in a non-linear fashion with no clear difference between S_0_ and T_1_ compounds (Fig. 6). However, according to MCI the aromatic character of **7** (0.207) and **8** (0.060) is much lower than the rest of studied compounds (range of 0.481–0.708) and they are best considered non-aromatic. The correlation of MCI with NICS is similarly non-linear (Fig. 7). In summary, the MCI calculations support varying extent of homoaromatic character of all compounds except **7** and **8** and allow a quantitative ordering that will be discussed further in the Conclusions section.

### Charge and spin distribution

Delocalization of charge is often employed to assess the homoaromaticity of charged species in the S_0_ ground state.^14^ The prime example is the homotropylium cation (**14**), in which charge delocalization is evidenced by the small variation in ^13^C NMR shifts.^39^ To assess this property we calculated atomic charges using the natural population analysis (NPA) scheme^40^ and computed the standard deviation of the charge (*σ*_Q_) for the unsaturated carbon atoms in the homoaromatic circuit. The results in Table 2 show that **4–6** and **9–11** in their T_1_ states have similar charge delocalization as the S_0_ state homoaromatics. Compounds **7** and **8** show high charge localization and should thus be only weakly homoaromatic or non-aromatic. The charge localization in **17** (*σ*_Q_ = 0.135) is also quite high. For the cationic species in the T_1_ state there is a clear correlation between *σ*_Q_*vs.* WBI(C···C) (Fig. S2a, ESI,† *R*^2^ = 0.93) and minimum NICS_*zz*_ values (Fig. S2c, ESI,† *R*^2^ = 0.88), but if one considers all positively and negatively charged compounds in both the S_0_ and T_1_ states, the correlations become worse. The correlation between MCI and *σ*_Q_ is non-linear (Fig. S2b, ESI†).

For the T_1_ state we can also quantify the delocalization of excess spin, and we expect that more homoaromatic molecules show larger spin delocalization with smaller standard deviations (*σ*_SD_). Indeed, *σ*_SD_ (Table 2) is correlated to both WBI(C···C) (Fig. S3a, ESI,† *R*^2^ = 0.88) and the minimum NICS_*zz*_ values (Fig. S3b, ESI,† *R*^2^ = 0.86), showing that the delocalization of spin in the T_1_ state is strongly associated with the extent of homoconjugation and homoaromaticity. Correlation between MCI and *σ*_SD_ is again non-linear (Fig. S3b, ESI†). Figures with the spin densities are given in the ESI,† Section 10.

### ACID plots

**Fig. 8:**
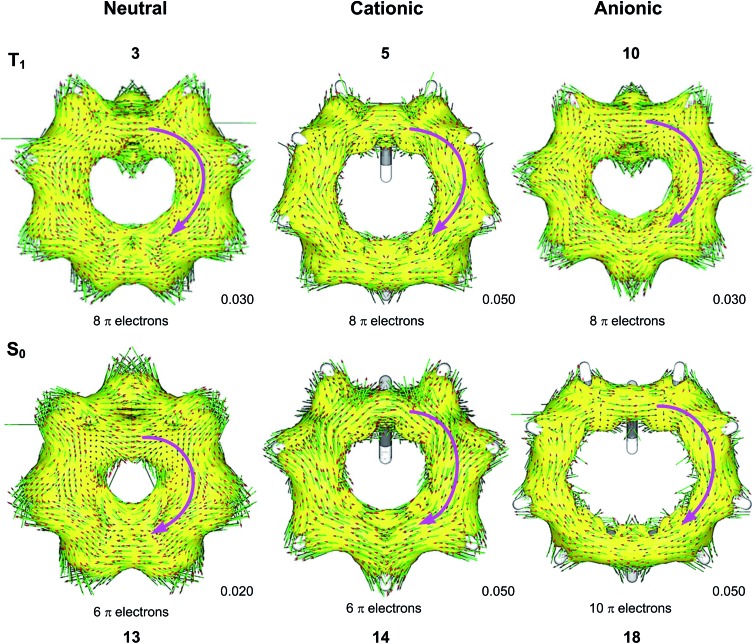
ACID plots with B3LYP/6-311+G(d,p) for selected compounds in T_1_ and S_0_. Clockwise arrows indicate a diatropic ring current indicative of aromaticity. ACID isosurface values are indicated in the figure.

We used the ACID method to visualize the ring current associated with homoaromaticity. Ring currents have previously been analyzed by Sundholm^41^ and Schleyer^25^ for homoaromatic compounds in the S_0_ ground state. In our plots, diatropic currents run clockwise and indicate aromaticity, while paratropic currents run counter-clockwise and indicate antiaromaticity. In Fig. 8, the ACID plots for a selection of compounds in T_1_ and S_0_ are shown. ACID plots for all compounds are given in Section 9 of the ESI,† and the direction of the ring current (diatropic or paratropic) is given in Table 2. The ACID plots in Fig. 8 identify that **3**, **5** and **10** are T_1_ homoaromatic, while **13**, **14** and **18** are S_0_ homoaromatic. Considering the strength of the ring currents (through the length of the arrows in the ACID plots), **14** appears to be most aromatic, while **3** and **13** have lower aromaticity. Notably, **5** displays a strong ring current and is clearly aromatic. All T_1_ compounds except **4**, **7**, **8** and **11** have visible ring currents in the ACID plots, supporting their T_1_ homoaromaticity.

### NICS scans

**Fig. 9:**
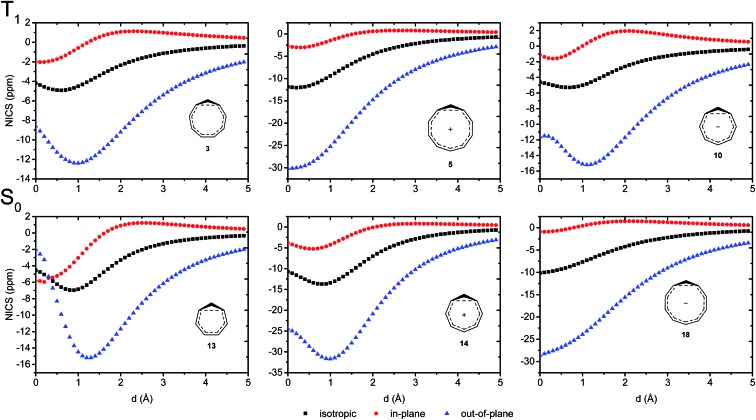
NICS scans of selected compounds in the T_1_ and S_0_ states. Deep minima in the out-of-plane component of the NICS indicate aromatic character.

We further probed the magnetic properties with the NICS scan method.^33^ Aromatic compounds are characterized by a relatively deep minimum in the out-of-plane component of the magnetic shielding tensor, while antiaromatic compounds have a maximum at 0 Å that goes to zero with increasing distance. Non-aromatic compounds have a shallow minimum. Although NICS has shown limitations for non-planar and polycyclic compounds,^42^ a previous study on homoaromatic compounds found a very good correlation between NICS and multicenter indices (*R*^2^ = 0.96).^37^ We also complement the NICS scans with ring current density plots (using ACID), as recommended in the recent literature.^43^ The NICS scans for a selection of compounds are given in Fig. 9, while NICS scans for all compounds are given in the ESI† and the minimum NICS_*zz*_ values along the scan are given in Table 2. As the direction of the NICS scan is not unambiguous for non-planar homoaromatic compounds, the placement of the bq probe atoms (along the *z*-axis of the coordinate system) are given graphically in Section 6 of the ESI† and also as separate coordinate files. As shown for the *B*_ind_ aromaticity index, the direction of the *z*-axis in magnetic calculations is important.^44^ We have therefore also calculated three-dimensional iso-chemical shielding surfaces (ICSSs)^45^ for a selection of compounds (Section 15 of the ESI†), and the results show that small deviations in scan direction or origin do not affect the interpretations. From Fig. 9 we see that **3** has a minimum of nearly the same depth as **13** (–12.4 *vs.* –15.2 ppm), **5** has a minimum nearly as deep as **14** (–30.1 *vs.* –31.7 ppm) and **10** has a shallower minimum than **18** (–15.2 *vs.* –28.6 ppm). Overall, the NICS scans support strong homoaromaticity of **1**, **2**, **5**, **6** and **9** in the T_1_ state as well as **12** and **14–18** in the S_0_ state. Compounds **3**, **4**, **10**, **11** and **13** have weaker aromaticity and **7** and **8** are best characterized as non-aromatic. As noted above, the minimum NICS values along the scan are well-correlated to the WBI(C···C) and MCI values, indicating that it is indeed the increased through-space conjugation and aromaticity that leads to the lower NICS values (Fig. 5b and 7).

### ISE values

**Scheme 2:**
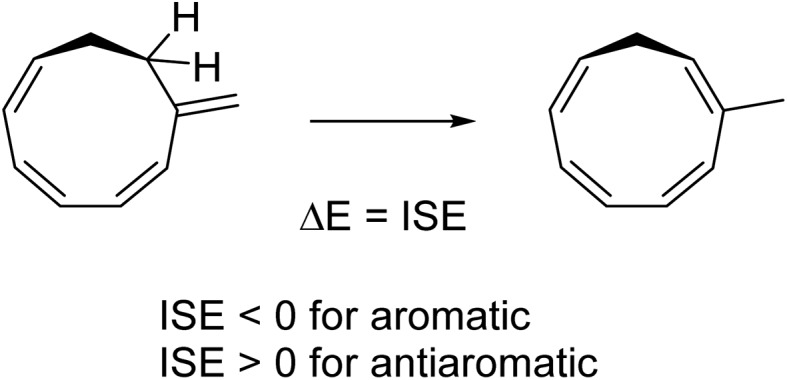
Example reaction for determining the ISE values.

To assess the energetic stabilization due to homoaromaticity we used the isomerization stabilization energy (ISE) method.^34^ The ISE method computes the aromatic stabilization energy by comparing a methylated aromatic compound to its non-aromatic exocyclic methylene isomer (Scheme 2). Typical homoaromatic stabilization energies in the ground state are small and in the range 2–10 kcal mol^–1^ and therefore easily offset by destabilizing strain.14*b* Therefore, we focus on the difference ΔISE = ISE(T_1_) – ISE(S_0_) for which strain effects should largely cancel between the two electronic states. We expect positive ΔISE values for S_0_-homoaromatic species (S_0_-homoaromatic → T_1_-nonaromatic) and negative ΔISE values for T_1_-homoaromatic species (S_0_-nonaromatic → T_1_-homoaromatic).

Indeed, the S_0_-homoaromatic compounds **13–18** display positive ΔISE values as expected (Table 3). Compounds **1–11** show the expected negative values except for **4** and **7**. The homoaromaticity of **7** is questionable and weak at best according to the geometric, electronic and magnetic criteria (*vide supra*). It is therefore not surprising that it is not aromatic according to the energetic criterion. For **4** the unexpected positive value is due to hyperconjugative aromaticity which stabilizes the S_0_ state (the electrons of the C–H bonds of the saturated center take part in the conjugation, giving a 6π system).^46^ Interestingly, **4** could be seen as an aromatic chameleon^47^ which is 6 π-electron hyperconjugatively aromatic in the S_0_ state and 4 π-electron homoaromatic in the T_1_ state. In summary, we conclude that the ISE method supports the homoaromatic character of **1–3**, **5**, **6** and **8–11** while **7** is considered non-aromatic and the situation is not clear for **4**.

**Table 3:** ISE values in kcal mol^–1^ for the S_0_ and T_1_ states at the B2PLYP/6-311+G(d,p) level^*a*^

Compound	ISE(S_0_)	ISE(T_1_)	ΔISE^*a*^
**1**	–1.3	–6.8	–5.5
**2**	+6.6	+3.4	–3.2
**3**	–0.2	–4.0	–3.8
**4**	–11.6	–5.0	+6.6
**5**	–3.0	–6.5	–3.5
**6**	–0.7	–6.5	–5.8
**7**	–10.0	–7.7	+2.3
**8**	–0.4	–4.0	–3.6
**9**	+2.9	–12.7	–15.5
**10**	+4.1	–4.3	–8.4
**11**	+2.2	–3.2	–5.4
**12**	n/a^*b*^	n/a^*b*^	n/a^*b*^
**13**	–4.0	–3.2	+0.8
**14**	–15.0	–1.6	+13.3
**15**	–13.8	–1.7	+12.1
**16**	–21.6	–5.1	+16.5
**17**	–2.1	+2.2	+4.3
**18**	–5.9	+4.3	+10.3

^*a*^Negative values indicate homoaromatic stabilization in T_1_ while positive values indicate loss of homoaromatic stabilization in S_0_. Values close to zero are characteristic of non-aromaticity.

^*b*^ **12** was not included as it is a TS structure that cannot be treated with the method.

### Extension to the S_1_ state

After having thoroughly assessed homoaromaticity in T_1_, we extend the concept also to the S_1_ state. We turn to **3** and **10** that were identified as possible excited state intermediates (Scheme 1c and d). Although we have shown that they are homoaromatic in the T_1_ state, it is likely that the photoreactions described occur in the S_1_ state. Therefore, we optimized **3** and **10** in the S_1_ state using CASSCF/6-31G(d). For **10** we could also employ TD-B3LYP/6-31+G(d,p), while this was not possible for **3** due to the doubly excited character of the S_1_ state. The optimized structure of **3** in S_1_ is similar to that in T_1_ but with a shorter *r*(C···C) of 2.119 Å in S_1_ with CASSCF compared to 2.385 Å in T_1_ with B2PLYP.

The *C*_2v_-symmetric optimized structure of **10** in S_1_ with *r*(C···C) = 2.379 Å is not stable with CASSCF and distorts to a more charge-localized *C*_1_-symmetric structure with *r*(C···C) = 2.360 Å. In T_1_ the structure is *C*_2v_-symmetric with *r*(C···C) = 2.477 Å according to B2PLYP. In contrast to CASSCF, TD-B3LYP favors the *C*_2v_-symmetric geometry with a *r*(C···C) = 2.495 Å which is more like the T_1_ value (2.477 Å). We conclude that **3** and **10** have significant homoconjugation in S_1_ based on their geometries.

So are they aromatic? Unfortunately, ACID plots are not available for the S_1_ state. However, qualitative NICS_iso_ scans in S_1_ show the characteristic aromatic minima, indicating that they are indeed aromatic (Fig. S9 and S10, ESI†).^48^ We therefore tentatively conclude that **3** and **10** are both T_1_- and S_1_-homoaromatic, although we defer an extended investigation into S_1_ homoaromaticity to a later study.

### Application to design of photomechanical materials

**Fig. 10:**
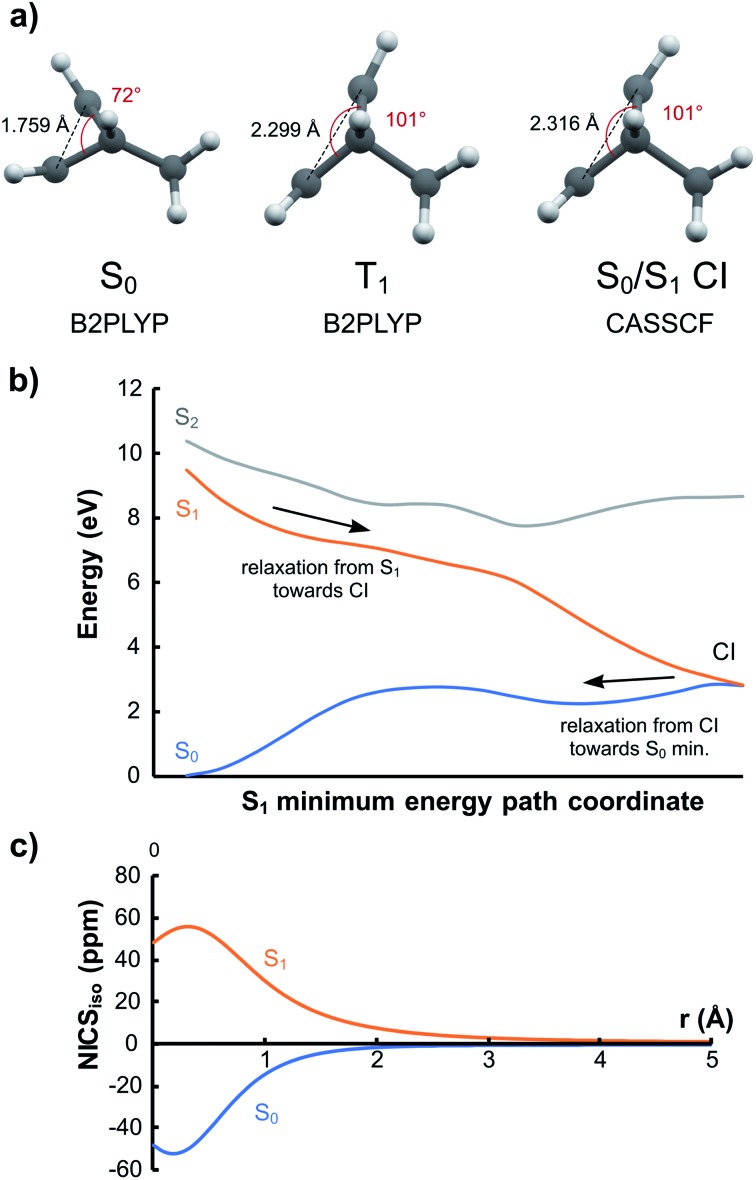
(a) Optimized geometries for **16** for S_0_, T_1_ and S_0_/S_1_ conical intersection. (b) CASSCF energies on minimum energy path in S_1_ following vertical excitation. (c) NICS_iso_ scans for S_0_ and S_1_ at the S_0_ geometry.

Compound **16** is especially interesting as it can function as a photomechanical lever. In S_0_ it prefers the conformation with the bridging carbon coordinated to the double bond due to homoaromatic stabilization. From the results above, we expect that this cycle with 2π electrons will become homoantiaromatic in the S_1_ and T_1_ states and that the bridging carbon will swing away from the ethylene segment. Indeed, optimization in T_1_ leads to a minimum with a considerably larger distance between the bridge and the bridging carbon (Fig. 10a). A minimum energy path calculation in S_1_ with CASSCF/6-31G(d) leads to a similar structure which is now a S_0_/S_1_ conical intersection (CI) leading back to the S_0_ minimum (Fig. 10b). The C–C–C angle is opened from 72° in S_0_ to 101° at the T_1_ minimum and S_0_/S_1_ CI. At the same time, *r*(C···C) increases from 1.759 Å in S_0_ to 2.299 Å in T_1_ and 2.316 Å at the CI. The S_0_, S_1_ and T_1_ states are almost degenerate at the CI as judged by MS-CASPT2 calculations (Fig. S6, ESI†).

The S_1_/T_1_ relaxation represents a substantial geometrical change that could be utilized as a photochemical “lever”. Exchanging the hydrogen substituent at the bridging carbon for larger groups would lead to an even higher torque. To confirm that homoantiaromaticity is indeed responsible for this conformational change, we calculated the NICS scan for the vertically excited S_1_ state. It shows a clear antiaromatic profile that is a mirror image of the aromatic scan in S_0_ (Fig. 10c). The T_1_ NICS scans at the vertically excited and relaxed T_1_ geometries shows that also the T_1_ state is antiaromatic but that the antiaromaticity is alleviated by de-coordination of the bridging carbon (Fig. S7 and S8, ESI†). Compound **16** could thus be a starting point for design of new photomechanical materials based on the concept of excited state homoantiaromaticity.

## Synopsis and outlook

The analysis of the different aromaticity criteria for **1–11** is summarized in Table 4, together with an overall assessment of the extent of T_1_ homoaromaticity. We find that **1**, **2**, **5**, **6** and **9** are strongly T_1_ homoaromatic, while **3**, **4**, **10** and **11** are weakly T_1_ homoaromatic. Compounds **7** and **8** are non-aromatic. It is thus established that homoaromaticity is important in the T_1_ excited state, and our calculations also indicated that this extends to the S_1_ state. Further investigations in this direction are currently ongoing in our laboratory. Electronic excitation emerges as a second way, besides a Möbius orbital topology,^49^ for 4*n*π systems to become homoaromatic. Based on the selected reactions from the literature with probable excited state homoaromatic intermediates (**3** and **10**), we expect that there are more photochemical mechanisms that can be interpreted using homoaromaticity. Furthermore, the concept should aid in the discovery and development of new photochemical reactions, as well as tailoring of photophysical properties. One example that we highlighted are the predicted photomechanical properties of **16** that are due to the homoantiaromatic destabilization in the excited state of the preferred ground state conformation. Thus, excited state homo(anti)aromaticity could be a new tool for design of novel optically active molecular machines.

**Table 4:** Summary of aromatic criteria and overall assessment of T_1_ state homoaromaticity of **1–11**. Full circle means that criterion is fulfilled, empty circle that it is not, dash that it could not be evaluated. Ranking of homoaromatic character is giving according to the MCI

#	BLA	*r*(C···C)	WBI(C···C)	*σ*_Q_	ACID	NICS	ΔISE	MCI	T_1_ homoaromaticity
**1**	—							3	Strong
**2**	—							4	Strong
**3**			○					8	Weak
**4**					○		—	6	Weak
**5**								2	Strong
**6**	—							1	Strong
**7**	—	○	○	○	○	○	○	10	Nonaromatic
**8**	—	○	○	○	○	○		11	Nonaromatic
**9**	—							5	Strong
**10**								7	Weak
**11**	—				○			9	Weak

## Computational methods

All calculations in the S_0_ and T_1_ states were performed using Gaussian 09 Revision D.01 and E.01 50 with the B2PLYP^51^ and B3LYP^52^ functionals and the 6-311+G(d,p) basis set.^53^ Restricted calculations were used for singlet states and unrestricted for triplet states. All compounds were optimized with B2PLYP/6-311+G(d,p) as it performed well against CCSD(T),^54^ BD(T)^55^ and CASPT2 56 results for a selection of compounds (see Section 11 of the ESI† for an extended discussion). Analysis of the CCSD T_1_ diagnostics and CASSCF configurational weights show that the triplet states are single-configurational and the use of DFT should therefore be appropriate (see ESI,† Section 13). Spin contamination of the T_1_ states is small at 2.01–2.05 except for two cases with 2.09 and 2.11. B2PLYP is insensitive to spin contamination of this magnitude.^57^ We did not include dispersion corrections beyond what is given by the MP2 component of B2PLYP, and calculations on a selection of the compounds show a very small effect on the obtained geometries (see Section 14 of the ESI†). Stationary points were confirmed by frequency calculations with no negative frequencies except for the TS structure of **12** with one negative frequency. The wavefunctions were checked for instabilities using the “stable” keyword in Gaussian 09. All NICS scans in T_1_ were generated with the Aroma β.4 package,^58^ using the GIAO method^59^ at the B3LYP/6-311+G(d,p) level as these open-shell magnetic calculations are not possible at the B2PLYP level. The placement of the ghost atoms is given in figures and included in the atomic coordinates in the ESI.† ACID calculations were performed using the AICD 2.0.0 program at the B3LYP/6-311+G(d,p) level using the CSGT method.^60^ We have chosen this level of theory to be consistent with the previous literature,^25^ and because ACID cannot be calculated at the B2PLYP level. Note that different ACID isosurface values were used to clearly identify the direction of the ring currents. ISE values were calculated at the B2PLYP/6-311+G(d,p) level. No *anti*–*syn* diene mismatch corrections were made. Atomic charges and spin densities were calculated with the natural population analysis scheme^40^ using NBO 6.0.^61^ Bond length alternation (BLA) was calculated according to the formula:
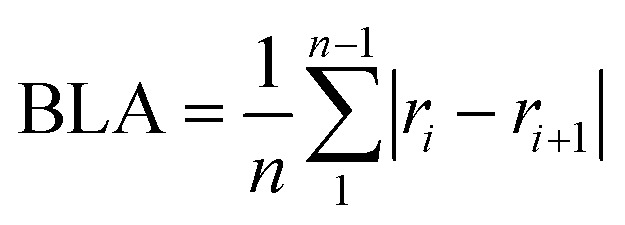


CASSCF optimization in the S_1_ states were performed with an active space of all π orbitals and electrons, a two-state (for **3** and **10**) or three-state (for **16**) average procedure and the 6-31G(d) basis set^62^ using a development version of Molcas 8.1.^63^ The smaller 6-31G(d) basis set was used to allow numerical frequency calculations in the S_1_ state. TD-DFT optimizations were done at the B3LYP/6-31+G(d,p) level with Gaussian 16 Rev. A.03.^64^ In both cases minima were confirmed by frequency calculations. NICS scans in S_1_ were calculated with Dalton 2016.0.^48^ WBI and multicenter indices were computed according to the equations reported previously^30^,^65^,^66^ and applied for the study of ground state homoaromaticity,^37^ including all permutations over the atomic labels as in several cases the sequential ordering was not the largest contributor to the MCI value. Bader's atoms in molecules approach,^67^ known as quantum chemical topology was used to distinguish the atoms in the molecule and to yield atomic overlap matrices using AIMAll.^68^ Orbitals from the DFT Kohn–Sham scheme were used in the same way as Hartree–Fock orbitals as experience has shown that this reveals the relevant chemistry properly.^69^ Normalization of the resulting indices was done as proposed by Mandado *et al.*^38^ for both spin contributions separately after which both were summed to yield a normalized total MCI. σ and π separation was done for planar conventional aromatics by limiting the sums over all orbitals to those relevant irreducible representations per contribution.

## Conflicts of interest

There are no conflicts to declare.

## Supplementary Material

Supplementary information

Supplementary information
