# Supplementary material for: Triplet state homoaromaticity: concept, computational validation and experimental relevance
Source: Chem Sci. 2018 Feb 19;9(12):3165–76. doi: 10.1039/c7sc05009g (PMC5916107; doi:10.1039/c7sc05009g)
Supplement: Supplementary file 1 [file SC-009-C7SC05009G-s001.pdf]

Electronic Supplementary Material (ESI) for Chemical Science.
This journal is © The Royal Society of Chemistry 2018

Contents

1 Conventional aromatic reference compounds 2
2 Multicenter indices 2
3 Charge and spin density distributions 3
4 ISE energies 4
5 Geometries 5
6 NICS scans 20
7 S_1 and T_1 calculations of compound 16 33
8 S_1 NICS scans 35
9 ACID plots 35
10 Spin densities 61
11 Method selection 66
11.1 Method evaluation for compound 2 66
11.1.1 Comparison between DFT and ab initio methods 66
11.1.2 Influence of multiconfigurational character 66
11.1.3 Effect of basis set for CASPT2 68
11.2 Method evaluation for compound 1 68
11.2.1 Comparison between DFT and ab initio methods 68
11.2.2 Influence of multiconfigurational character 69
11.3 Choice of functional 71
12 Energy differences with selected methods 71
13 Analysis of triplet wavefunctions 74
14 Effect of dispersion-corrected DFT 74
15 ICSS plots 75
16 Atomic coordinates and eneriges 76
17 References 76

1 Conventional aromatic reference compounds

For comparison, we give Wiberg bond indices, MCI values and minimum NICS values for compounds with conventional aromaticity (Figure S1, Table S1). The MCI values are consistent with those calculated by Mandado *et al.* for these systems.[1]

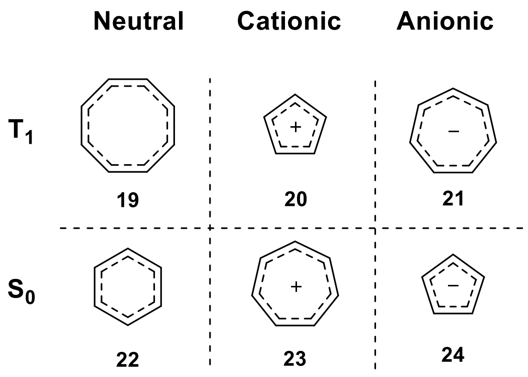

Figure S1. Reference aromatic molecules in T_1 (**19-21**) and S_0 (**22-24**).

Table S1. Wiberg bond indices, MCI values, ring current directions according to the ACID plots and minimum NICS values. The first two at the B2PLYP/6-311+G(d,p) level and the second two at the B3LYP/6-311+G(d,p) level.

	State	Charge	WBI	WBI_π	WBI_σ	MCI_α	MCI_β	MCI	Ring current	Min. NICS
**19**	T_1	0	1.347	0.304	1.043	0.325	0.361	0.686	Diatropic	-32.5
**20**	T_1	+1	1.263	0.177	1.085	0.347	0.371	0.718	Diatropic	-25.1
**21**	T_1	-1	1.339	0.309	1.030	0.284	0.361	0.644	Diatropic	-32.7
**22**	S_0	0	1.389	0.220	1.169	0.361	0.361	0.721	Diatropic	-29.2
**23**	S_0	+1	1.367	0.218	1.149	0.366	0.366	0.733	Diatropic	-26.4
**24**	S_0	-1	1.373	0.428	0.945	0.333	0.333	0.667	Diatropic	-33.9

2 Multicenter indices

Table S2. Non-normalized (subscript nn) and normalized MCI values at the B2PLYP/6-311+G(d,p) level.

	State	Charge	MCI_{nn}	$MCI_{\alpha,nn}$	$MCI_{\beta,nn}$	MCI_α	MCI_β	MCI
**1**	T_1	0	0.0019	0.0007	0.0012	0.3109	0.3339	0.6448
**2**	T_1	0	0.0796	0.0275	0.0521	0.2879	0.3379	0.6258
**3**	T_1	0	0.0004	0.0002	0.0003	0.2586	0.2780	0.5366
**4**	T_1	+1	0.0197	0.0135	0.0062	0.3062	0.2621	0.5684
**5**	T_1	+1	0.0007	0.0004	0.0003	0.3296	0.3208	0.6504
**6**	T_1	+1	0.0162	0.0097	0.0065	0.3426	0.3204	0.6631
**7**	T_1	+1	0.0006	0.0006	0.0000	0.1668	0.0405	0.2073
**8**	T_1	+1	0.0008	0.0009	-0.0001	0.1799	-0.1203	0.0596
**9**	T_1	+1	0.0238	0.0124	0.0114	0.3011	0.2961	0.5972
**10**	T_1	-1	0.0026	0.0004	0.0021	0.2501	0.3147	0.5647
**11**	T_1	-1	0.0008	0.0001	0.0006	0.2152	0.2654	0.4806
**12**	S_0	0	0.0177	0.0089	0.0089	0.3374	0.3374	0.6749
**13**	S_0	0	0.0072	0.0036	0.0036	0.2906	0.2906	0.5811
**14**	S_0	+1	0.0079	0.0040	0.0040	0.3435	0.3435	0.6870
**15**	S_0	+1	0.0065	0.0033	0.0033	0.3343	0.3343	0.6686
**16**	S_0	+1	0.2656	0.1328	0.1328	0.3538	0.3538	0.7075
**17**	S_0	-1	0.0125	0.0063	0.0063	0.2627	0.2627	0.5254
**18**	S_0	-1	0.0004	0.0002	0.0002	0.3054	0.3054	0.6108

3 Charge and spin density distributions

The standard deviation (σ_Q) of the charge is well-correlated with WBI (Figure S2a) and minimum NICS (Figure S2c) for cationic species but not for anionic species. The standard deviation (σ_D) of the spin density is well-correlated with WBI (Figure S3a) and minimum NICS (Figure S3c) for all species considered. For MCI we did not attempt linear correlation, but the properties seem to be non-linearly correlated (Figure S2b, Figure S3b).

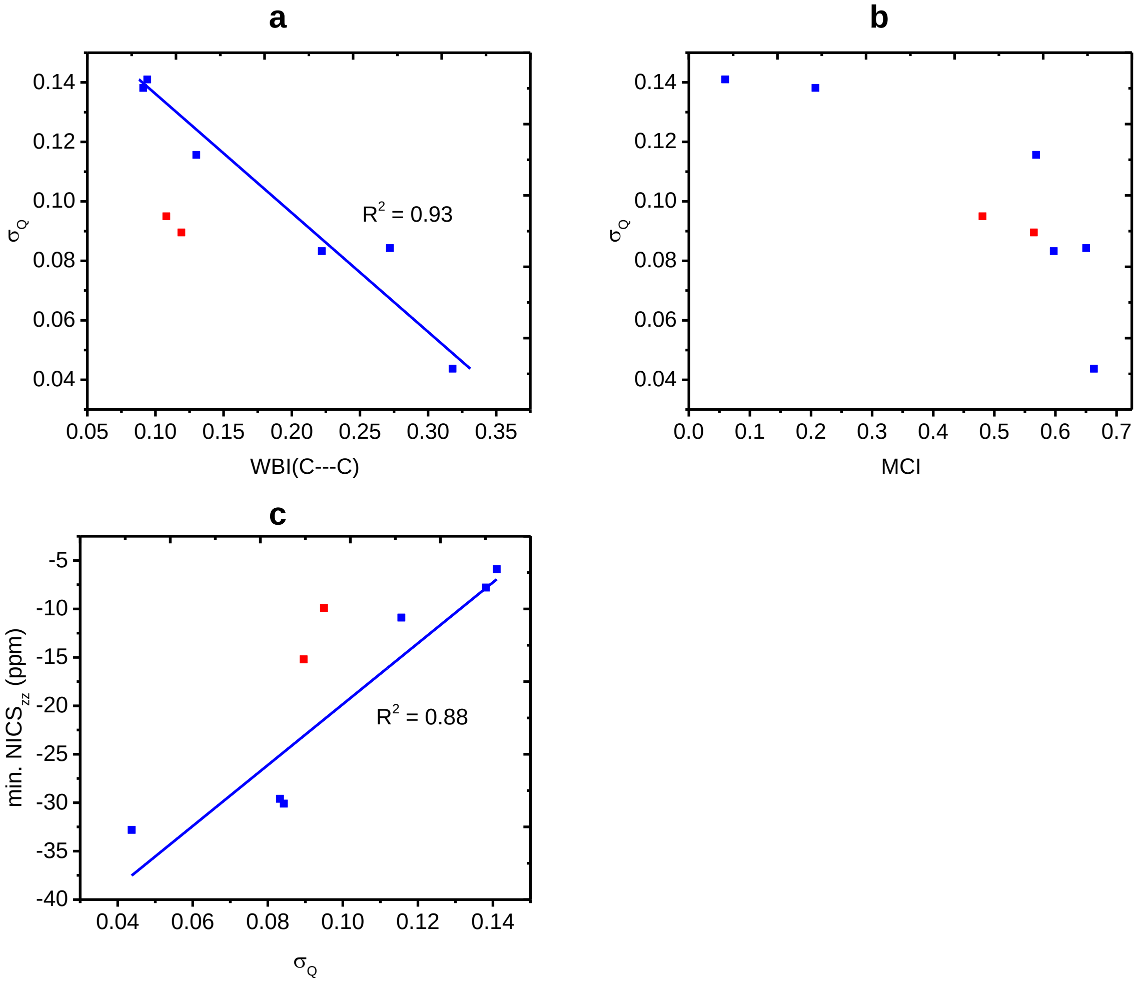

Figure S2. Correlation between σ_Q and (a) WBI(C---C), (b) MCI, and (c) minimum $NICS_{zz}$ values along the NICS scan for T_1-state candidate molecules.

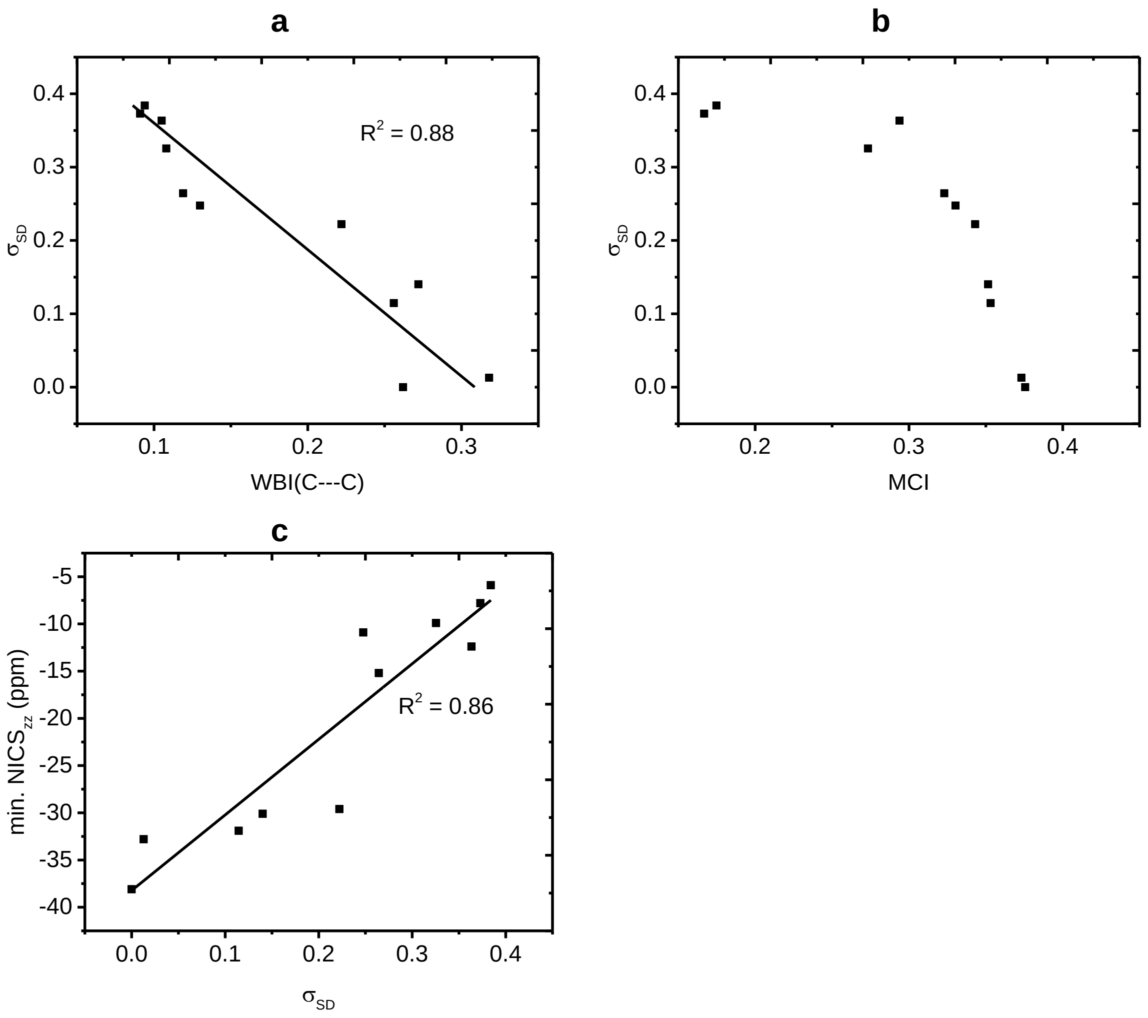

Figure S3. Correlation between σ_{SD} and (a) WBI(C---C) and (b) MCI, and (c) minimum $NICS_{zz}$ values along the NICS scan for T_1-state candidate molecules.

4 ISE energies

The methyl/methylene group can frequently be placed in different positions in the molecule. For the monohomoaromatics, the methyl group has consistently been placed in the 2-position (Figure S4a). For the bishomoaromatics, the placement of the methyl group is indicated in Figure S4b. For formation of the methylene unit, the hydrogen atom was placed on the carbon involved in the through-space conjugation. The ISE value for **17** in T_1 was calculated by B2PLYP single-point energies on the B3LYP geometries due to convergence issues in the B2PLYP geometry optimizations of one of the isomers.

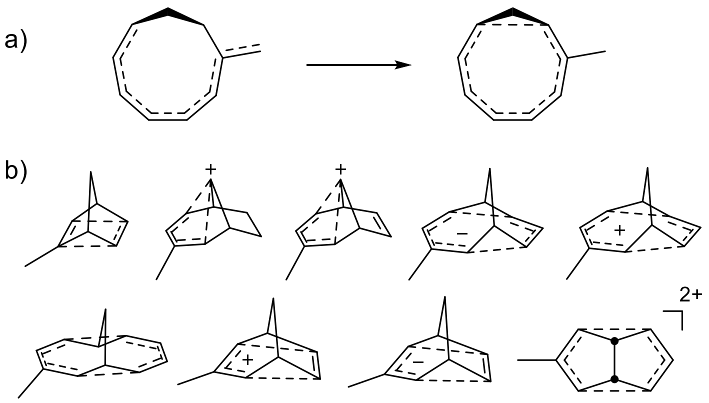

Figure S4. (a) Generic equation used to determine the isomerization stabilization energy showing the placement of the methyl group for the monocyclic compounds. (b) Placement of the methyl group in the bicyclic compounds.

5 Geometries

Figures of molecular geometries were generated using CYLview 1.0b[2]

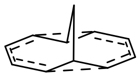

1a **T_1**

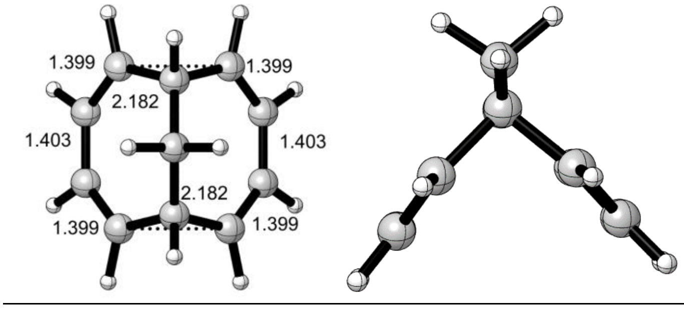

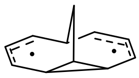

1b **T_1**

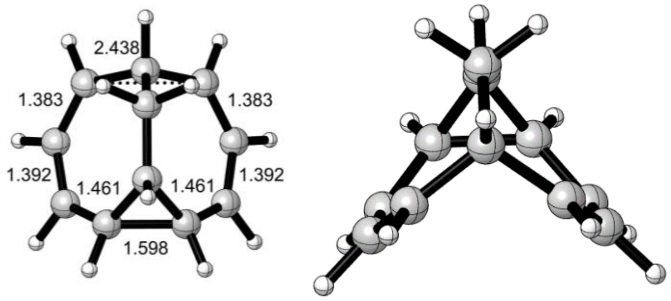

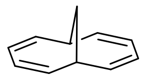

1c S_0

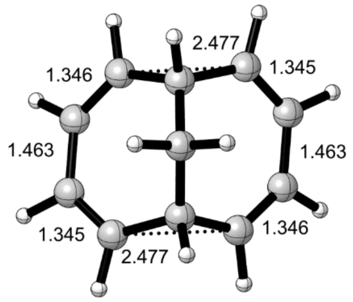

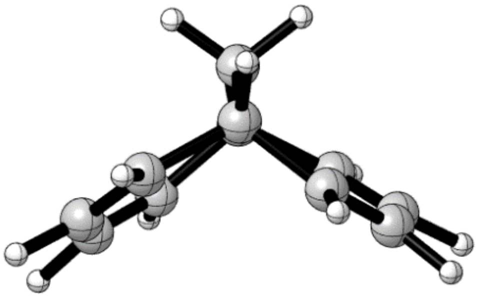

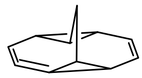

1d S_0

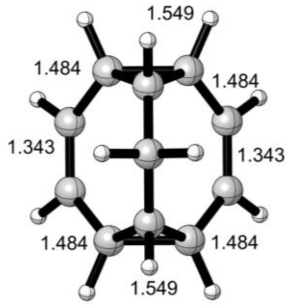

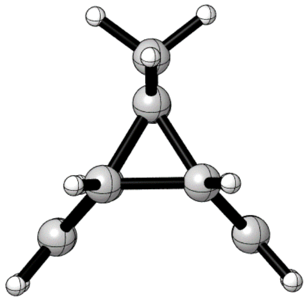

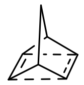

2a T_1

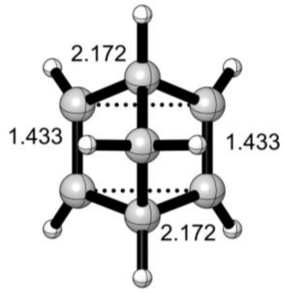

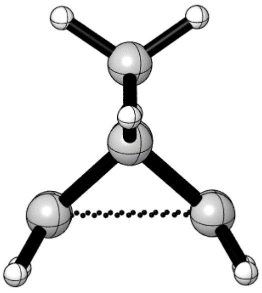

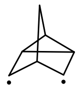

2b **T_1**

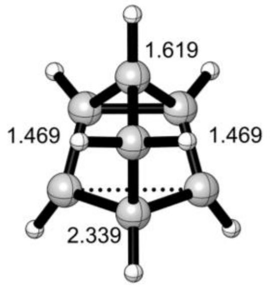

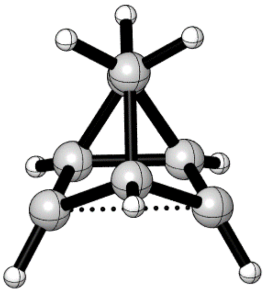

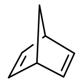

2c **S_0**

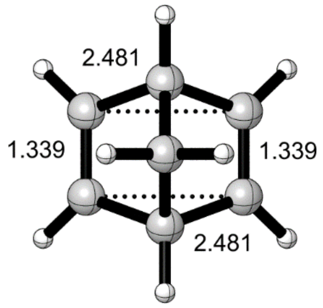

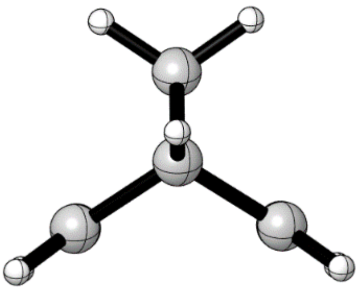

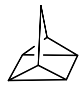

2d **S_0**

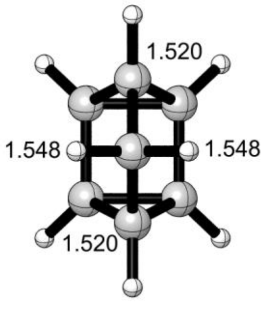

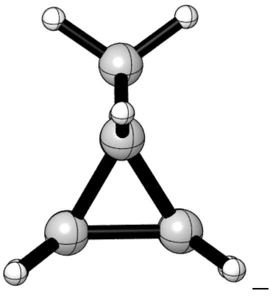

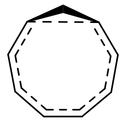

3a T_1

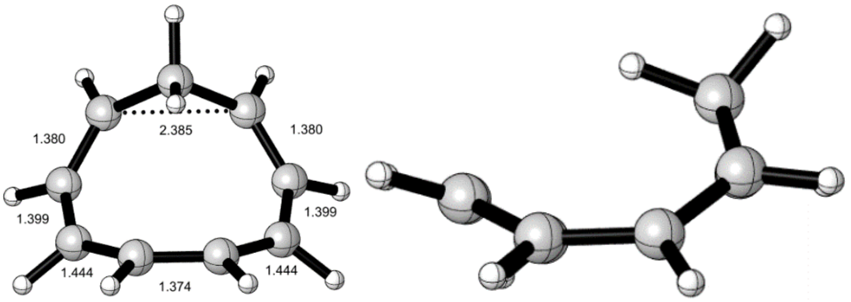

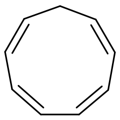

3b S_0

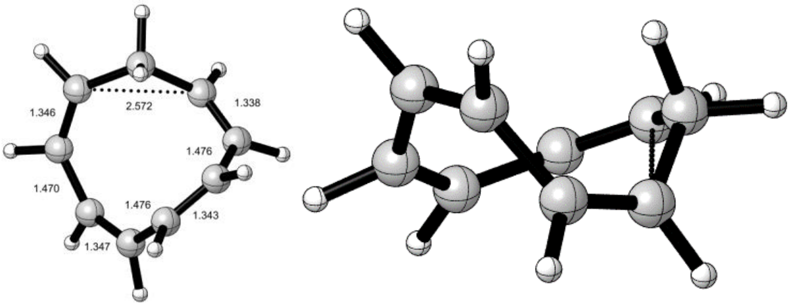

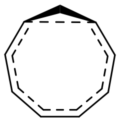

3c S_1

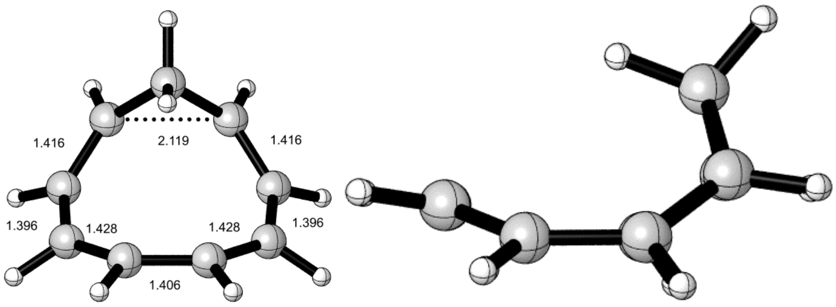

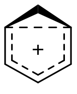

4a T_1

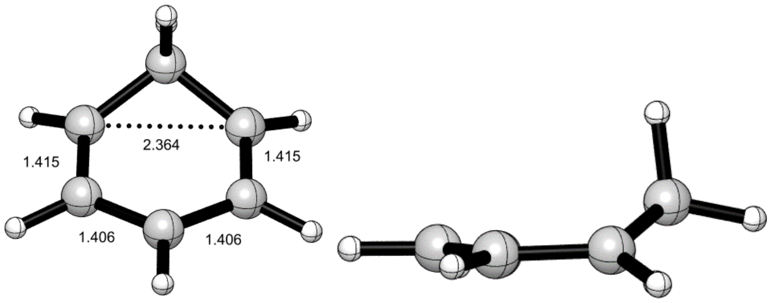

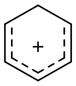

4b S_0

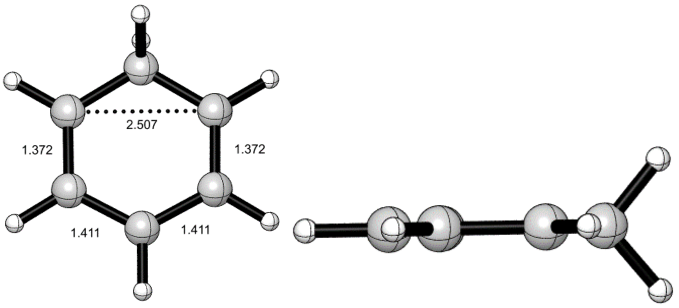

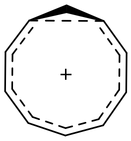

5a T_1

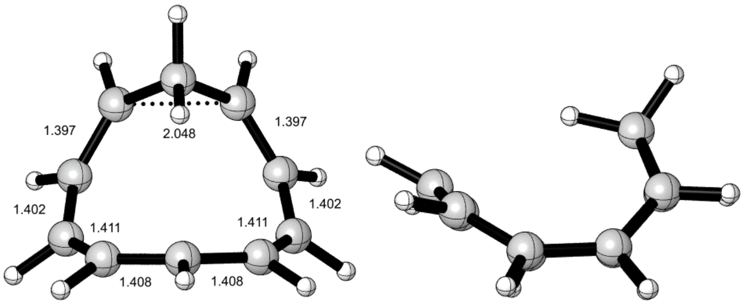

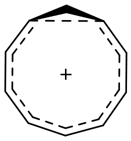

5b T_1

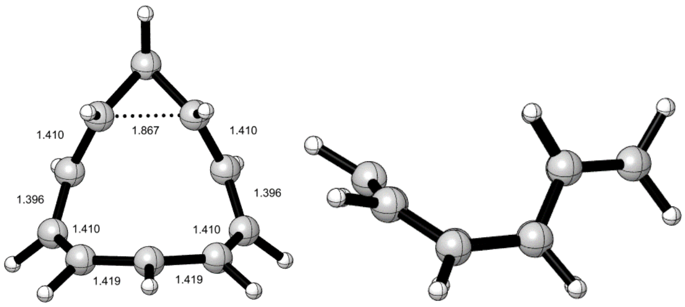
1.410
1.867
1.410
1.396
1.396
1.410
1.410
1.419
1.419

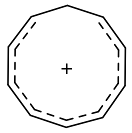
+

5c

S_0

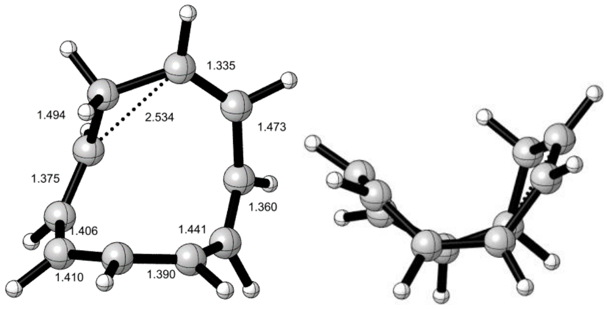
1.335
1.494
2.534
1.473
1.375
1.360
1.406
1.441
1.410
1.390

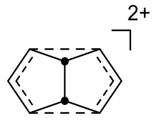
2+

6a

T_1

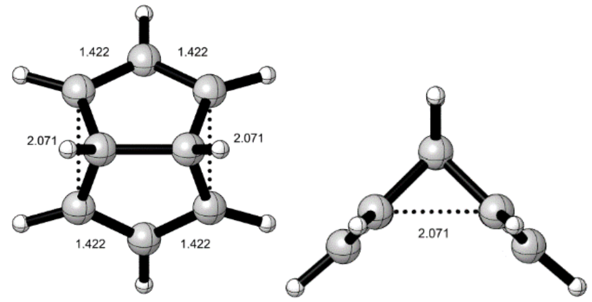
1.422
1.422
2.071
2.071
1.422
1.422
2.071

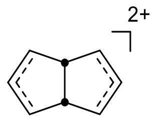

6b S_0

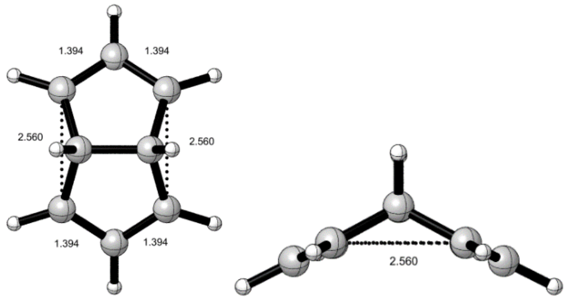

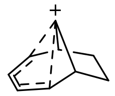

7a T_1

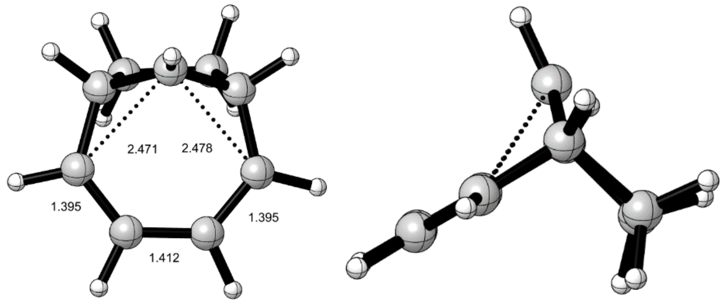

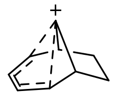

7b S_0

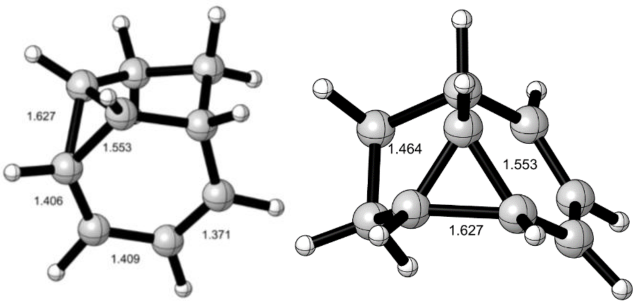

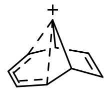

8a T_1

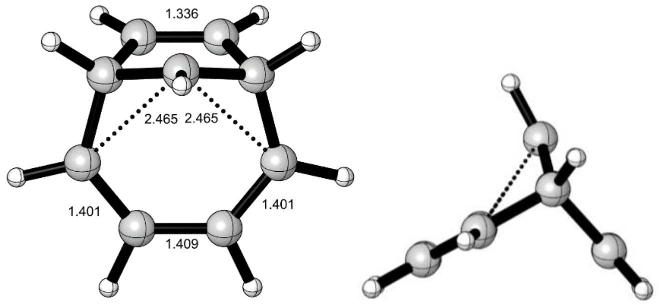

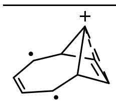

8b T_1

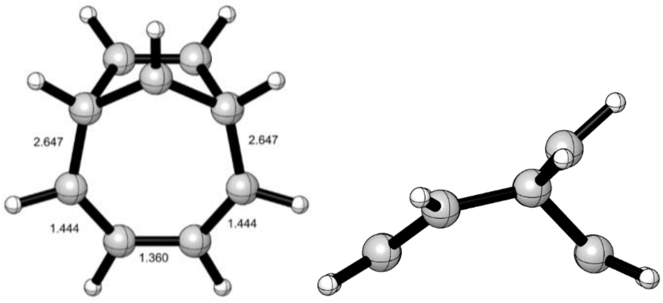

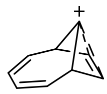

8c S_0

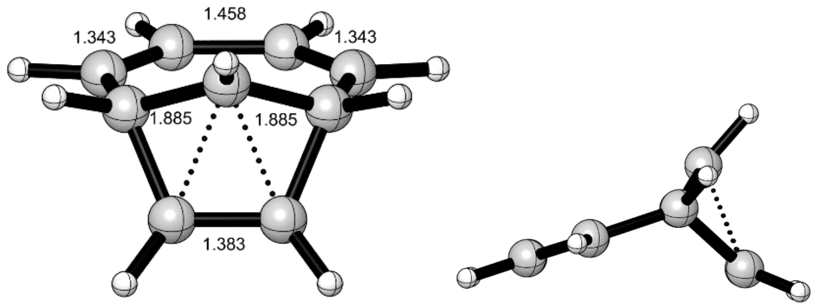

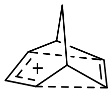

9a **T_1**

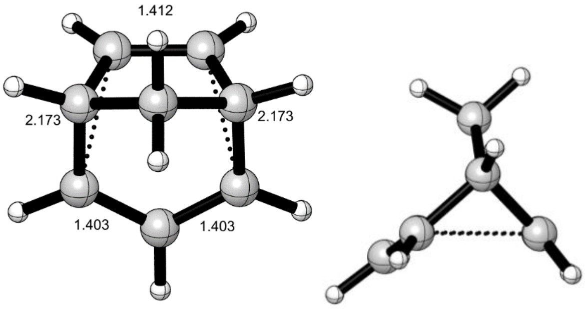

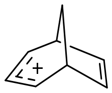

9b **S_0**

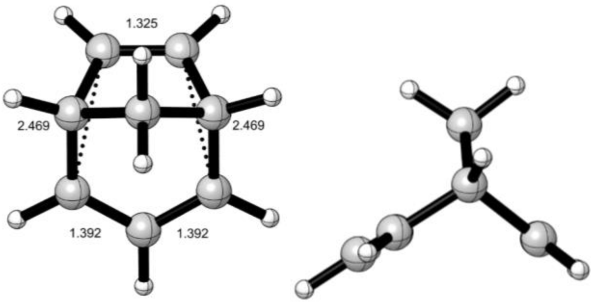

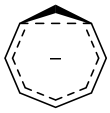

10a **T_1**

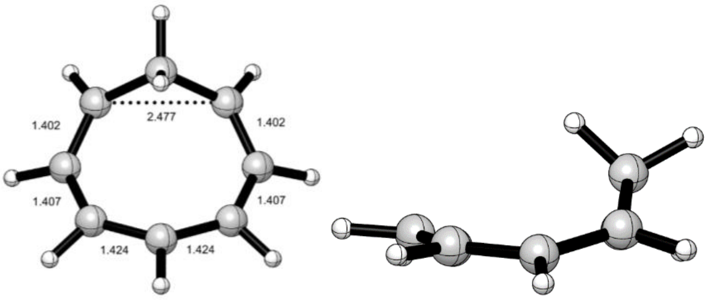

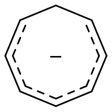

10b **S_0**

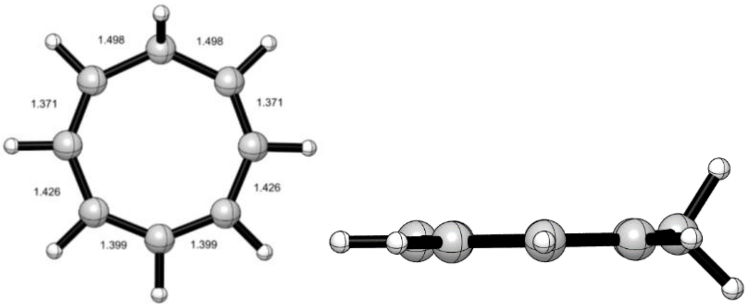

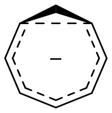

10c **S_1, C_{2v}, CASSCF**

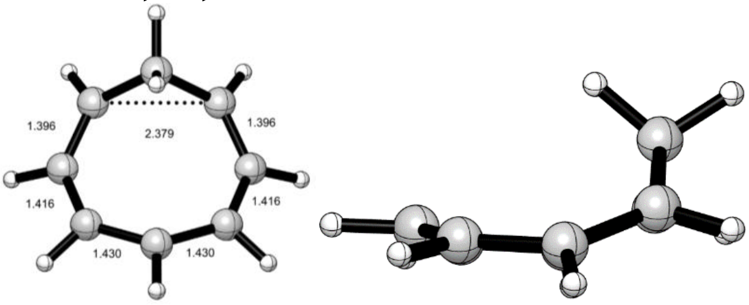

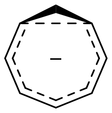

10c **S_1, C_{2v}, TD-B3LYP**

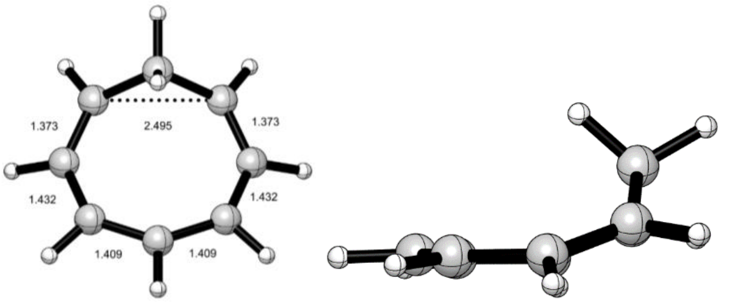

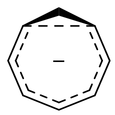

10d **S_1, C_1, CASSCF**

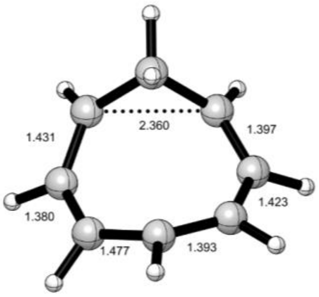

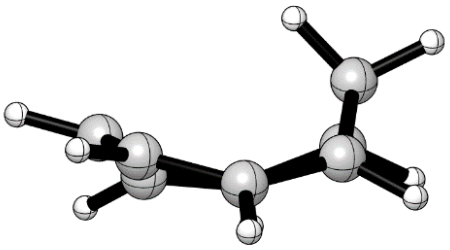

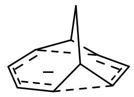

11a **T_1**

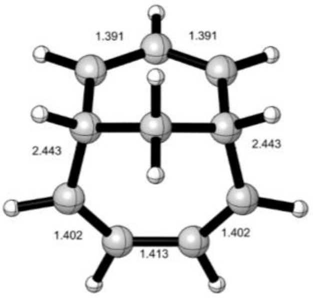

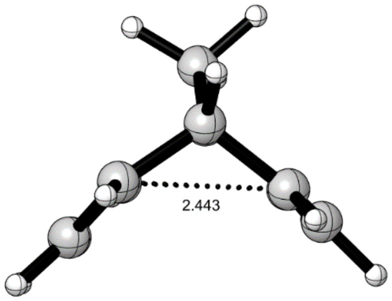

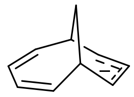

11b **S_0**

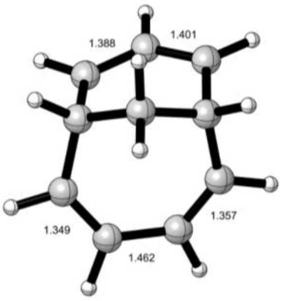

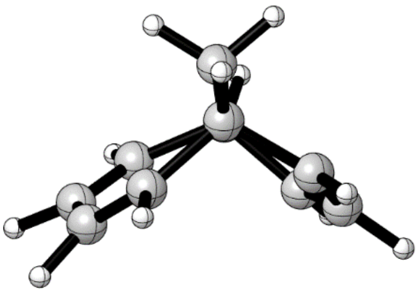

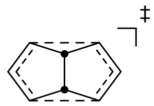

12 S_0

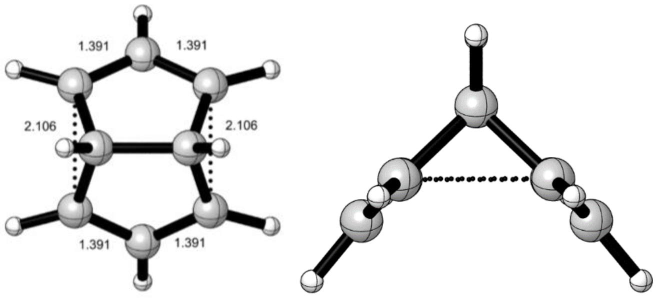

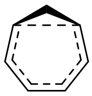

13 S_0

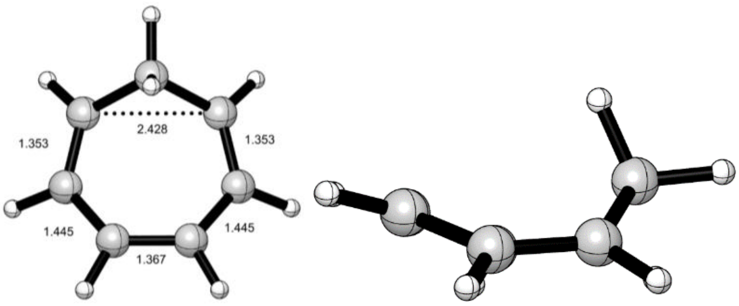

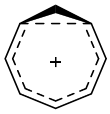

14 S_0

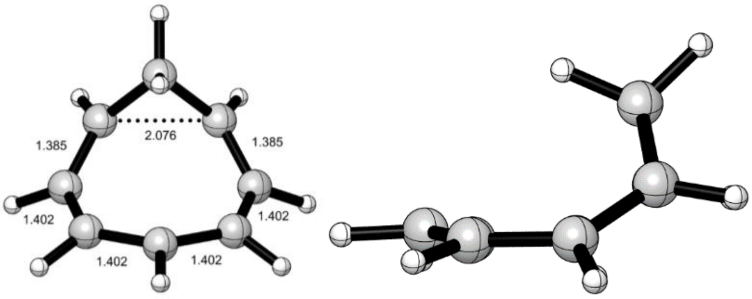

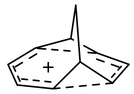

15 **S$_0$**

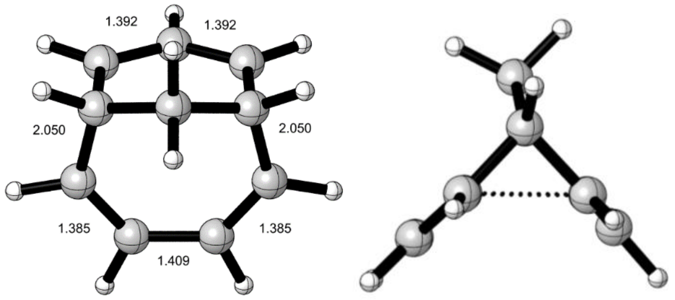

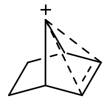

16 **S$_0$**

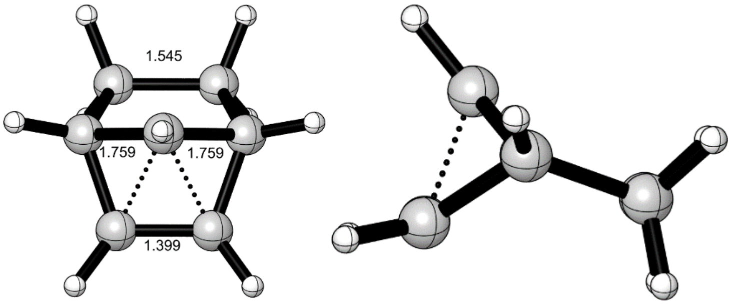

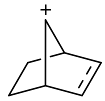

16 **T$_1$**

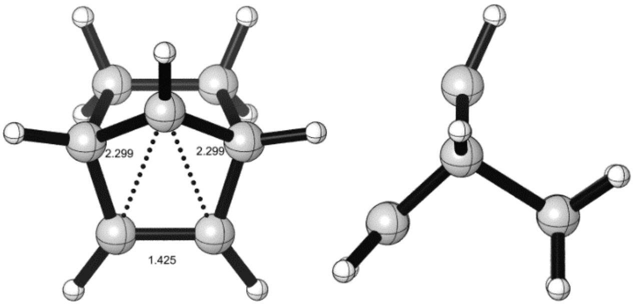

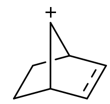

16 **S_0/S_1 conical intersection**

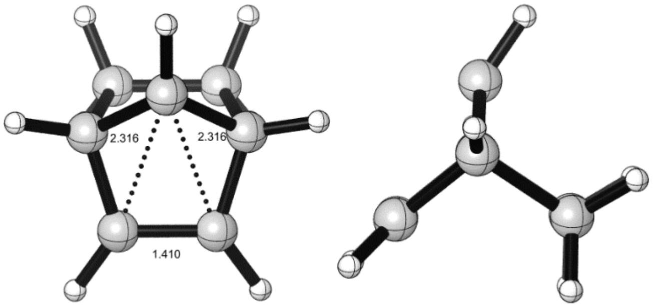

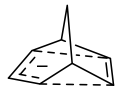

17 **S_0**

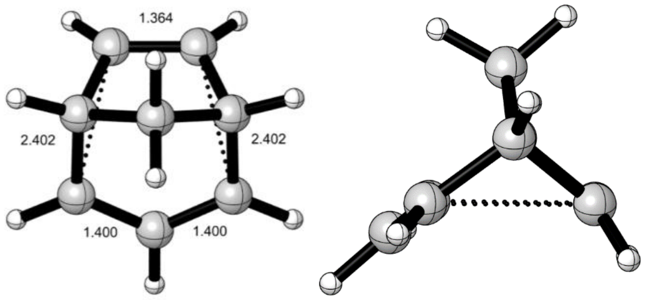

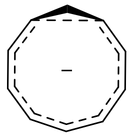

18 **S_0**

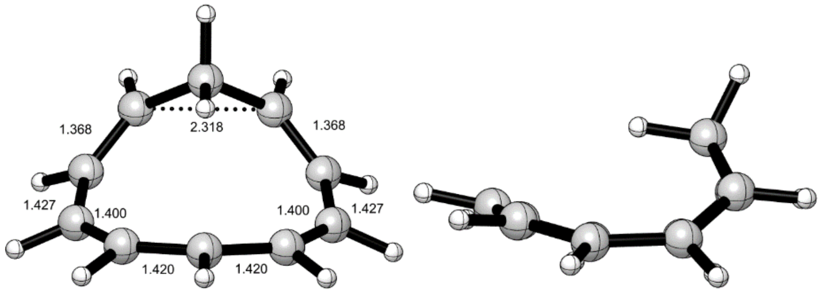

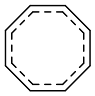

19 T$_1$

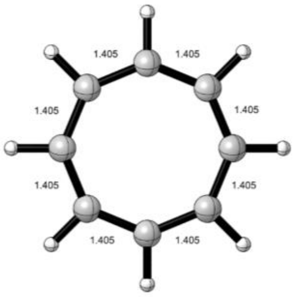

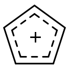

20 T$_1$

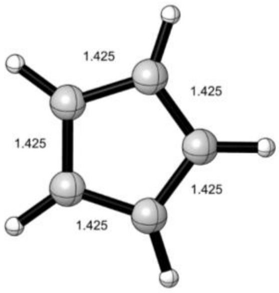

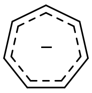

21 T$_1$

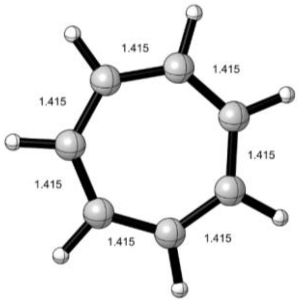

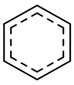

22 S$_0$

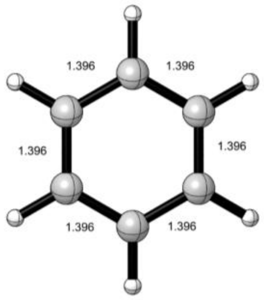

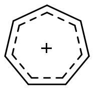

23 S_0

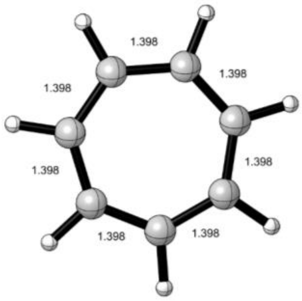

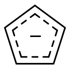

24 S_0

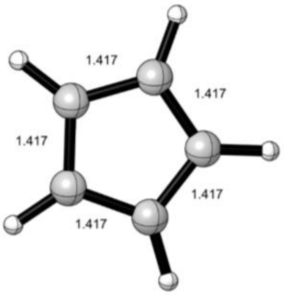

6 NICS scans

NICS scans were calculated with the B3LYP functional as GIAO calculations are not available for B2PLYP in Gaussian.

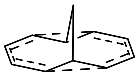

1 T_1

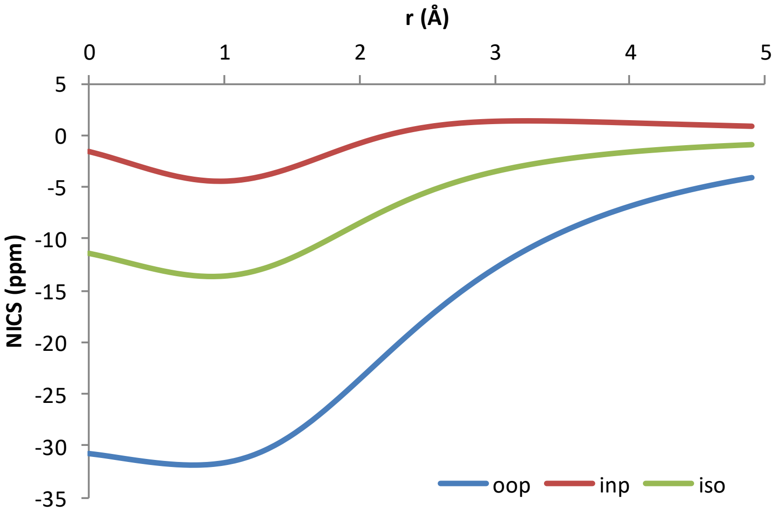

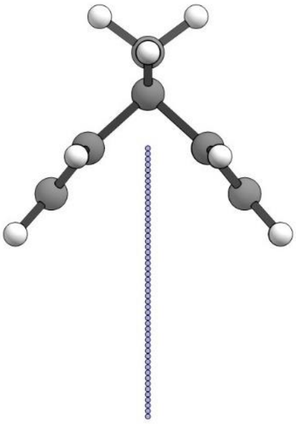

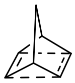

2 T$_1$

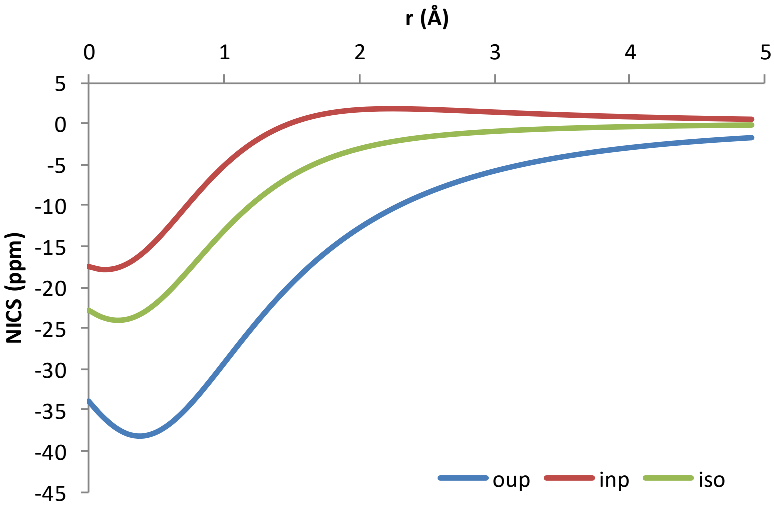
r (Å)
0
1
2
3
4
5
5
0
-5
-10
-15
-20
-25
-30
-35
-40
-45
NICS (ppm)
oup
inp
iso

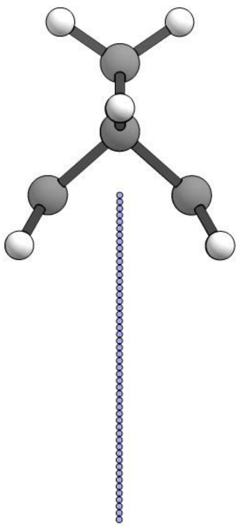

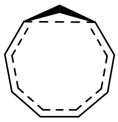

3 T$_1$

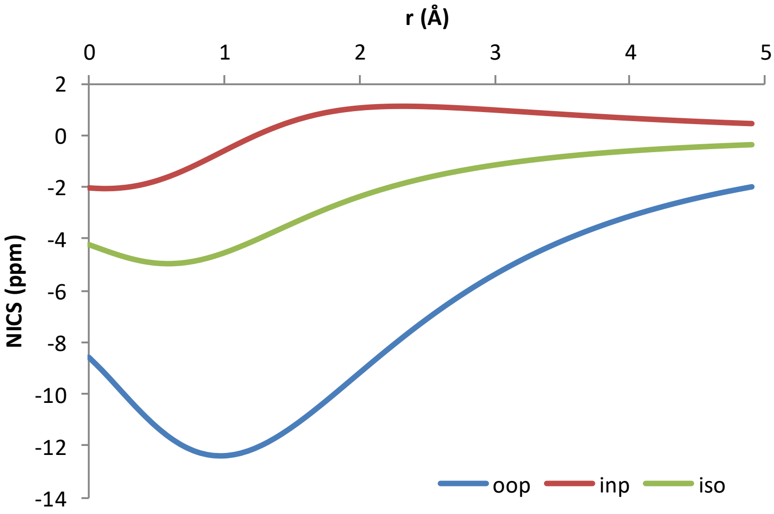
r (Å)
0
1
2
3
4
5
2
0
-2
-4
-6
-8
-10
-12
-14
NICS (ppm)
oop
inp
iso

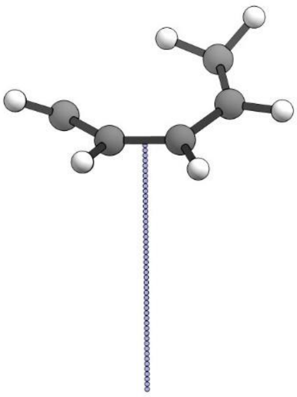

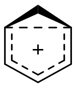

4 T_1

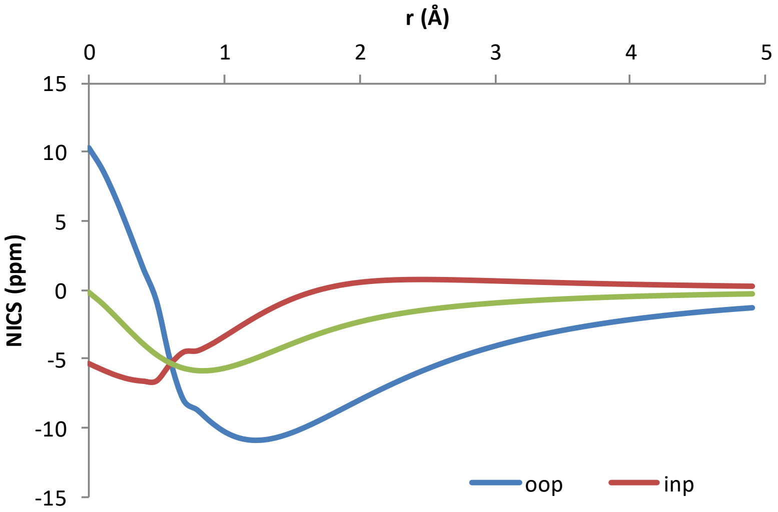
r (Å)
0
1
2
3
4
5
15
10
5
0
-5
-10
-15
NICS (ppm)
oop
inp

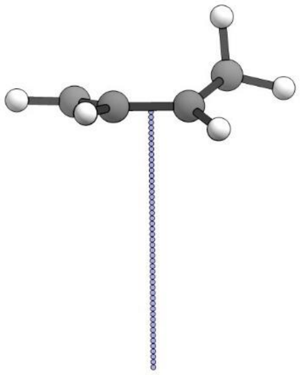

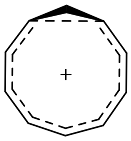

5a T_1

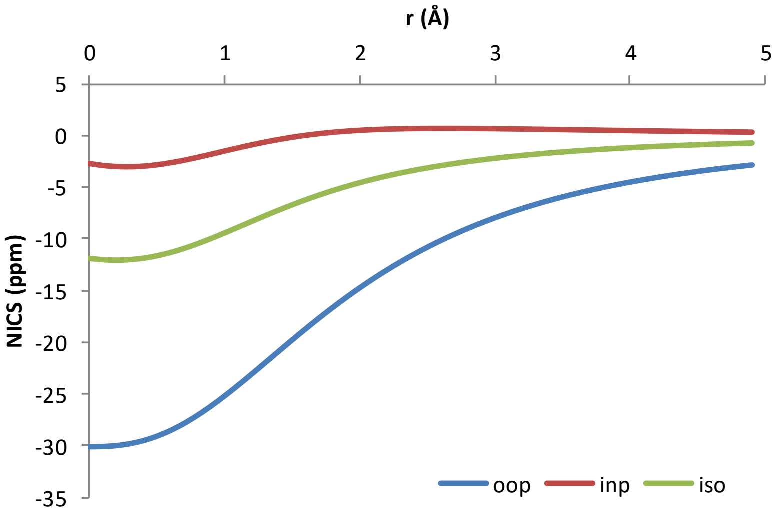
r (Å)
0
1
2
3
4
5
5
0
-5
-10
-15
-20
-25
-30
-35
NICS (ppm)
oop
inp
iso

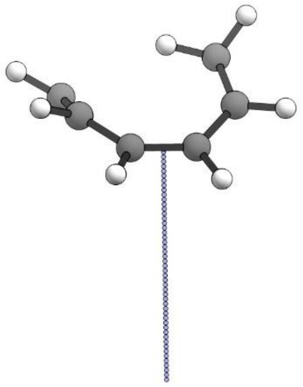

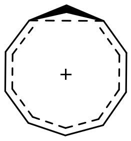

5b $\mathbf{T_1}$

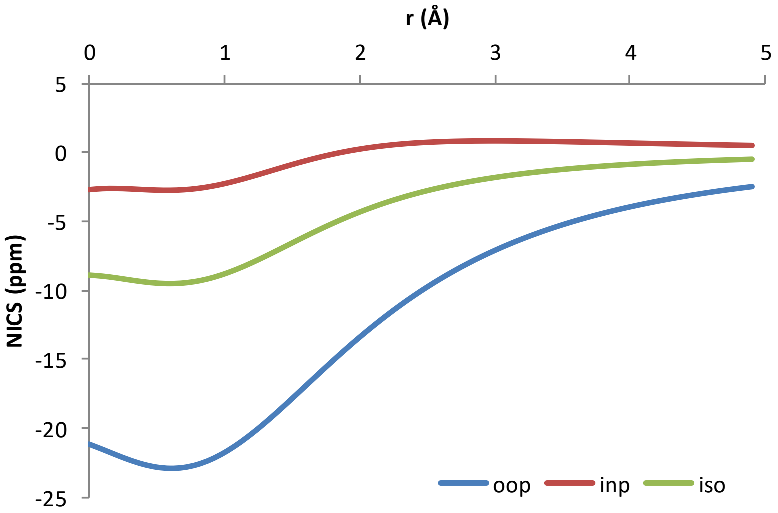
r (Å)
0
1
2
3
4
5
5
0
-5
-10
-15
-20
-25
NICS (ppm)
oop
inp
iso

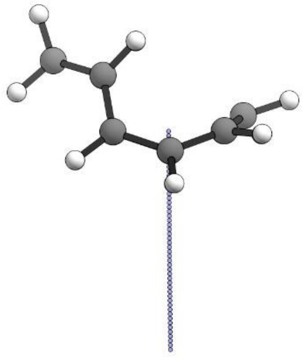

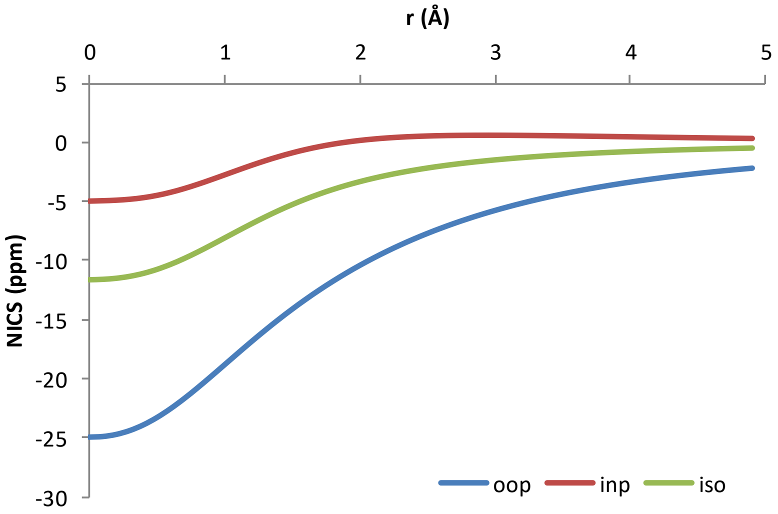
r (Å)
0
1
2
3
4
5
5
0
-5
-10
-15
-20
-25
-30
NICS (ppm)
oop
inp
iso

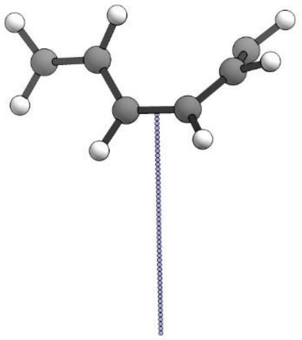

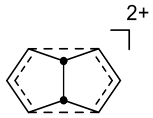
2+

6 T_1

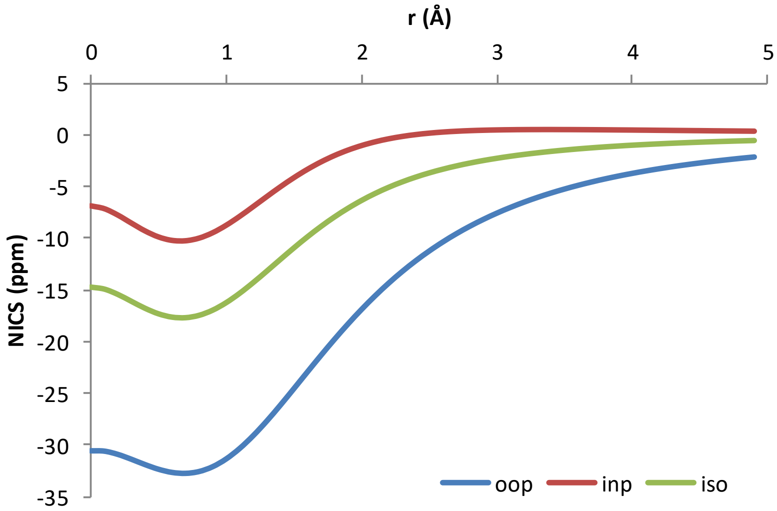
r (Å)
0
1
2
3
4
5
NICS (ppm)
5
0
-5
-10
-15
-20
-25
-30
-35
oop
inp
iso

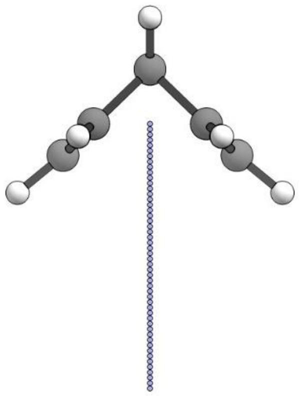

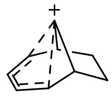
+

7 T_1

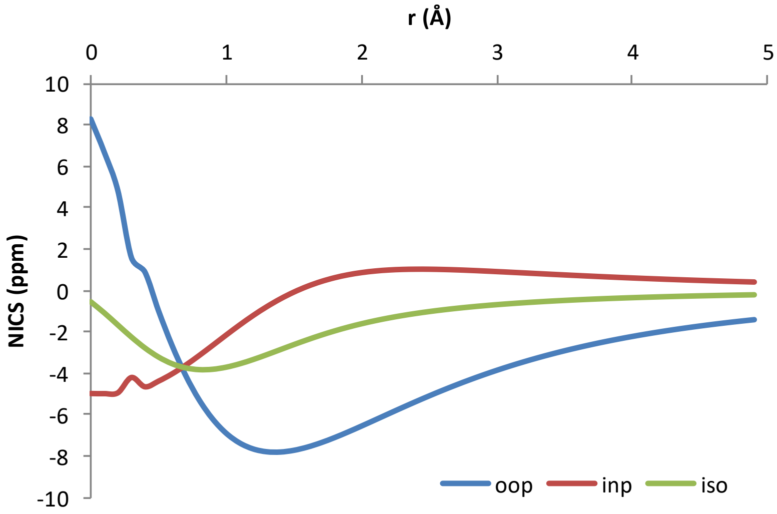
r (Å)
0
1
2
3
4
5
NICS (ppm)
10
8
6
4
2
0
-2
-4
-6
-8
-10
oop
inp
iso

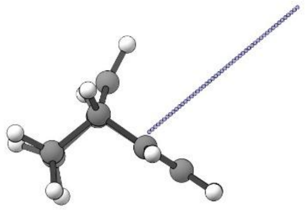

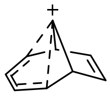

8 **T_1**

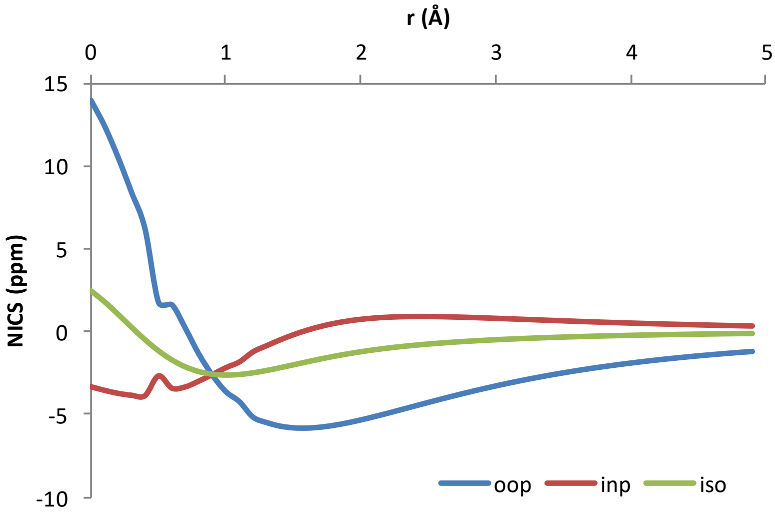
r (Å)
0
1
2
3
4
5
15
10
5
0
-5
-10
NICS (ppm)
oop
inp
iso

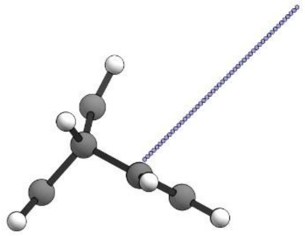

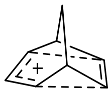

9 **T_1**

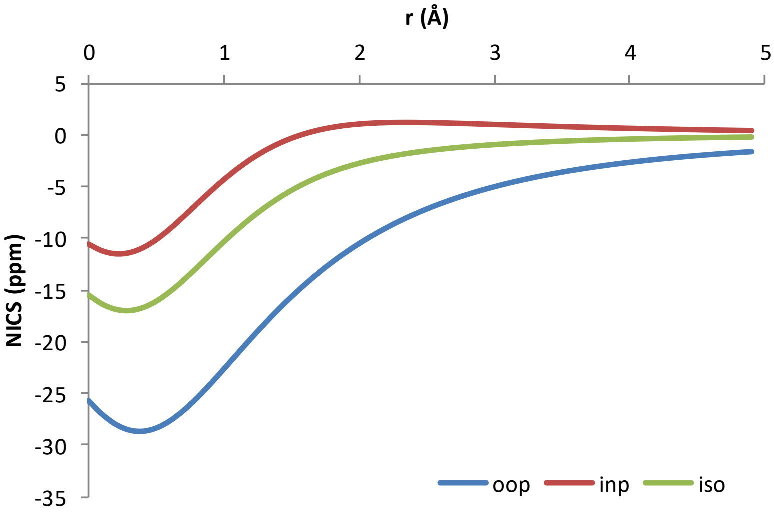
r (Å)
0
1
2
3
4
5
5
0
-5
-10
-15
-20
-25
-30
-35
NICS (ppm)
oop
inp
iso

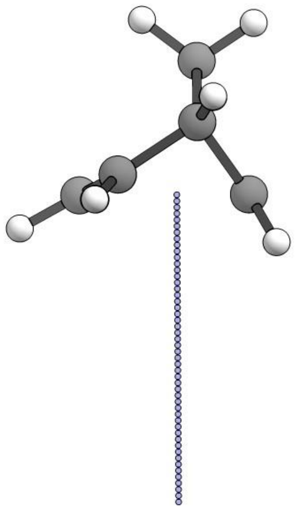

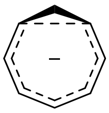

10 T_1

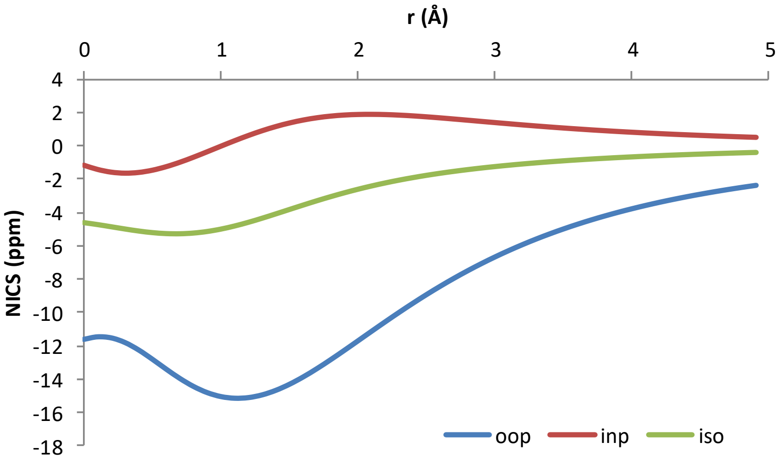

r (Å)
0
1
2
3
4
5
4
2
0
-2
-4
-6
-8
-10
-12
-14
-16
-18
NICS (ppm)
oop
inp
iso

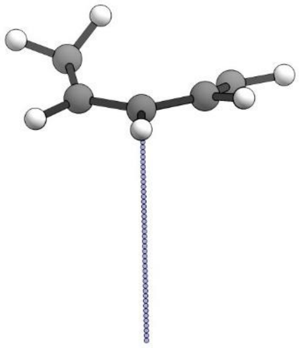

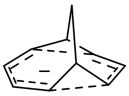

11 T_1

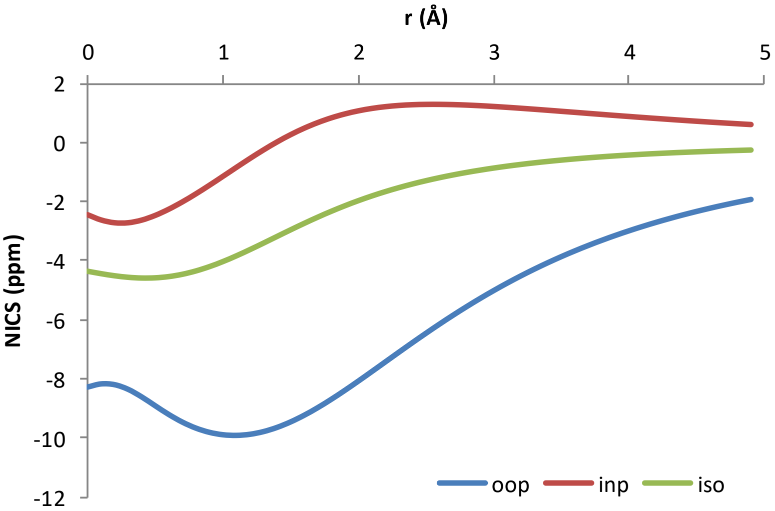

r (Å)
0
1
2
3
4
5
2
0
-2
-4
-6
-8
-10
-12
NICS (ppm)
oop
inp
iso

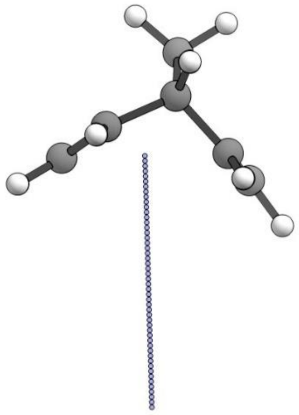

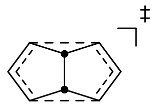

12 S_0

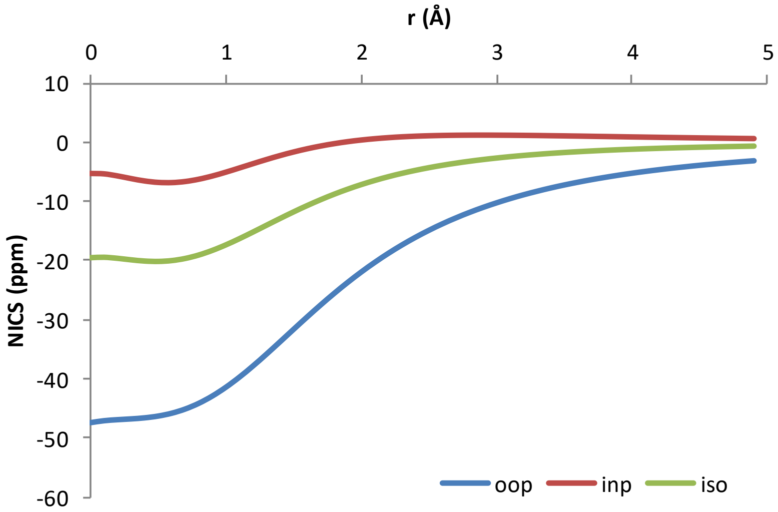

r (Å)
0
1
2
3
4
5
10
0
-10
-20
-30
-40
-50
-60
NICS (ppm)
oop
inp
iso

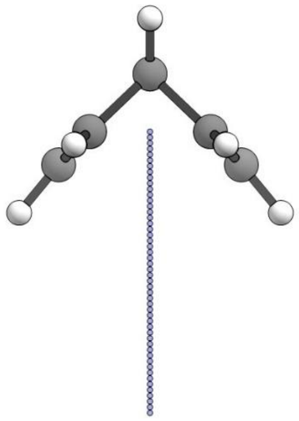

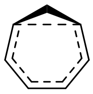

13 S_0

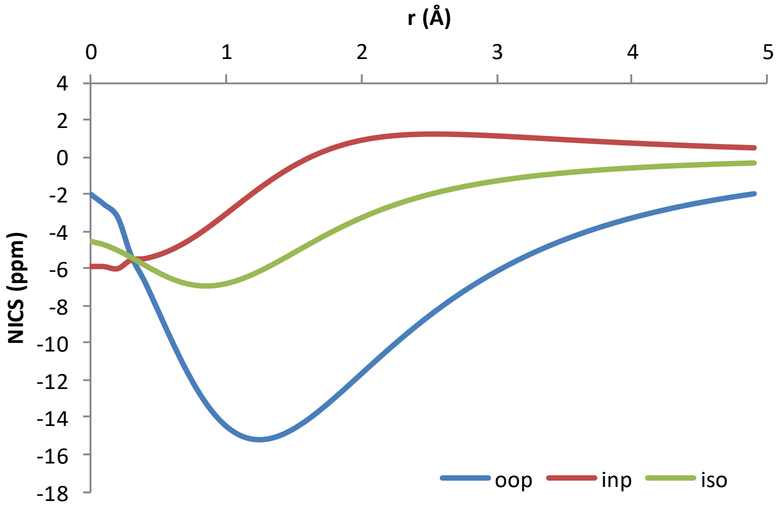

r (Å)
0
1
2
3
4
5
4
2
0
-2
-4
-6
-8
-10
-12
-14
-16
-18
NICS (ppm)
oop
inp
iso

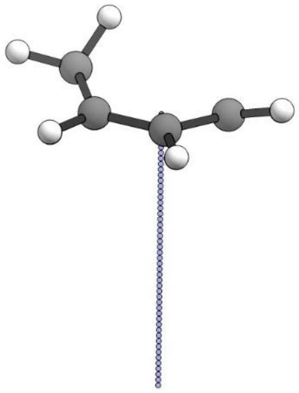

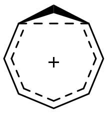

14 S$_0$

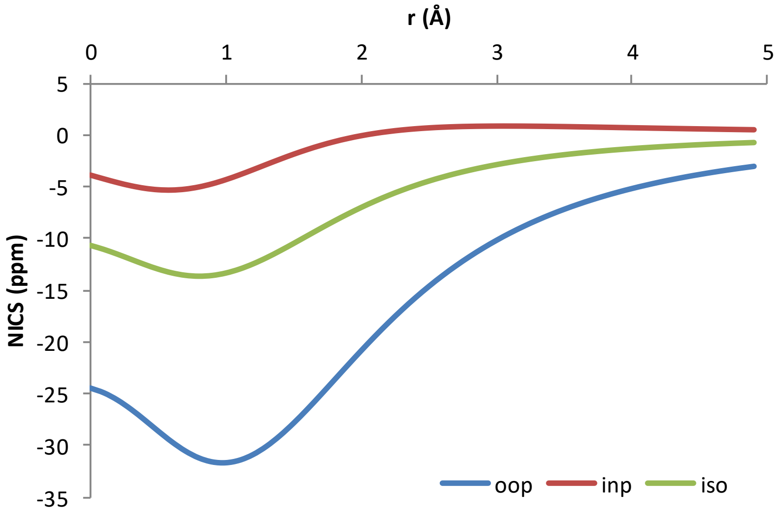
r (Å)
0
1
2
3
4
5
5
0
-5
-10
-15
-20
-25
-30
-35
NICS (ppm)
oop
inp
iso

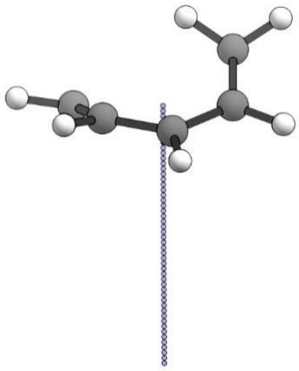

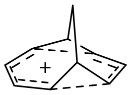
+

15 S$_0$

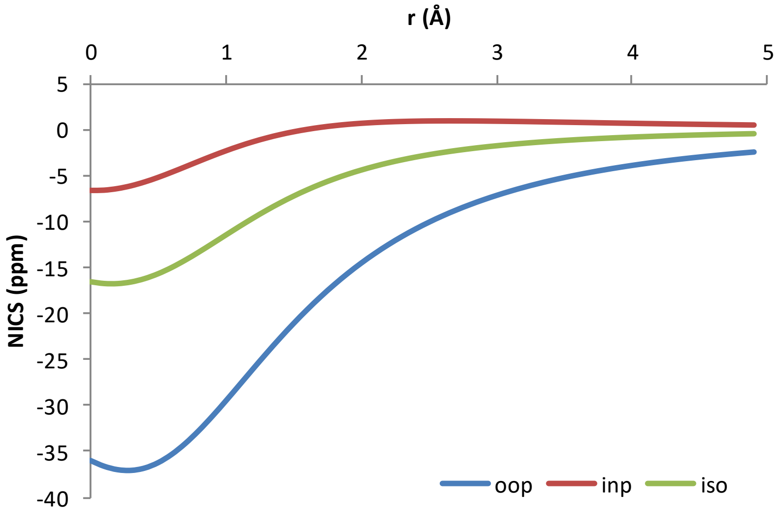
r (Å)
0
1
2
3
4
5
5
0
-5
-10
-15
-20
-25
-30
-35
-40
NICS (ppm)
oop
inp
iso

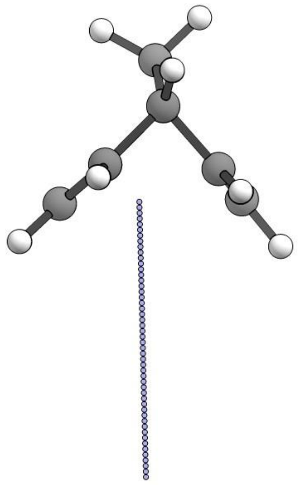

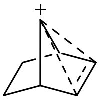

16 S_0

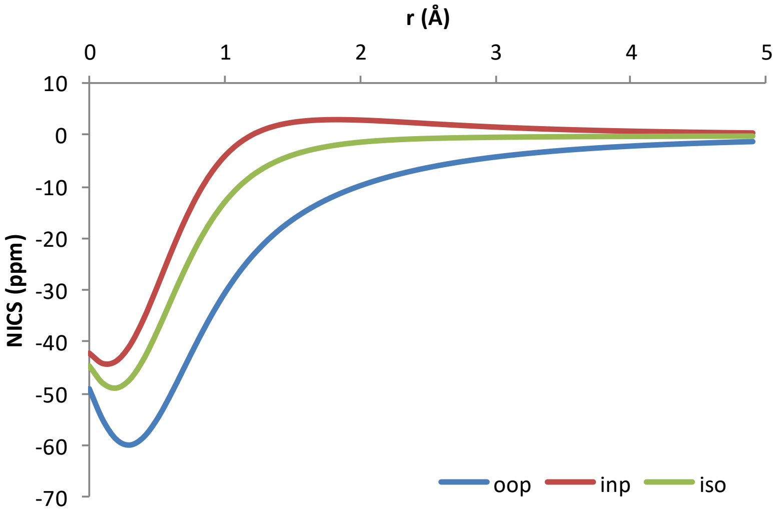
r (Å)
0
1
2
3
4
5
10
0
-10
-20
-30
-40
-50
-60
-70
NICS (ppm)
oop
inp
iso

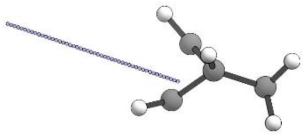

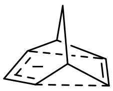

17 S_0

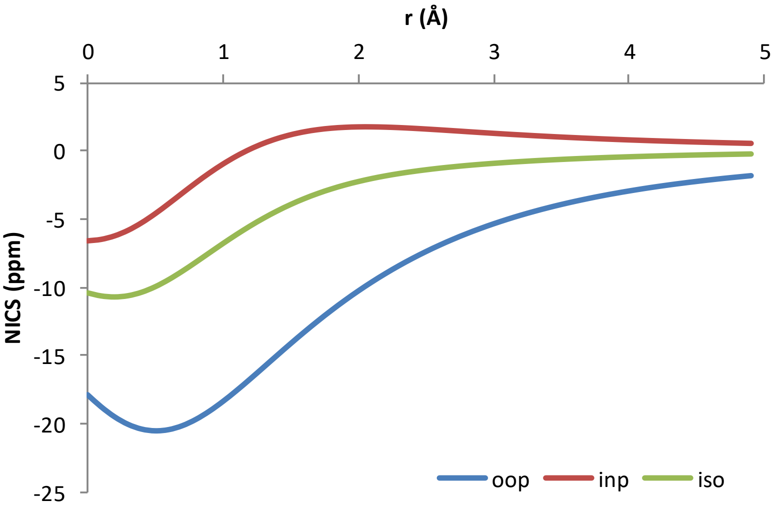
r (Å)
0
1
2
3
4
5
5
0
-5
-10
-15
-20
-25
NICS (ppm)
oop
inp
iso

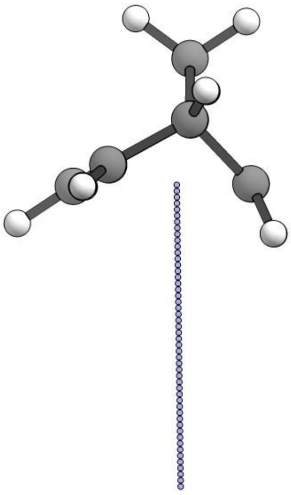

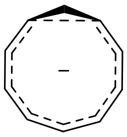

18 S_0

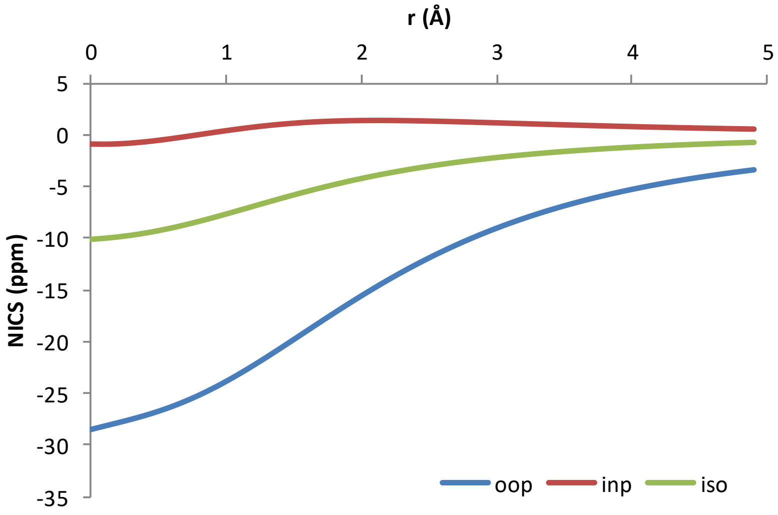

r (Å)
0
1
2
3
4
5
5
0
-5
-10
-15
-20
-25
-30
-35
NICS (ppm)
oop
inp
iso

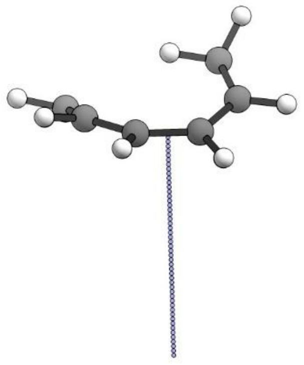

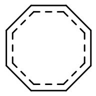

19 T_1

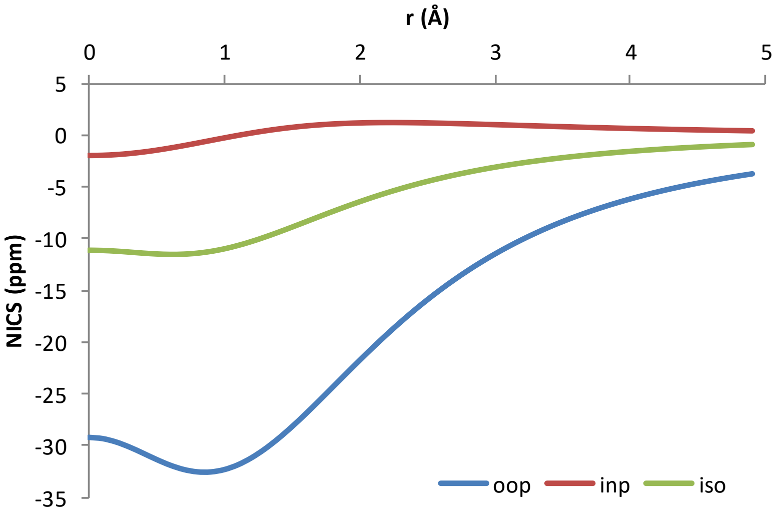

r (Å)
0
1
2
3
4
5
5
0
-5
-10
-15
-20
-25
-30
-35
NICS (ppm)
oop
inp
iso

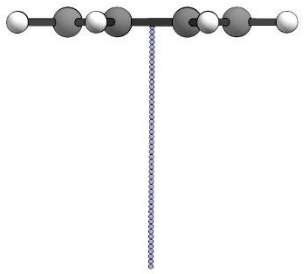

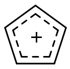
+

20 T_1

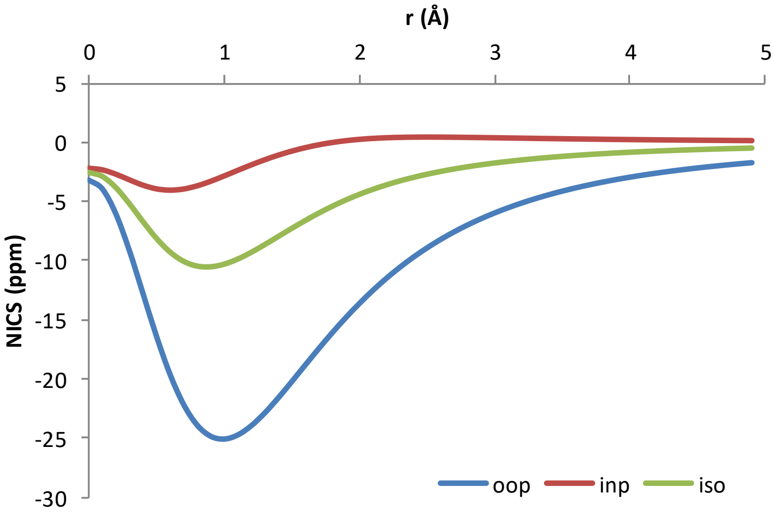
r (Å)
0
1
2
3
4
5
5
0
-5
-10
-15
-20
-25
-30
NICS (ppm)
oop
inp
iso

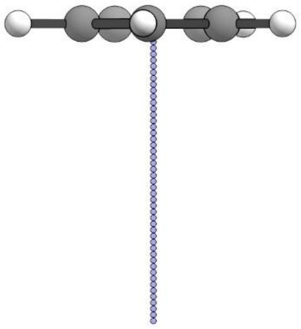

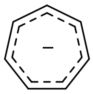
−

21 T_1

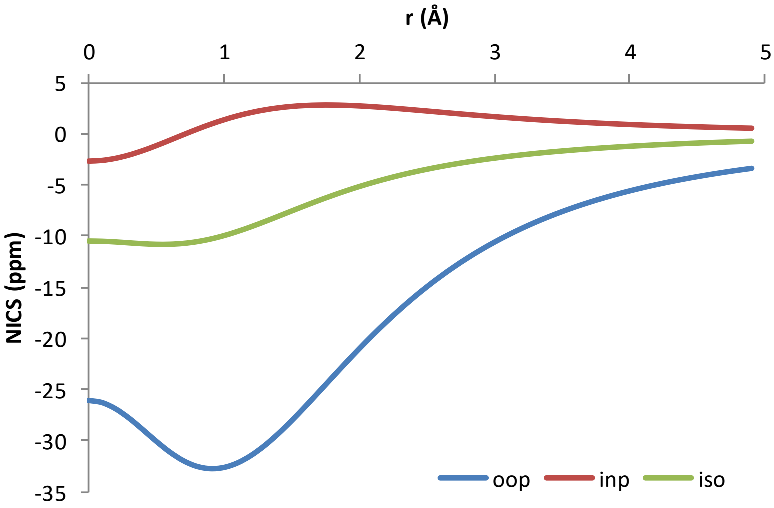
r (Å)
0
1
2
3
4
5
5
0
-5
-10
-15
-20
-25
-30
-35
NICS (ppm)
oop
inp
iso

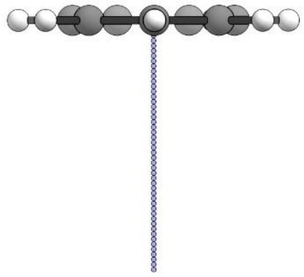

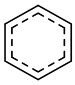

22 S_0

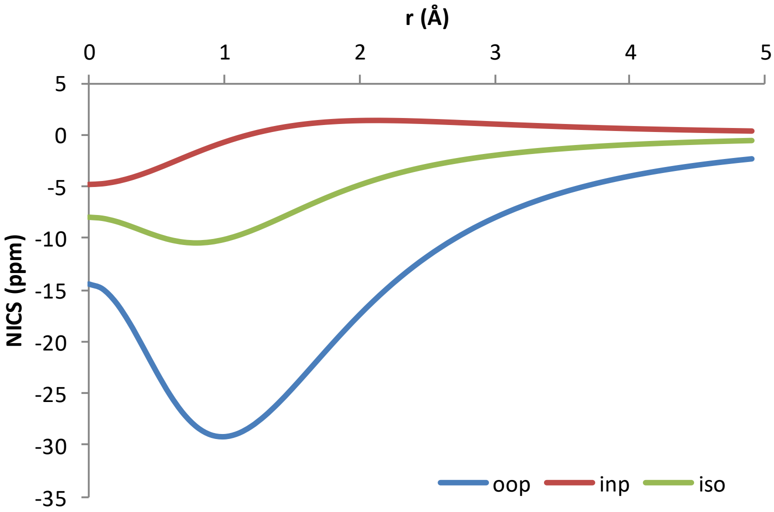
r (Å)
0
1
2
3
4
5
5
0
-5
-10
-15
-20
-25
-30
-35
NICS (ppm)
oop
inp
iso

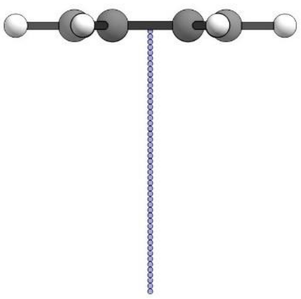

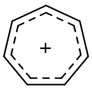
+

23 S_0

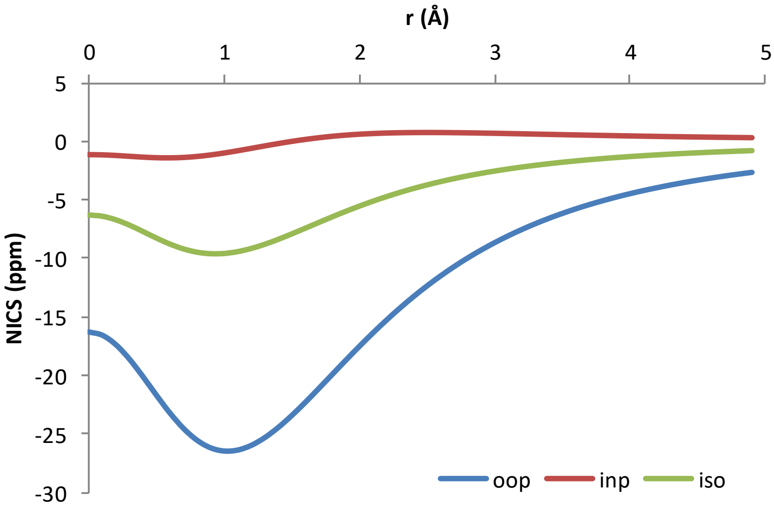
r (Å)
0
1
2
3
4
5
5
0
-5
-10
-15
-20
-25
-30
NICS (ppm)
oop
inp
iso

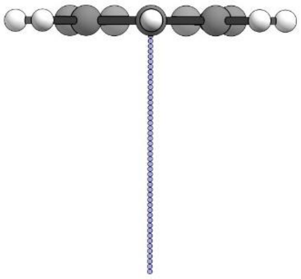

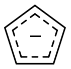

24 **S_0**

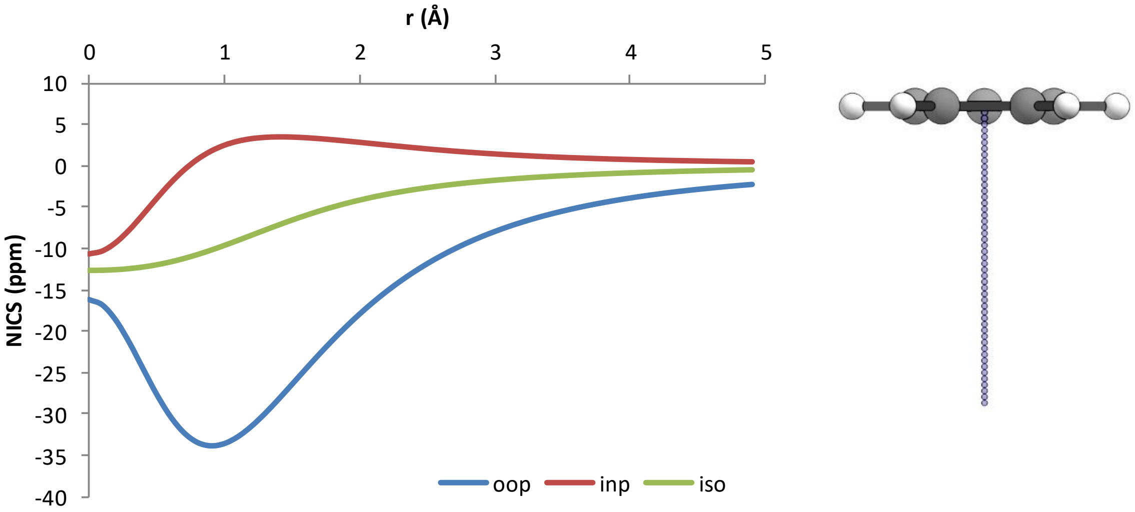

7 S_1 and T_1 calculations of compound 16

The minimum energy path (MEP) was calculated in the S_1 state with 3-state-average CASSCF/6-31G(d) including the three lowest states of singlet multiplicity. Single-point CASSCF and CASPT2 energies where then calculated on this path also with state-specific approach for the T_1 state and with multistate CASPT2 for the S_0, S_1 and S_2 states. The plot with the CASSCF energies shows a smooth relaxation in S1 towards a S_0/S_1 conical intersection (CI, Figure S5). The T_1 state is overstabilized at the CASSCF level and is likely isoenergetic with S_0 and S_1. This is more evident at the MS-CASPT2 level, where T_1 is lifted above S_0 (Figure S6). However, the location of the CI is slightly shifted compared to CASSCF so the S_0/S_1 degeneracy is not seen at this level (would require separate optimization). NICS scans for the vertical and relaxed T_1 geometries are given in Figure S7 and Figure S8.

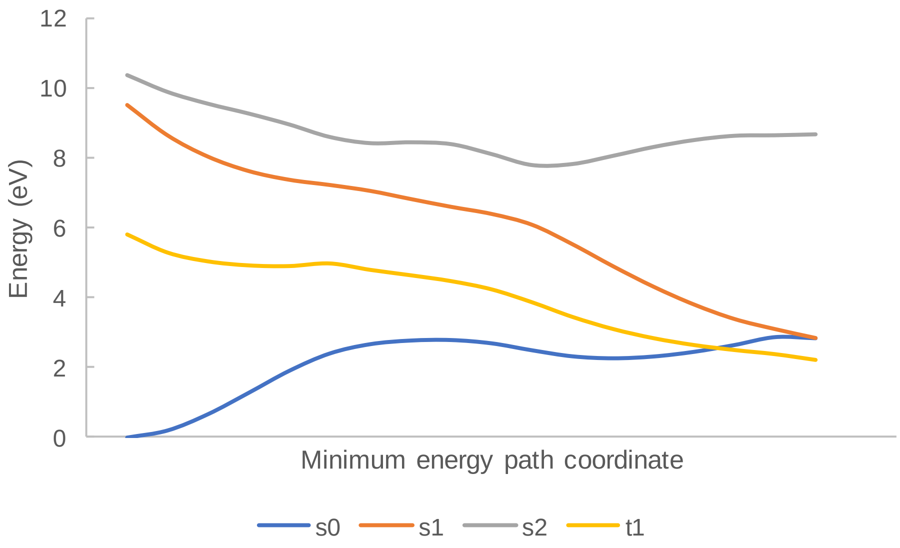

Figure S5. CASSCF energies on minimum energy path in S_1 following vertical excitation for **16**.

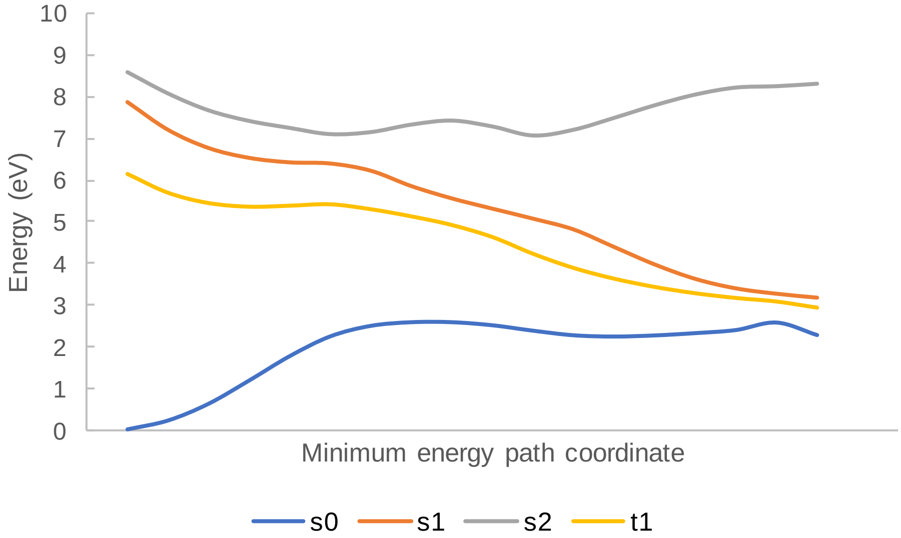

Figure S6. MS-CASPT2 energies on minimum energy path in S_1 following vertical excitation for **16**.

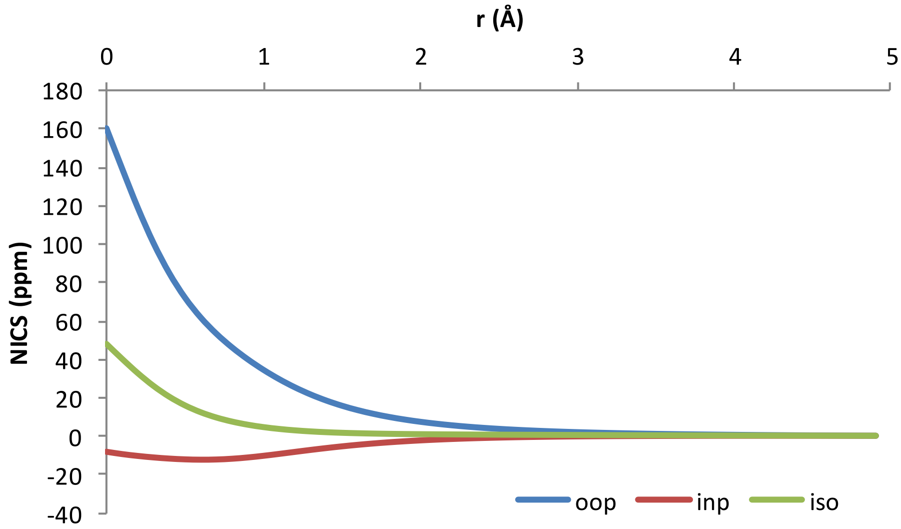

Figure S7. T_1 NICS scan at S_0 geometry for **16**. GIAO-B3LYP/6-311+G(d,p)

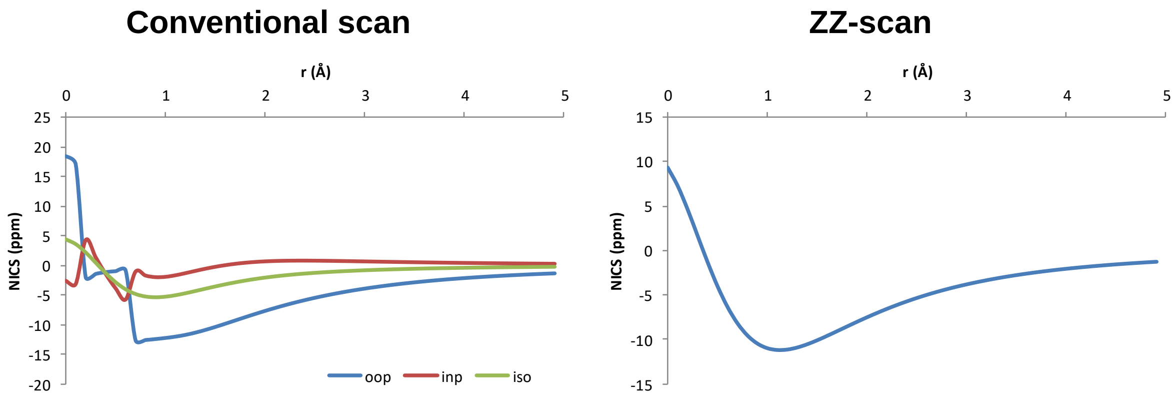

Figure S8. T_1 NICS scan at T_1 geometry for **16**. GIAO-B3LYP/6-311+G(d,p)

8 S_1 NICS scans

NICS scans were calculated with state-specific CASSCF/6-31++G(d,p) using Dalton 2016.0. These should be seen as giving qualitative rather than quantitative indications of aromaticity as NICS has not yet been extensively evaluated for the singlet excited states. The shape of the NICS curve (maximum/minimum) should be regarded rather than the absolute values. Here we have given scans of $NICS_{iso}$ values due to computational convenience. As shown previously, $NICS_{iso}$ shows the same trends as the out-of-plane component for determining aromaticity.[3] The plot for the optimized structure of **3** in the S_1 state shows the minimum in the NICS which is characteristic for aromatic compounds (Figure S9). For **10**, both the symmetric conformation which is favoured by TD-DFT and the skewed conformation which is favoured by CASSCF give aromatic-type NICS scans (Figure S10).

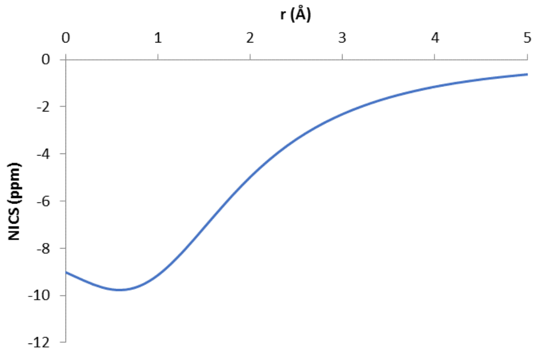

Figure S9. $NICS_{iso}$ scan of **3** in the S_1 state.

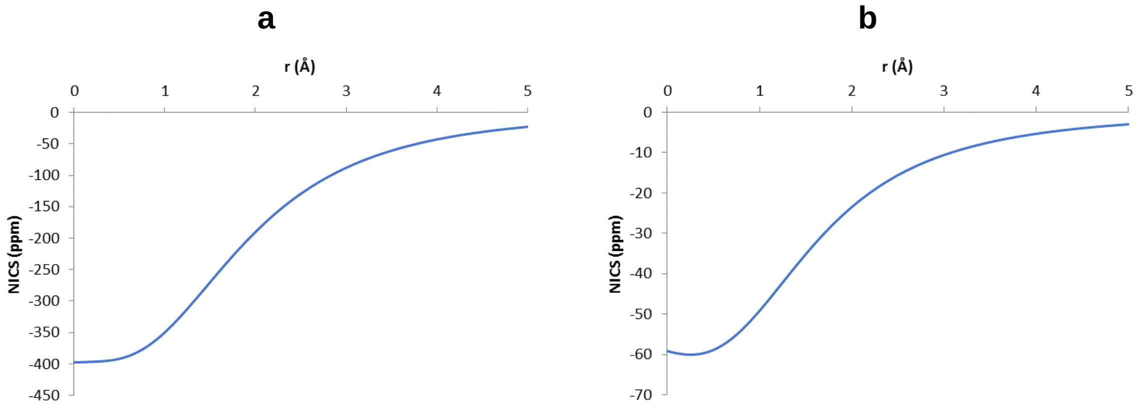

Figure S10. $NICS_{iso}$ scan of **10** in the (a) the symmetric S_1 conformation and (b) the distorted S_1 conformation.

9 ACID plots

ACID plots were generated with the ACID 2.0.0 program kindly provided by Prof. Rainer Herges. They are based on calculations at the CSGT-B3LYP/6-311+G(d,p) level with the IOp(10/93=1) keyword in G09 Revision D.01. The geometries are at the B2PLYP/6-311+G(d,p) level. (Note that CSGT calculations are not possible with B2PLYP in Gaussian.)

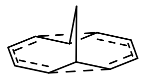

1 T_1

Direction of magnetic field	ACID isosurface at 0.050 a.u.
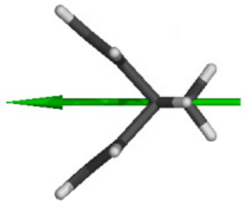	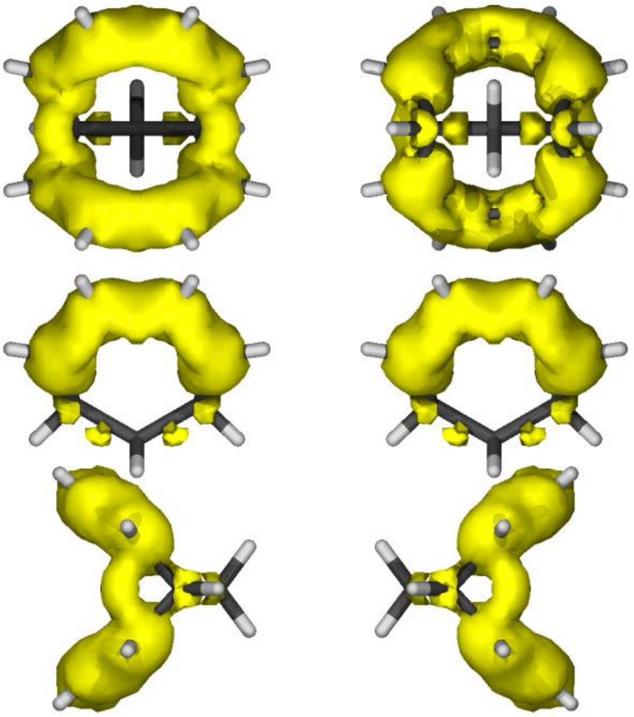

ACID plot with current vectors at 0.047 a.u.

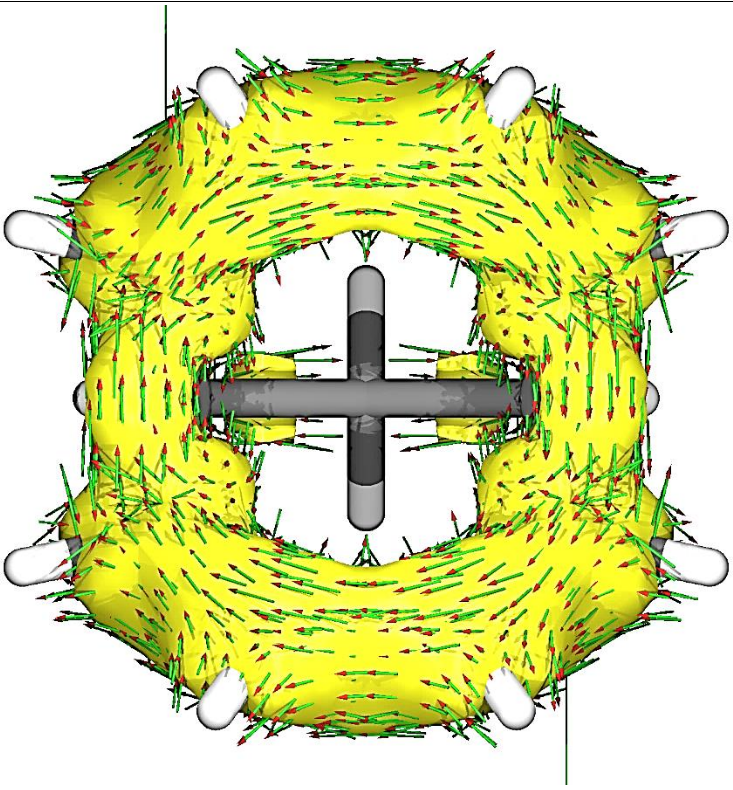

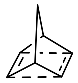

2 **T_1**

Direction of magnetic field	ACID isosurface at 0.050 a.u.
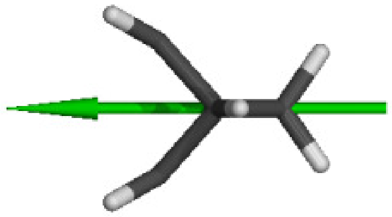	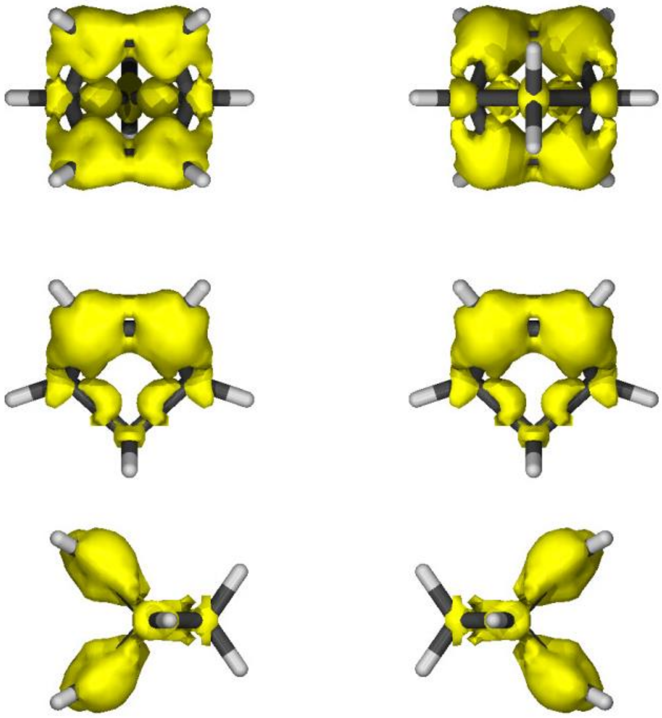

ACID plot with current vectors at

0.020 a.u	0.030 a.u.
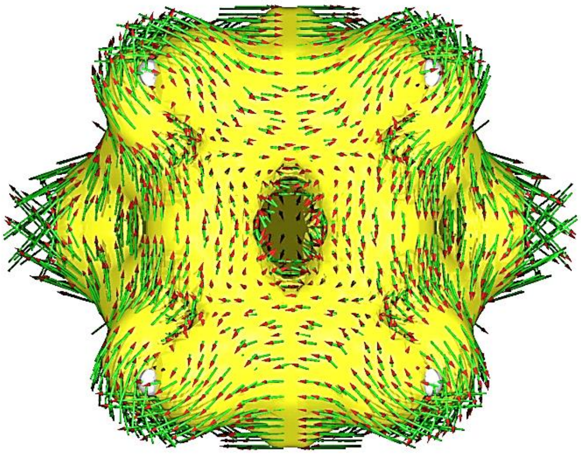	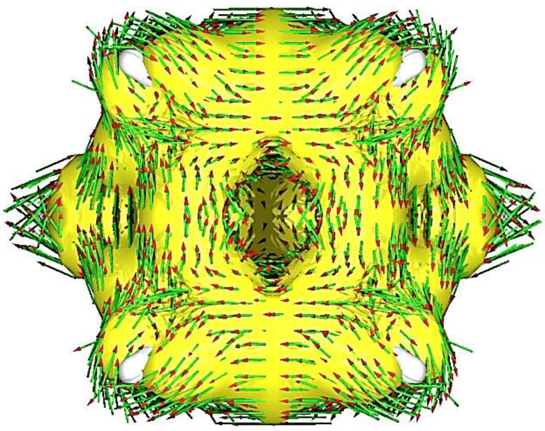

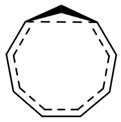

3 **T_1**

Direction of magnetic field	ACID isosurface at 0.050 a.u.	
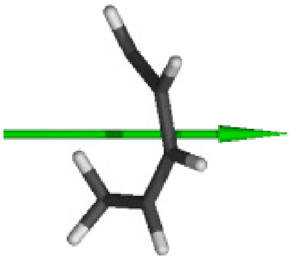	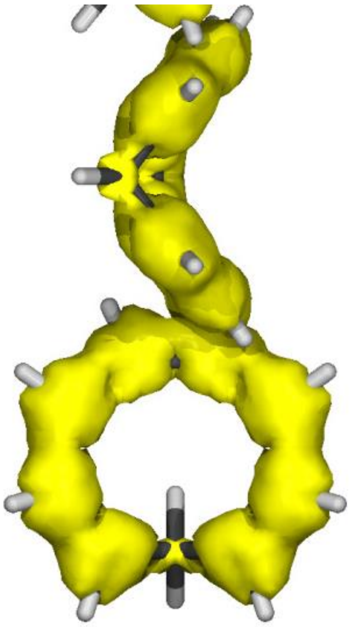	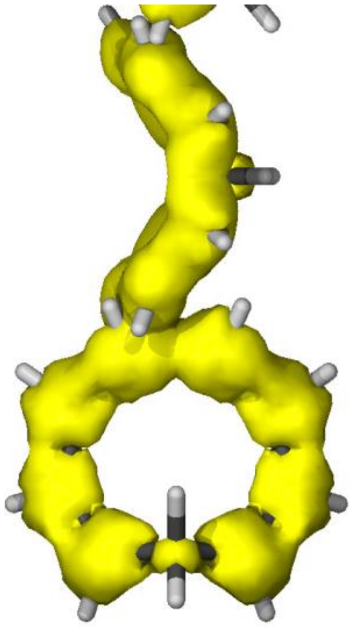

ACID plot with current vectors at

0.020 a.u.	0.030 a.u.
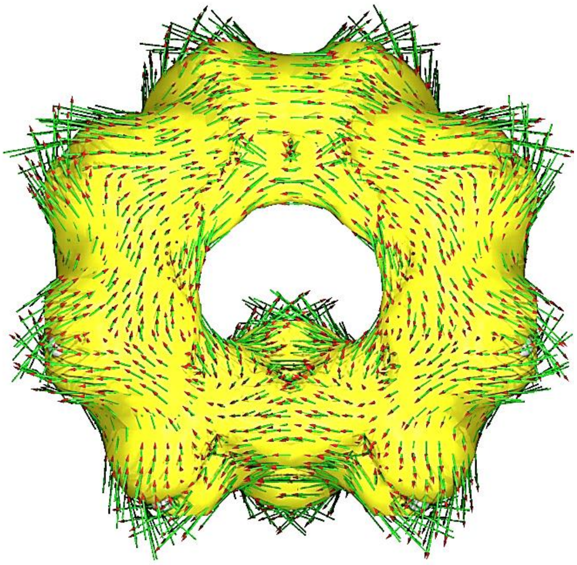	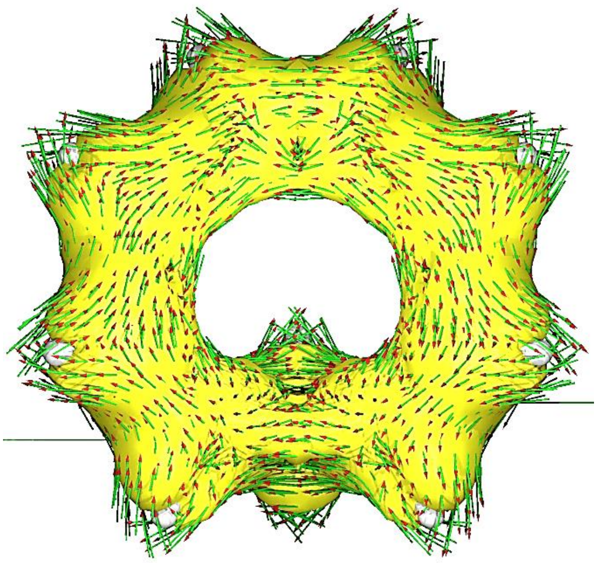

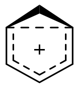

4 **T₁**

Direction of magnetic field	ACID isosurface at 0.050 a.u.

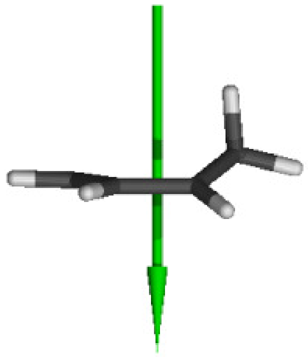

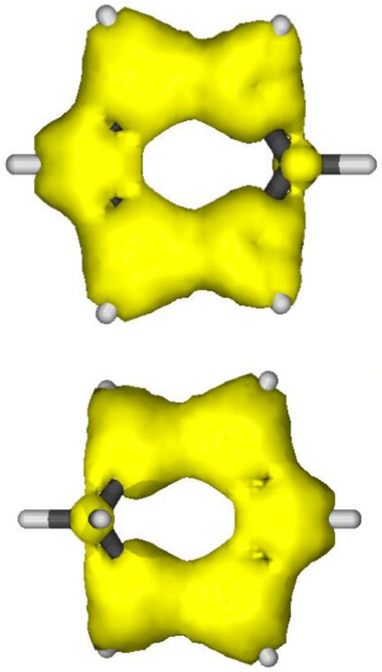

ACID plot with current vectors at 0.020 a.u.

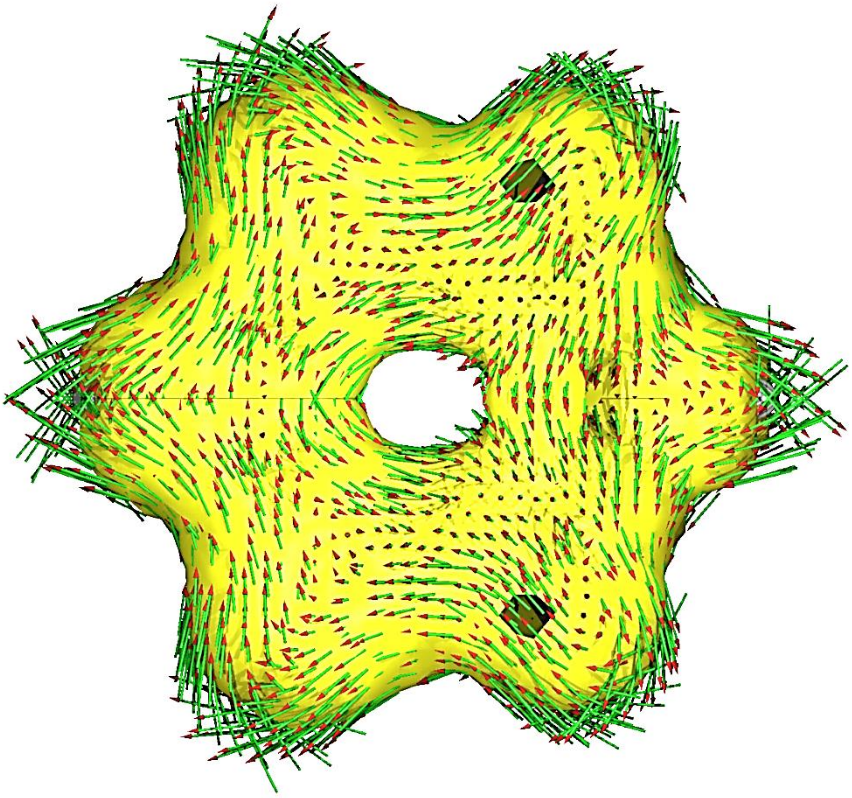

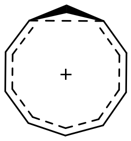

5a T$_1$

Direction of magnetic field ACID isosurface at 0.050 a.u.

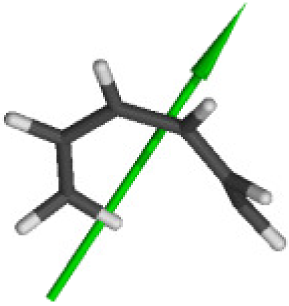

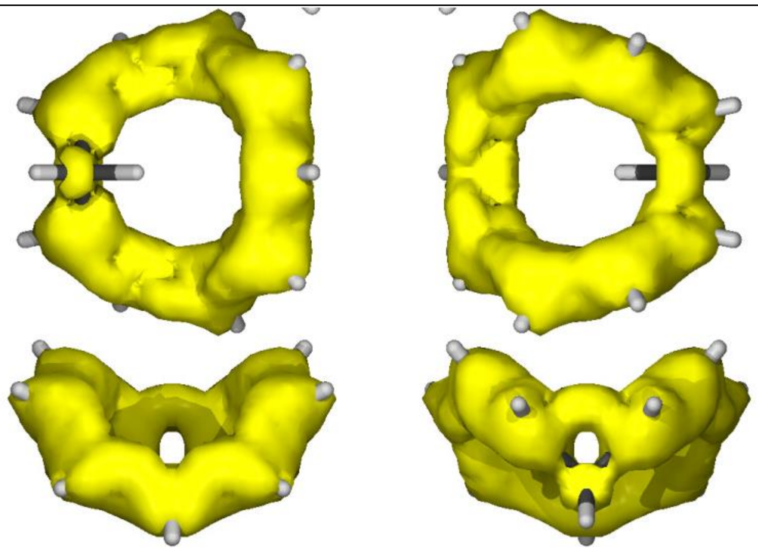

ACID plot with current vectors at 0.050 a.u.

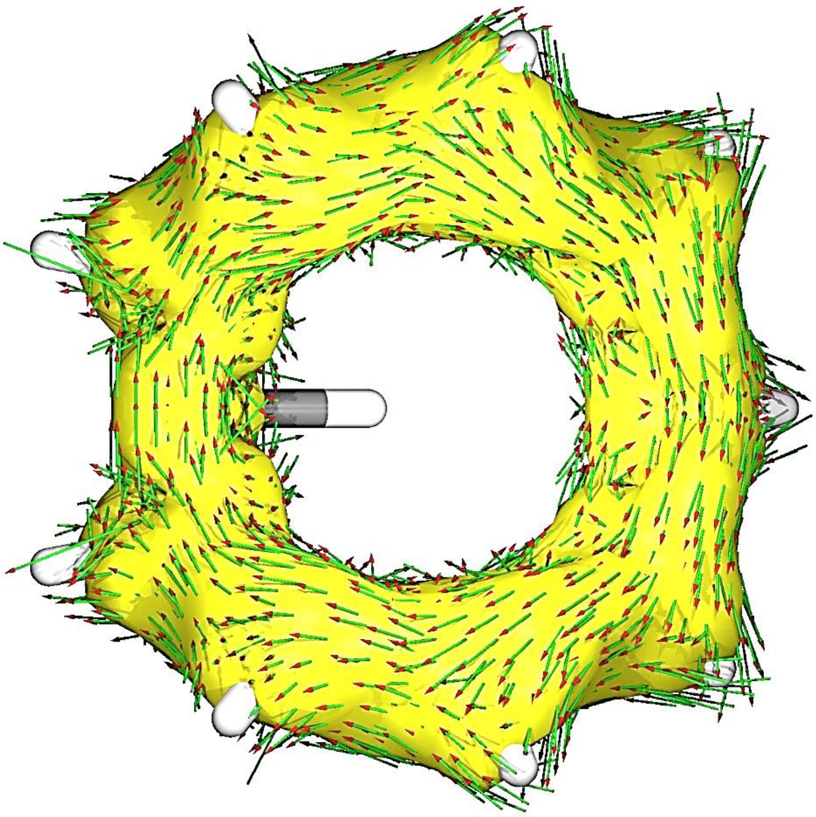

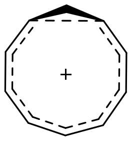

5b **T_1**

Direction of magnetic field ACID isosurface at 0.050 a.u.

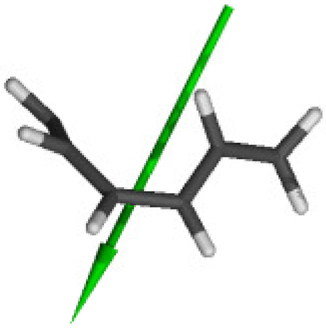

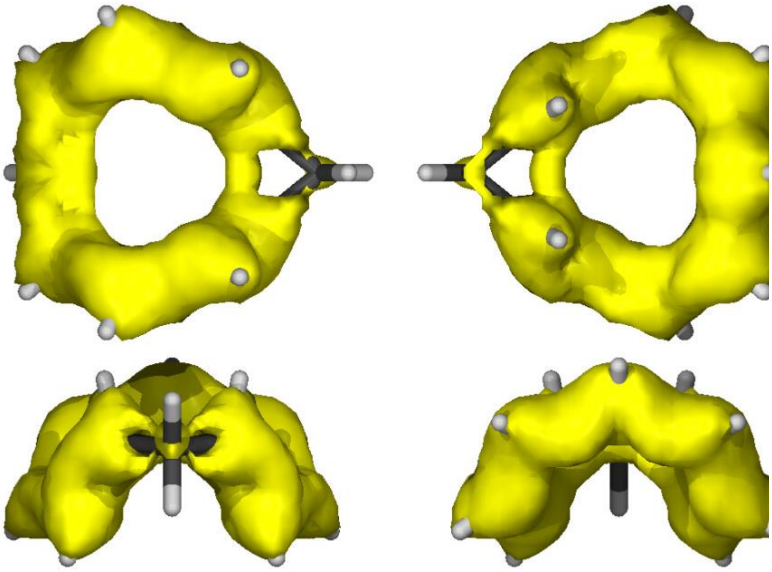

ACID plot with current vectors at 0.050 a.u.

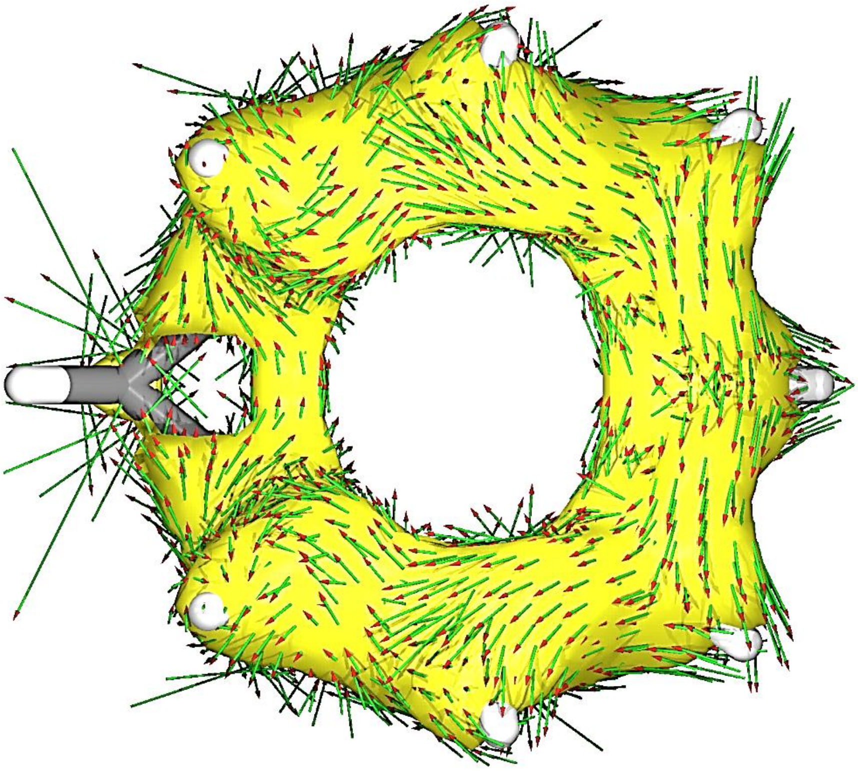

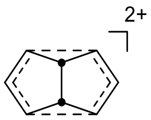

6 **T_1**

Direction of magnetic field ACID isosurface at 0.050 a.u.

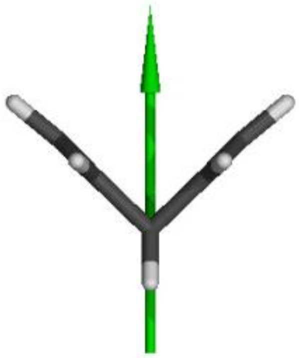

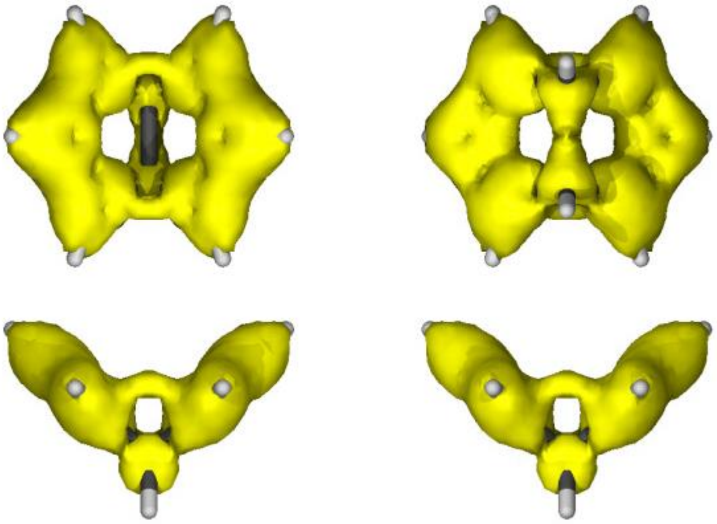

ACID plot with current vectors at 0.050 a.u.

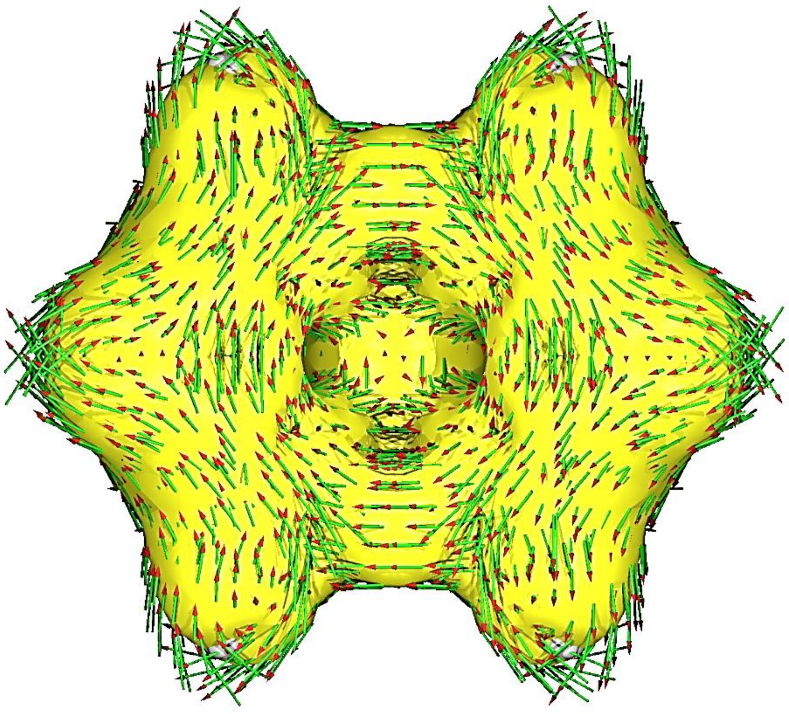

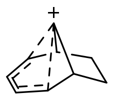

7 **T_1**

Direction of magnetic field	ACID isosurface at 0.050 a.u.
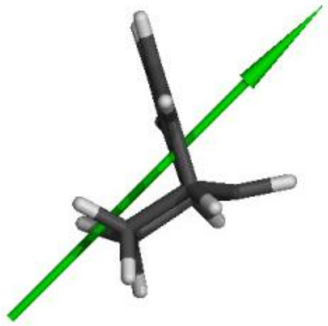	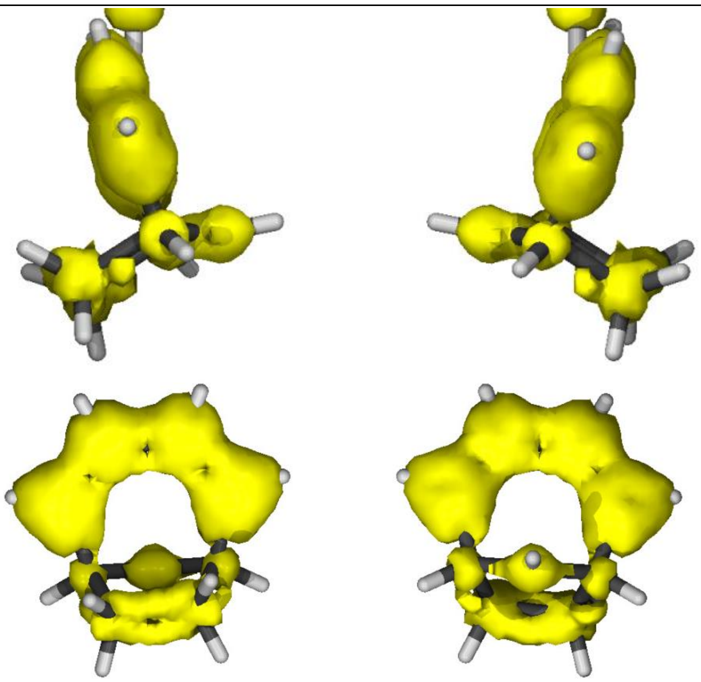

ACID plot with current vectors at

0.020 a.u.	0.030 a.u.
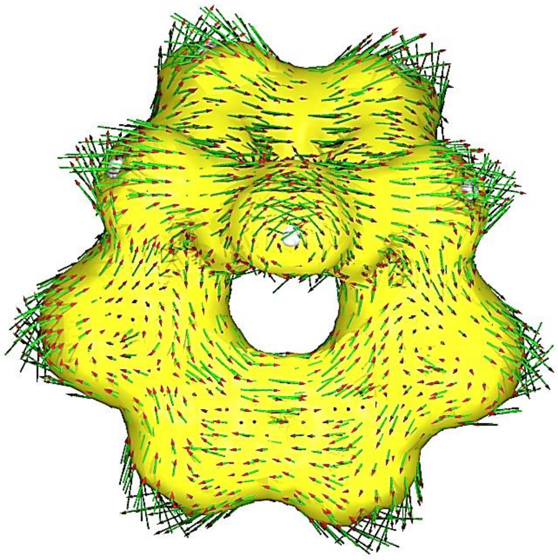	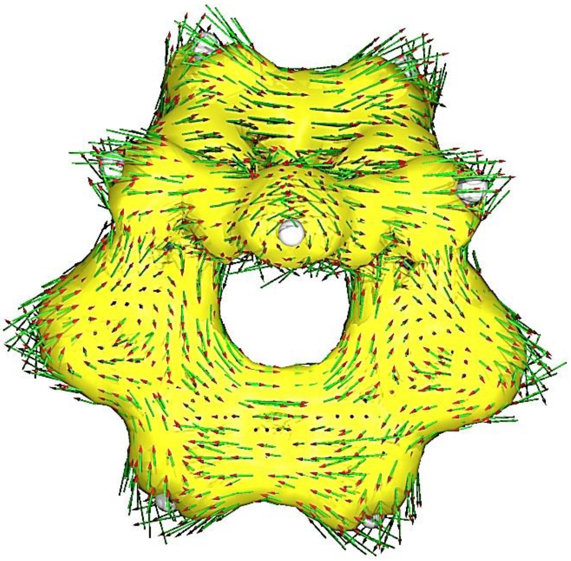

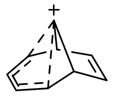

8 T$_1$

Direction of magnetic field	ACID isosurface at 0.050 a.u.
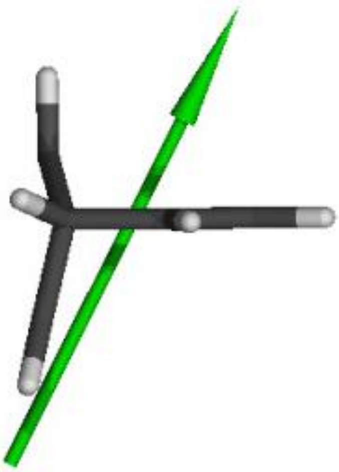	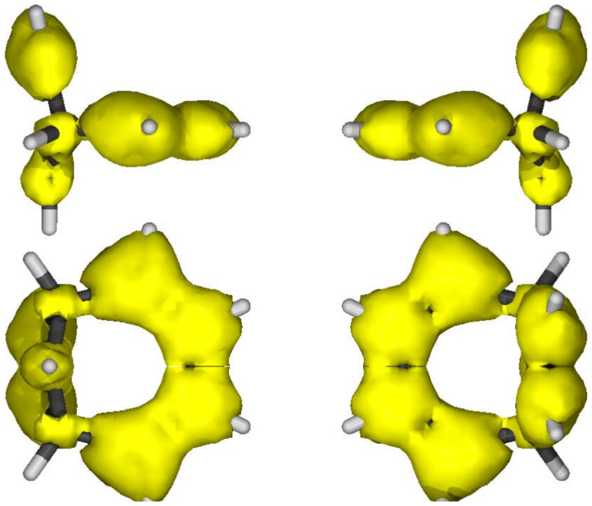

ACID plot with current vectors at	
0.020 a.u.	0.030 a.u.
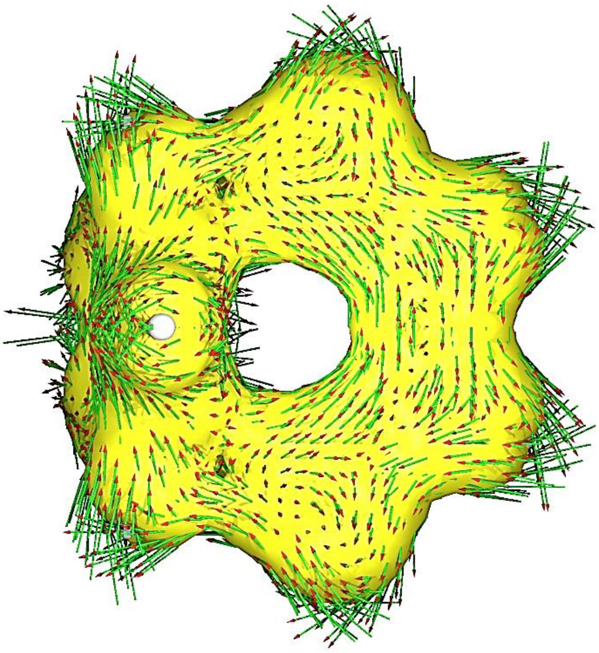	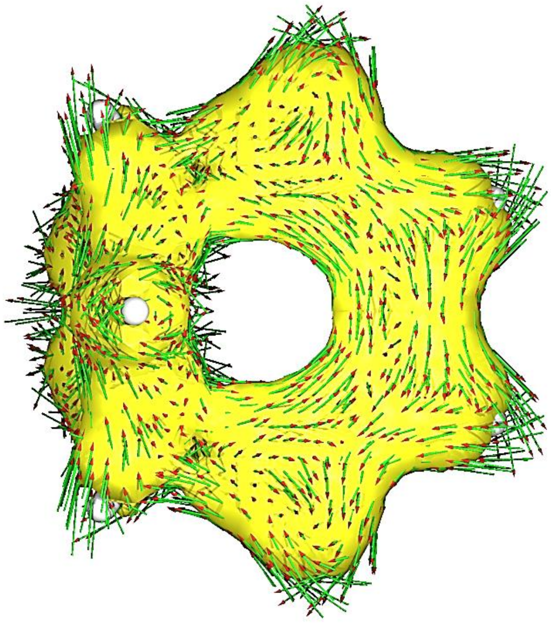

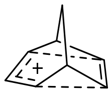

9 **T1**

Direction of magnetic field	ACID isosurface at 0.050 a.u.

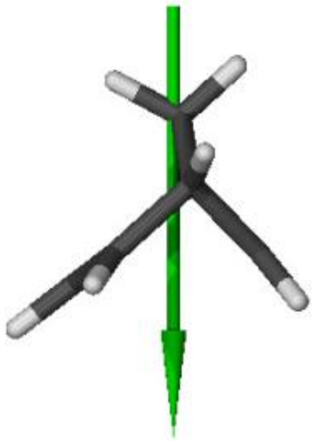

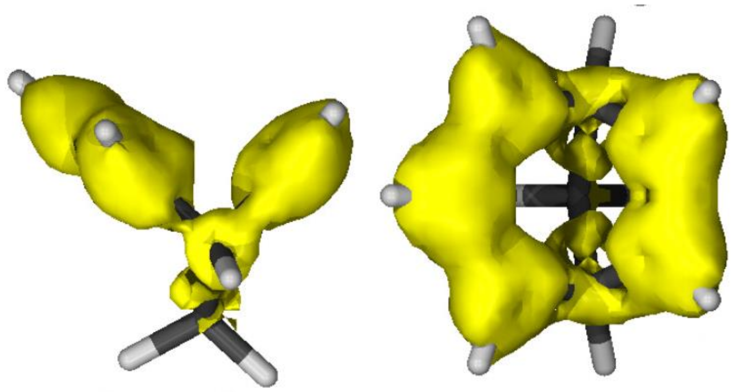

ACID plot with current vectors at	
0.020 a.u.	0.030 a.u.

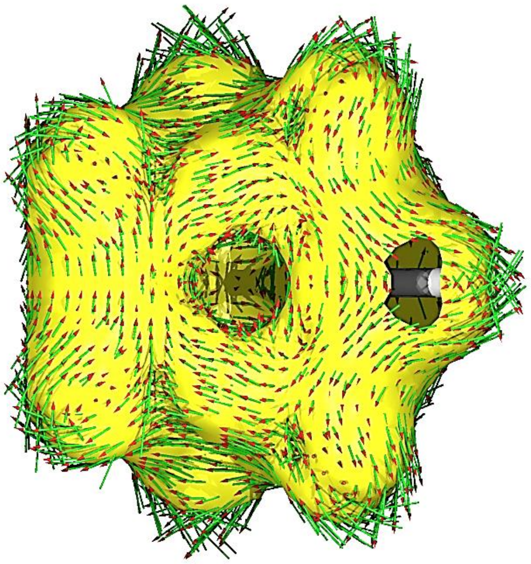

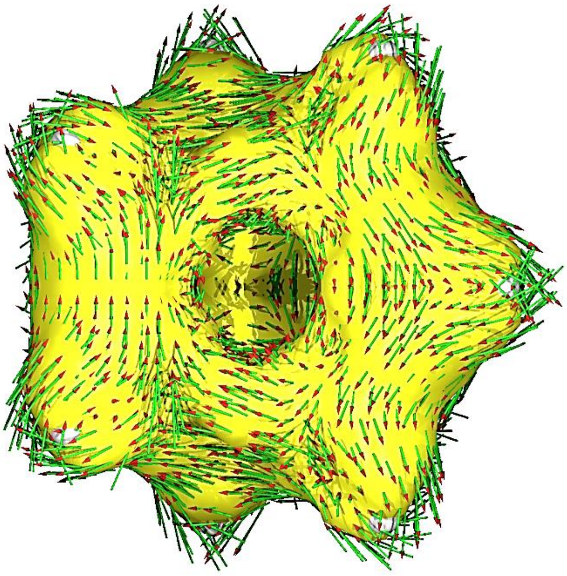

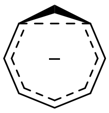

10 **T1**

Direction of magnetic field	ACID isosurface at 0.050 a.u.
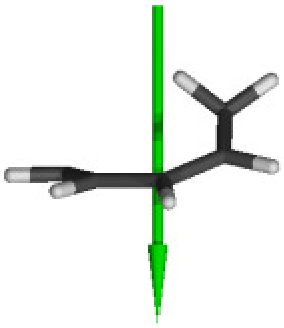	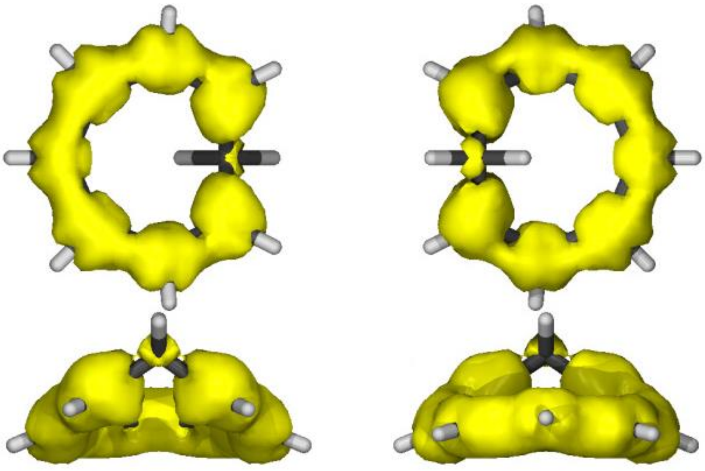

ACID plot with current vectors at

0.020 a.u.	0.030 a.u.
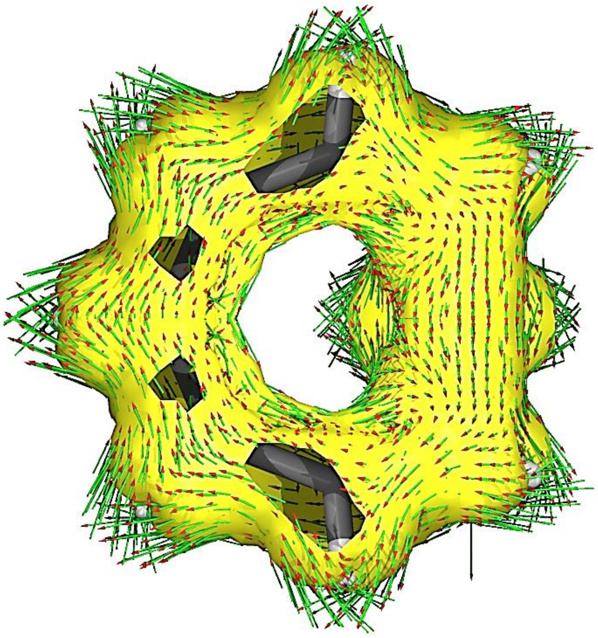	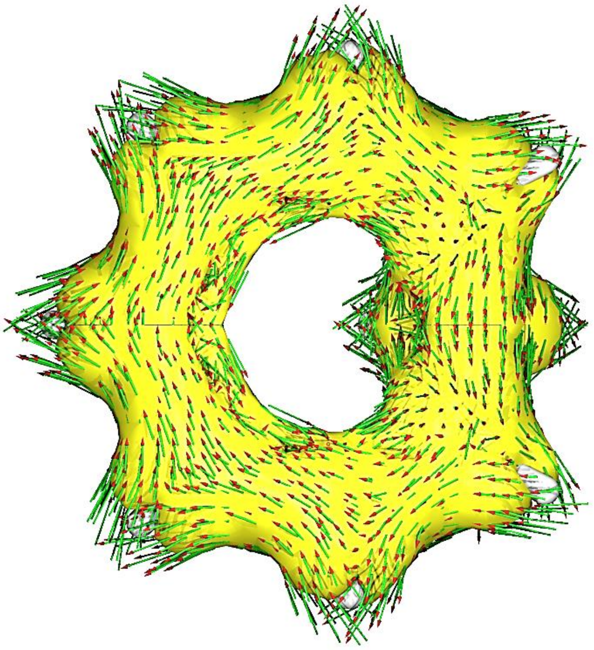

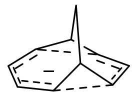

11 **T_1**

Direction of magnetic field	ACID isosurface at 0.050 a.u.
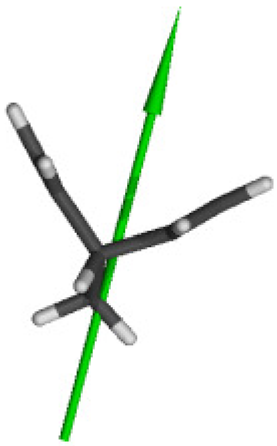	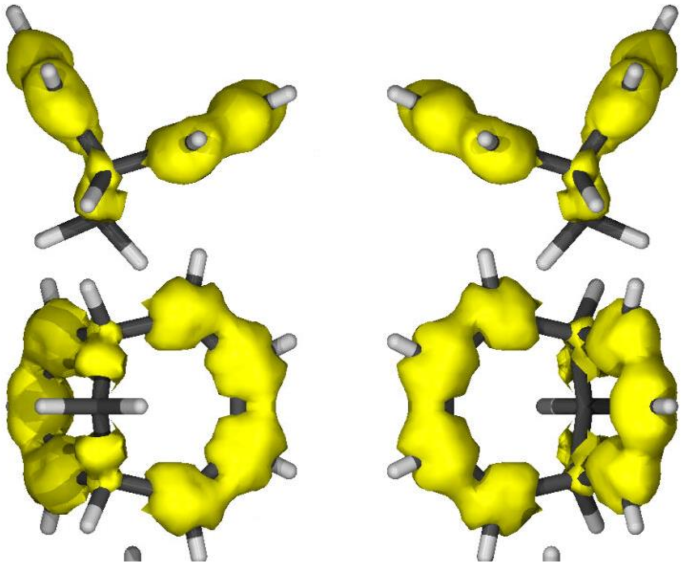

ACID plot with current vectors at	
0.020 a.u.	0.030 a.u.
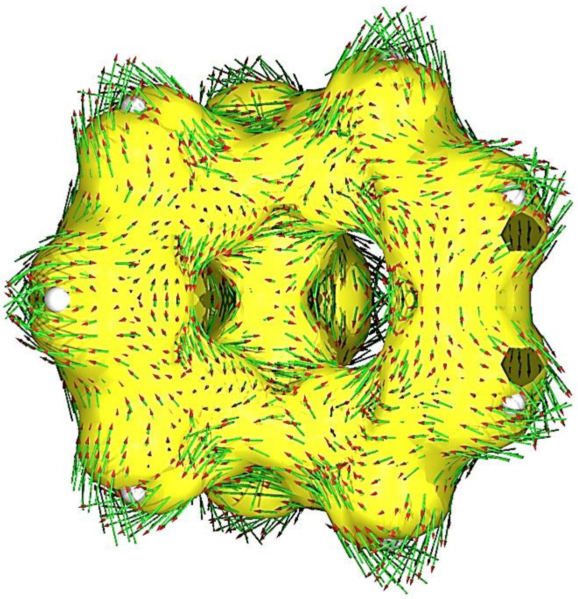	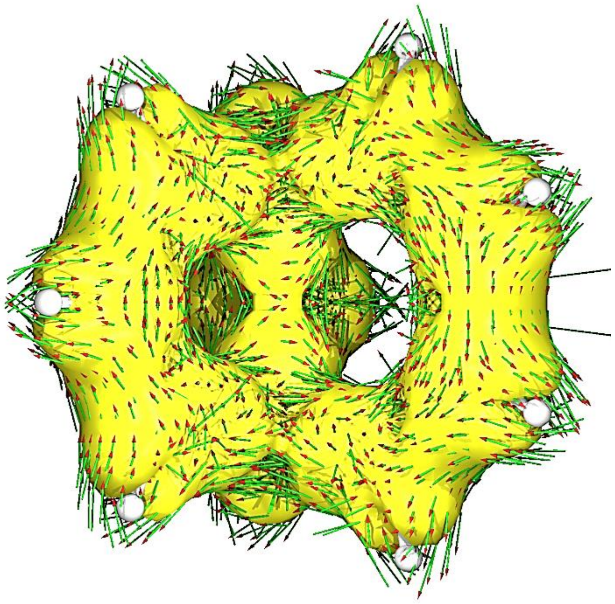

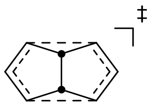

12 S0

Direction of magnetic field ACID isosurface at 0.050 a.u.

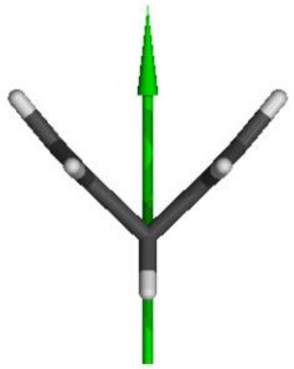

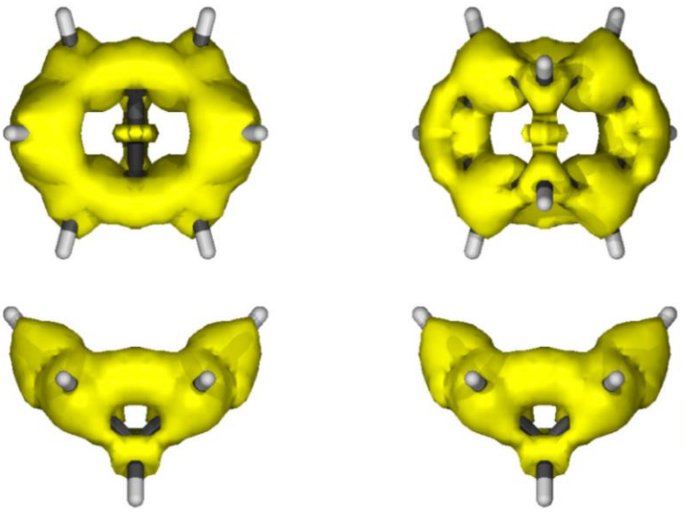

ACID plot with current vectors at 0.050 a.u.

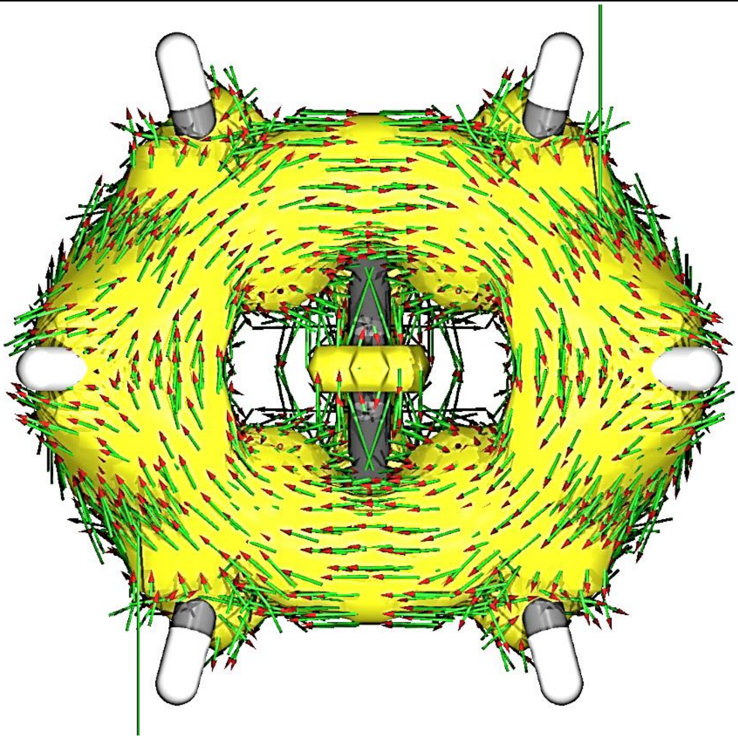

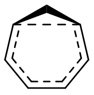

13 **S_0**

Direction of magnetic field	ACID isosurface at 0.050 a.u.
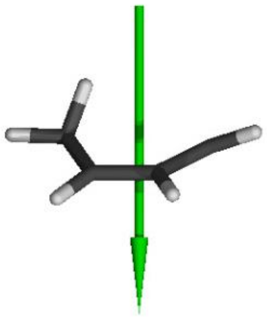	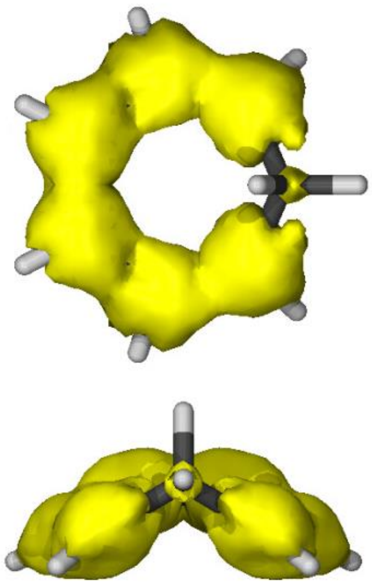

ACID plot with current vectors at 0.020 a.u.

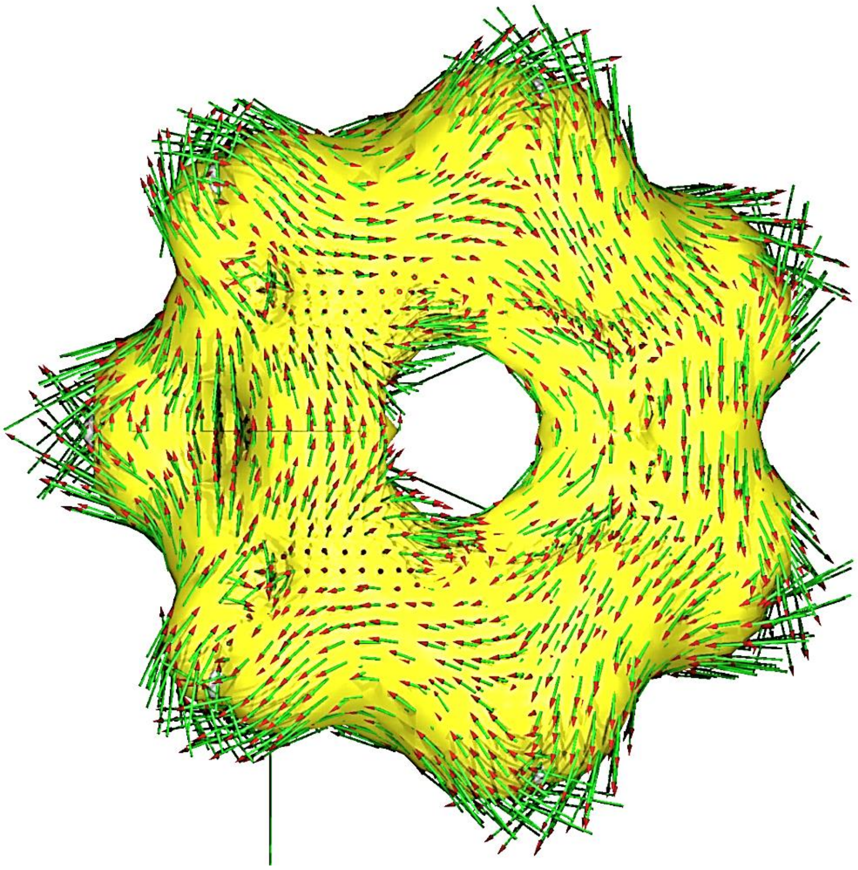

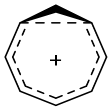

14 **S_0**

Direction of magnetic field	ACID isosurface at 0.050 a.u.

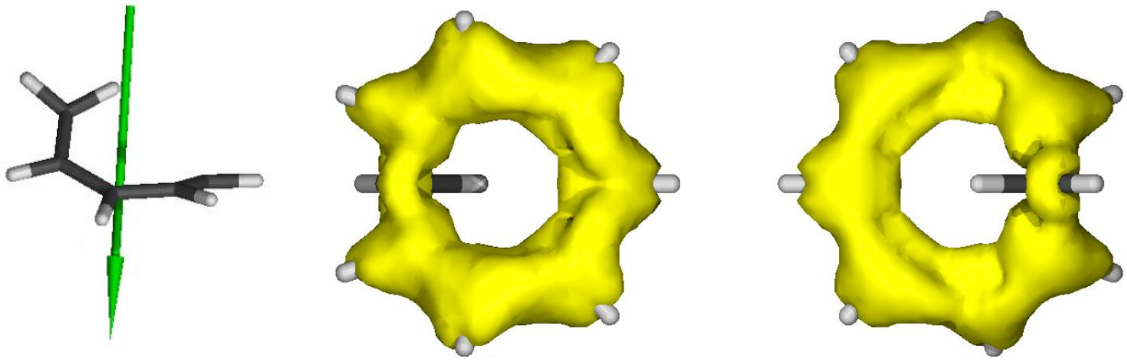

ACID plot with current vectors at 0.050 a.u.

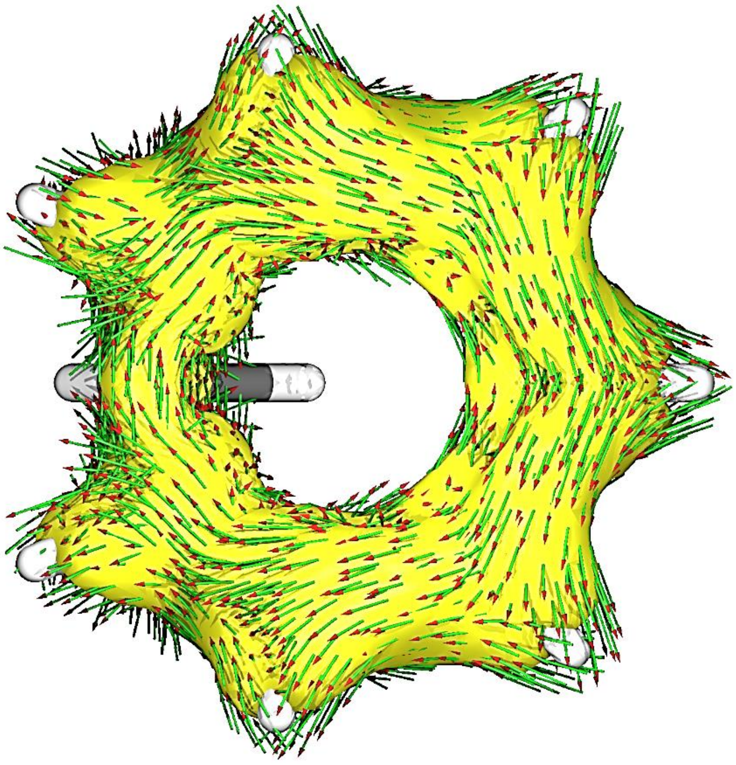

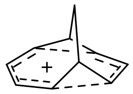

15 **S_0**

Direction of magnetic field	ACID isosurface at 0.050 a.u.
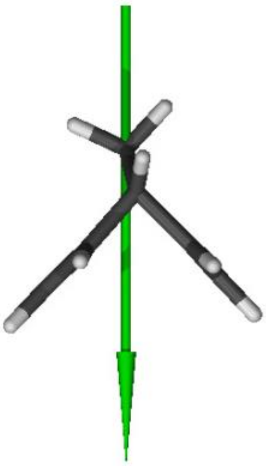	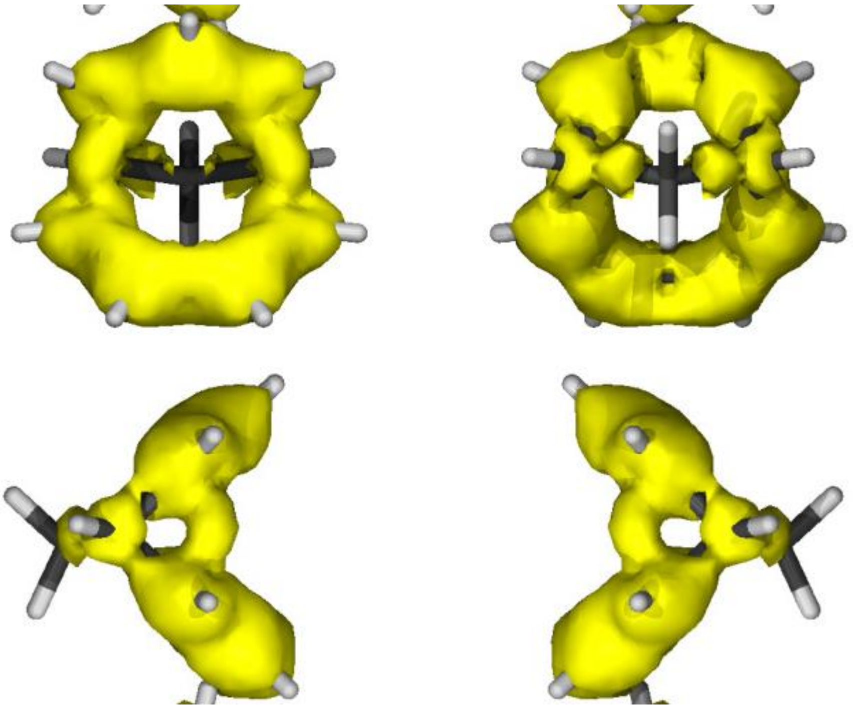

ACID plot with current vectors at 0.050 a.u.

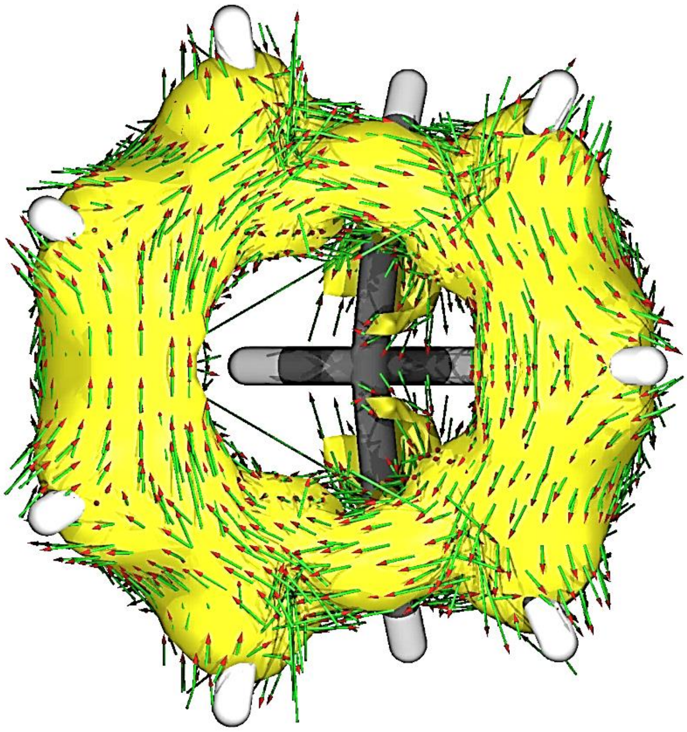

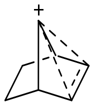

16

Direction of magnetic field	ACID isosurface at 0.050 a.u.
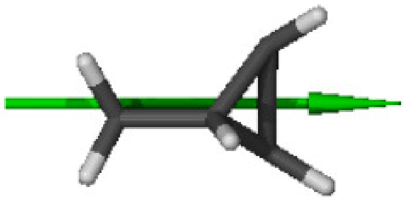	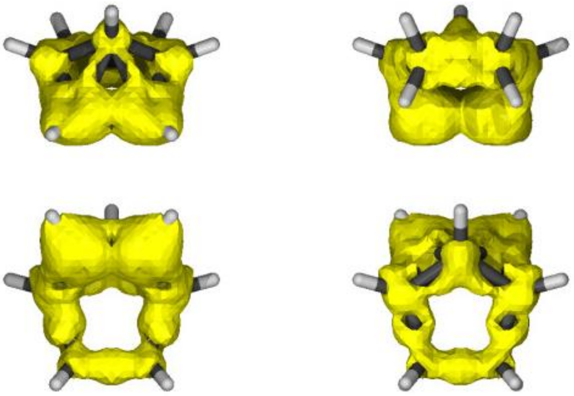

ACID plot with current vectors at

0.030 a.u.	0.040 a.u.
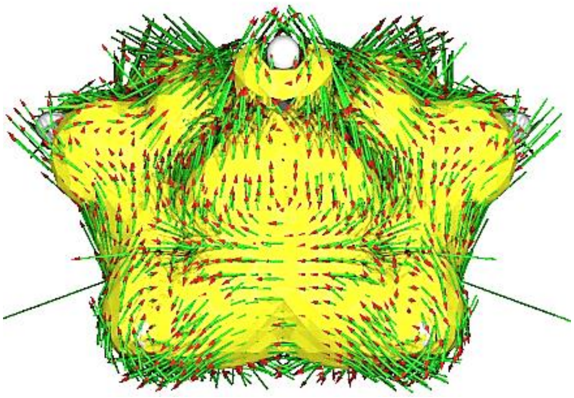	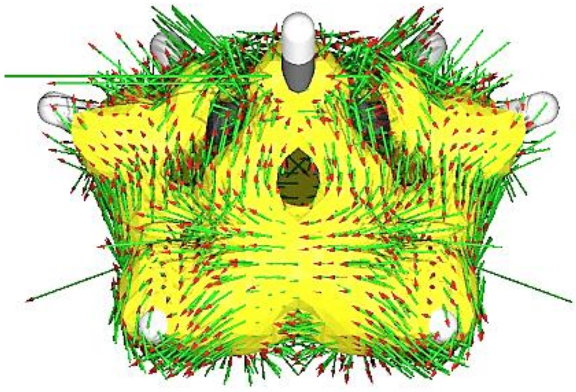

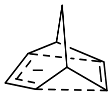

17 S_0

Direction of magnetic field | ACID isosurface at 0.050 a.u.

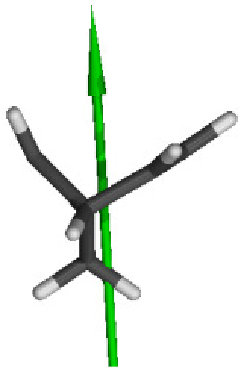

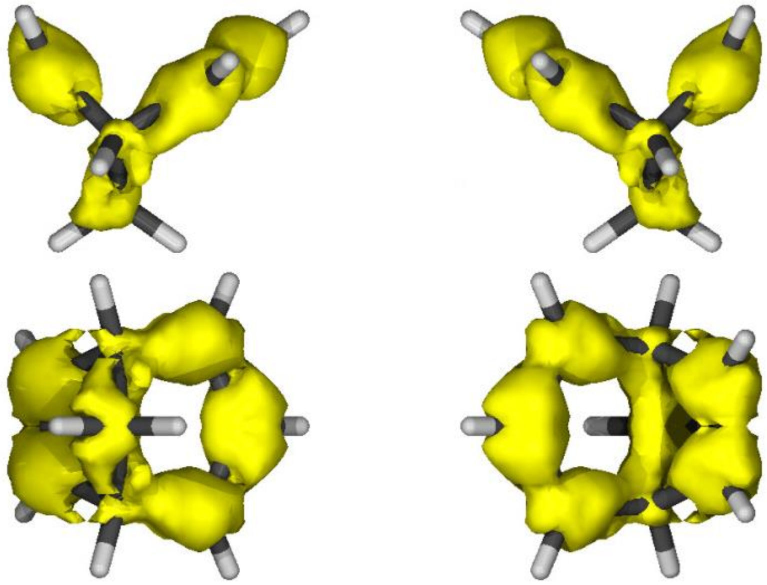

ACID plot with current vectors at

0.020 a.u. | 0.030 a.u.

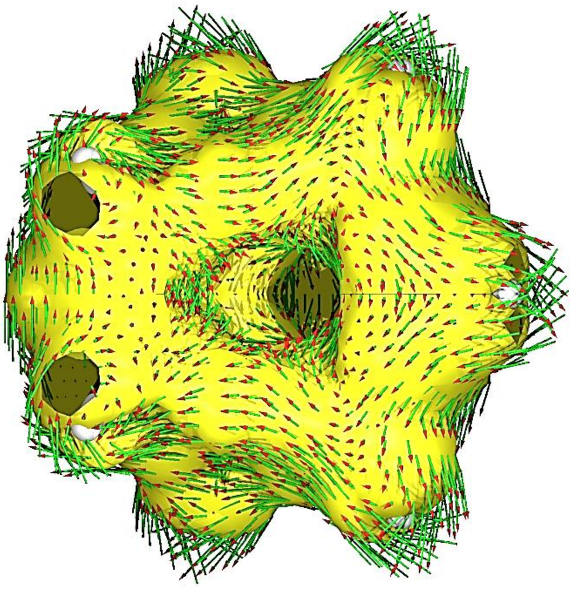

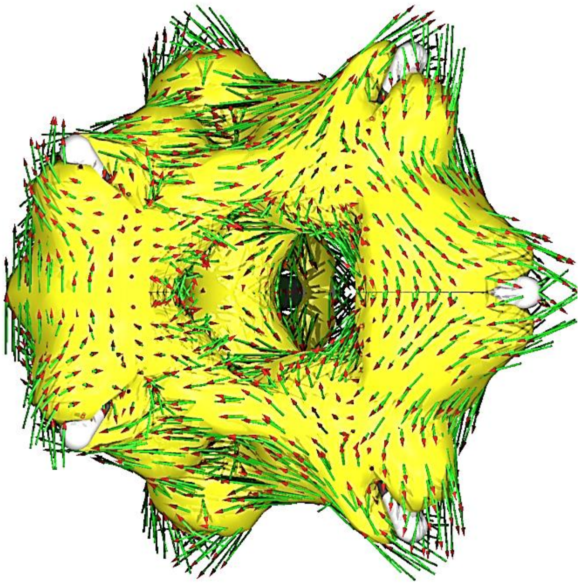

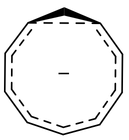

18 **S_0**

Direction of magnetic field ACID isosurface at 0.050 a.u.

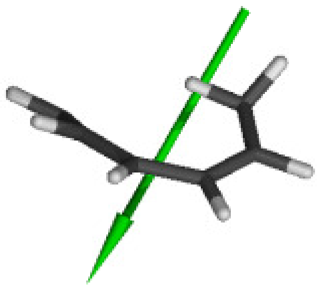

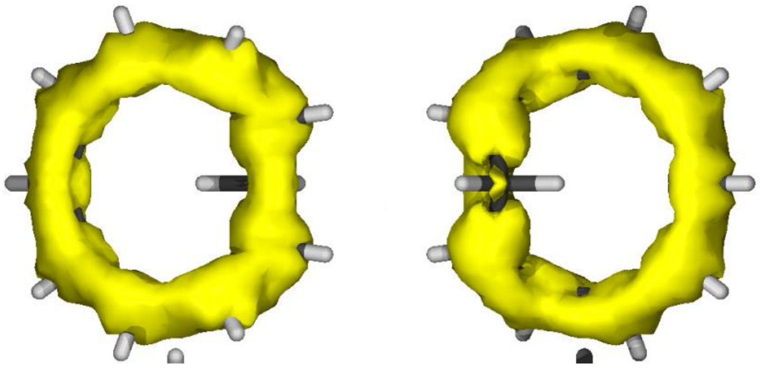

ACID plot with current vectors at 0.050 a.u.

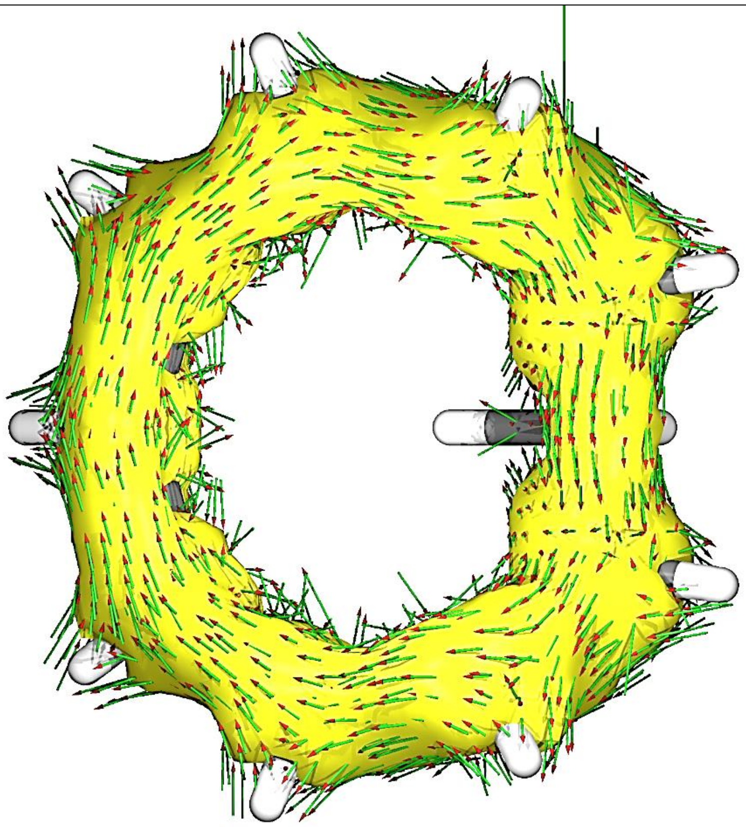

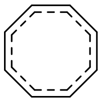

19 **T_1**

Direction of magnetic field ACID isosurface at 0.050 a.u.

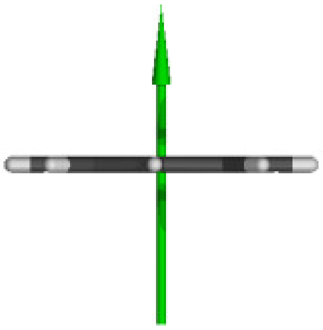

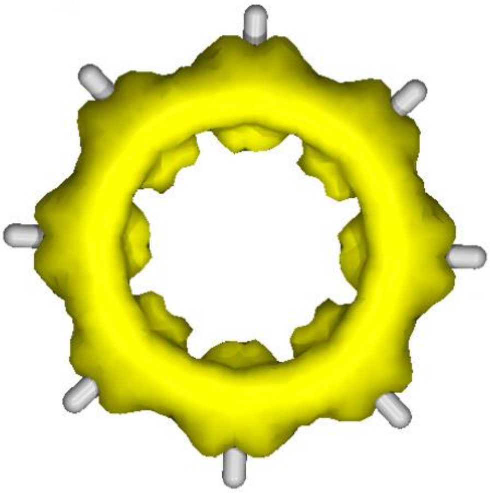

ACID plot with current vectors at 0.050 a.u.

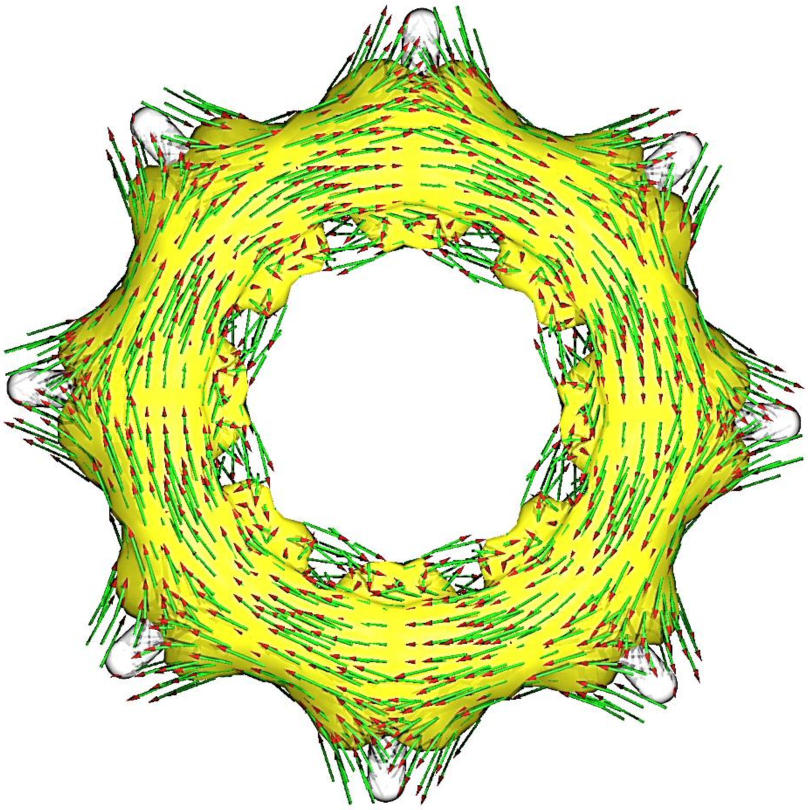

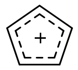

20 **T_1**

Direction of magnetic field	ACID isosurface at 0.050 a.u.
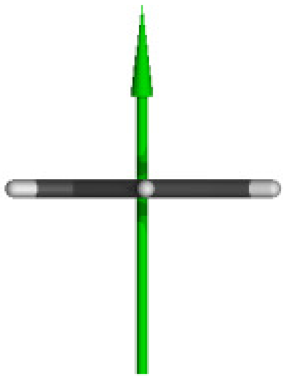	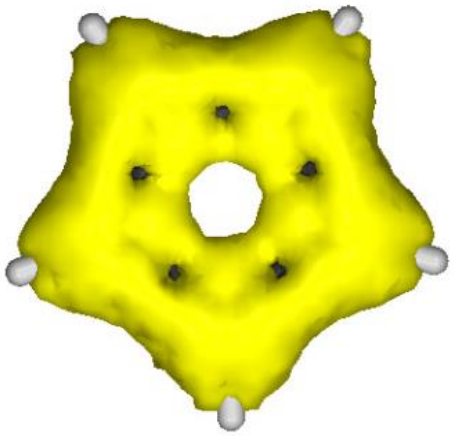

ACID plot with current vectors at 0.050 a.u.

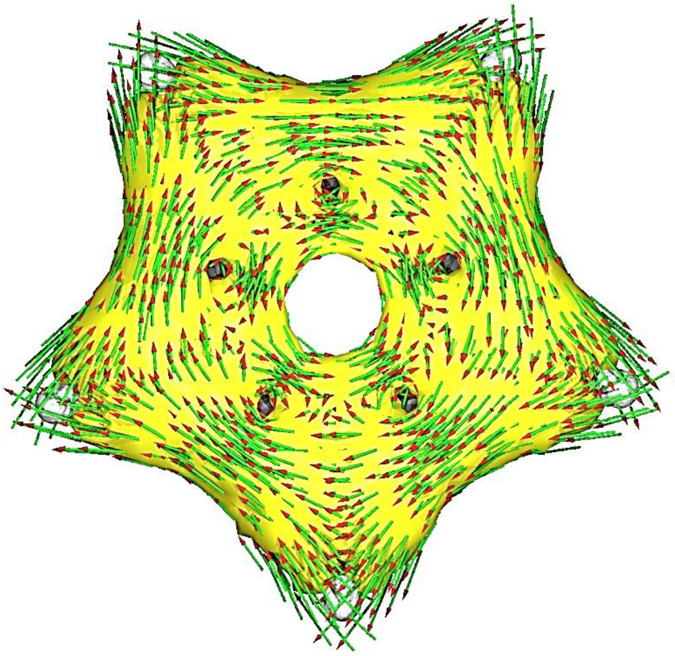

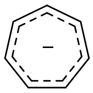

21 **T_1**

Direction of magnetic field ACID isosurface at 0.050 a.u.

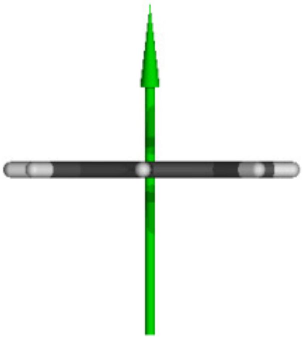

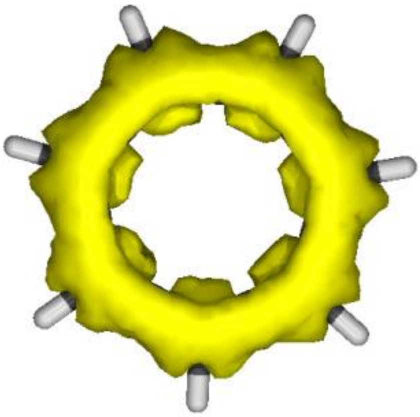

ACID plot with current vectors at 0.050 a.u.

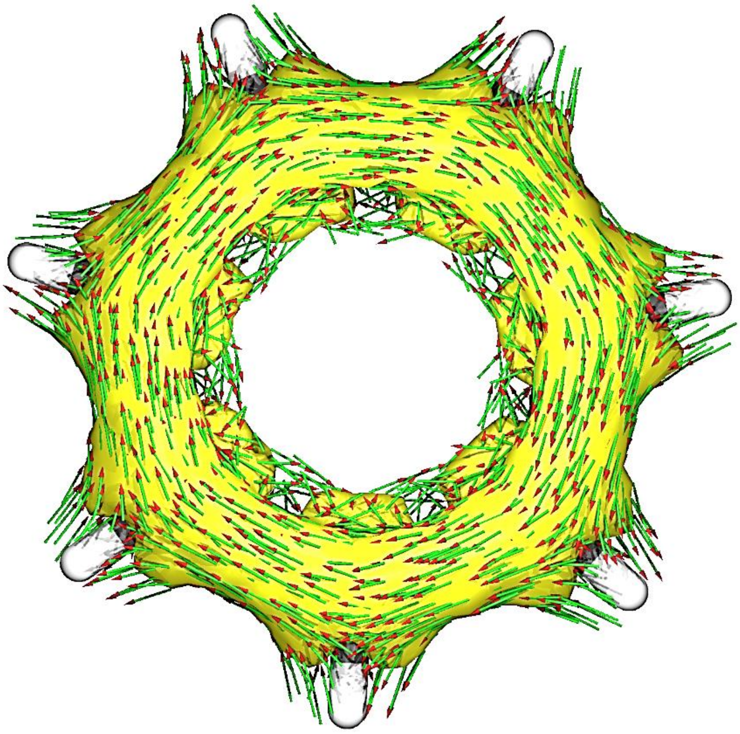

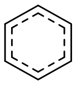

22 **S_0**

Direction of magnetic field ACID isosurface at 0.050 a.u.

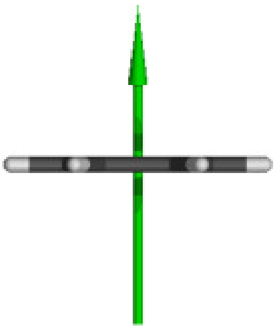

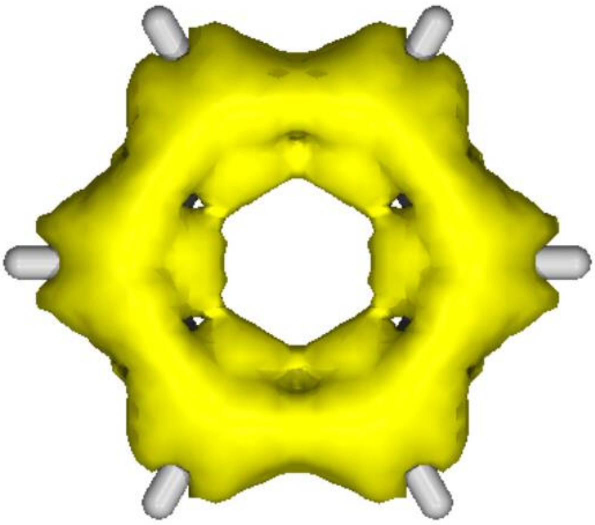

ACID plot with current vectors at 0.050 a.u.

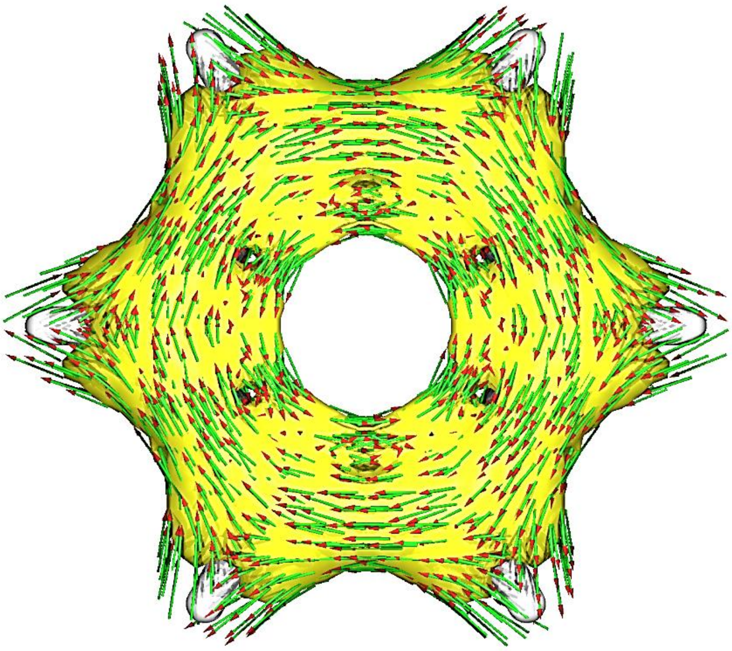

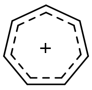

23 S_0

Direction of magnetic field	ACID isosurface at 0.050 a.u.
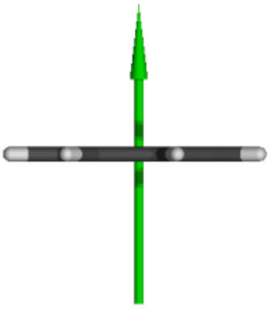	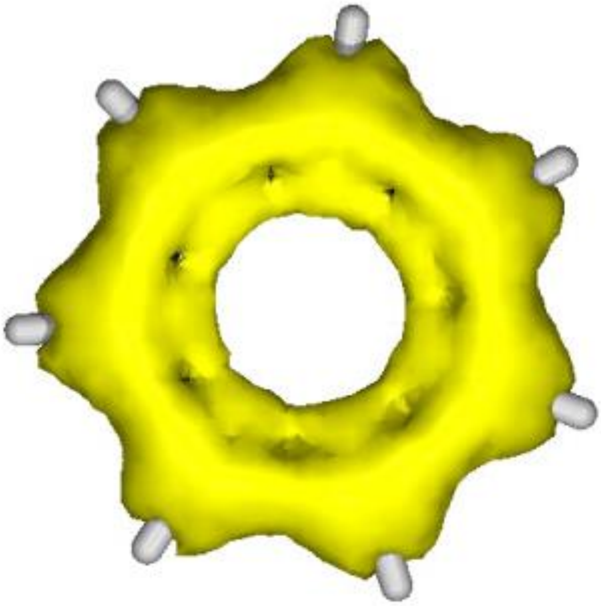

ACID plot with current vectors at 0.050 a.u.

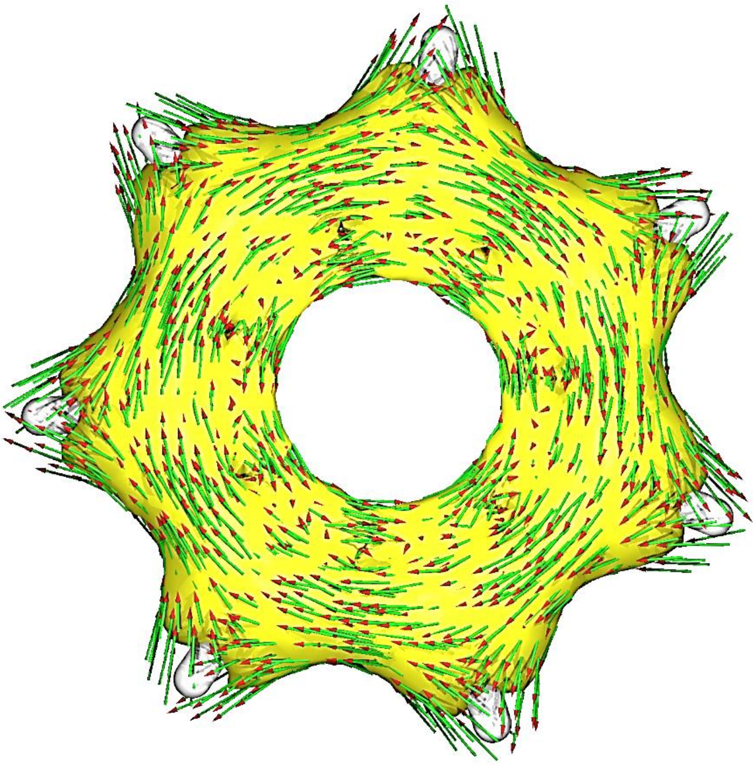

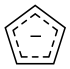

24 S_0

Direction of magnetic field	ACID isosurface at 0.050 a.u.
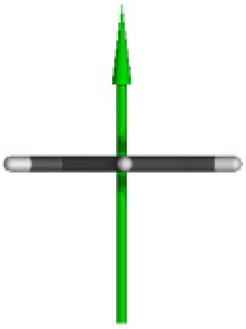	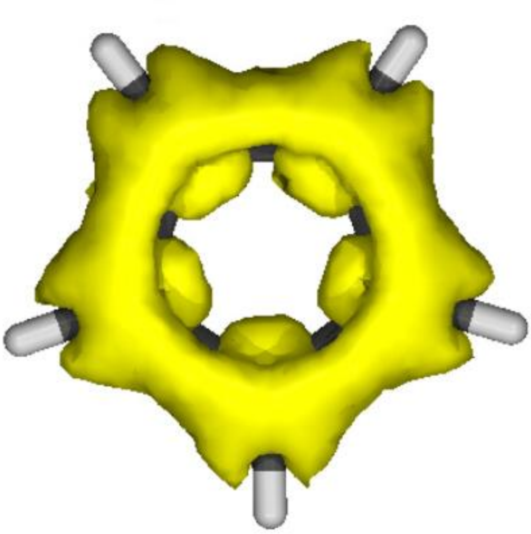

ACID plot with current vectors at 0.050 a.u.

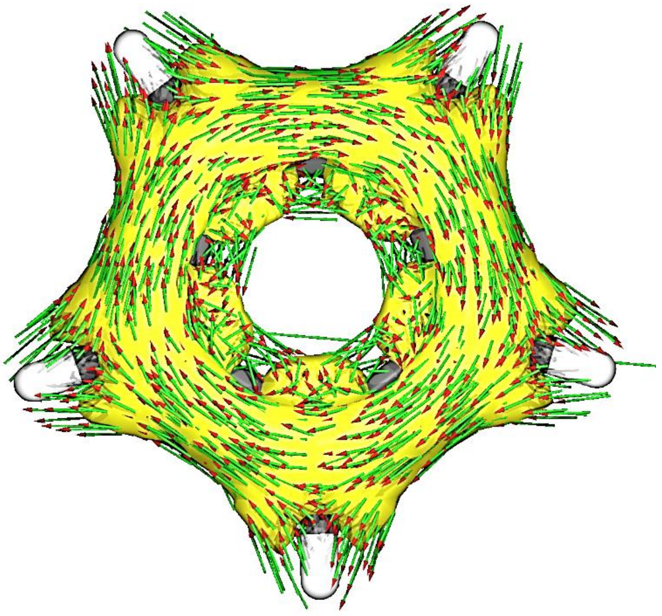

10 Spin densities

Spin densities were calculated and visualized based on the B2PLYP/6-311+G(d,p) densities using GaussView 5.0.[4] They are shown at 0.0004 a.u. isosurface.

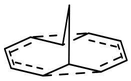

1a

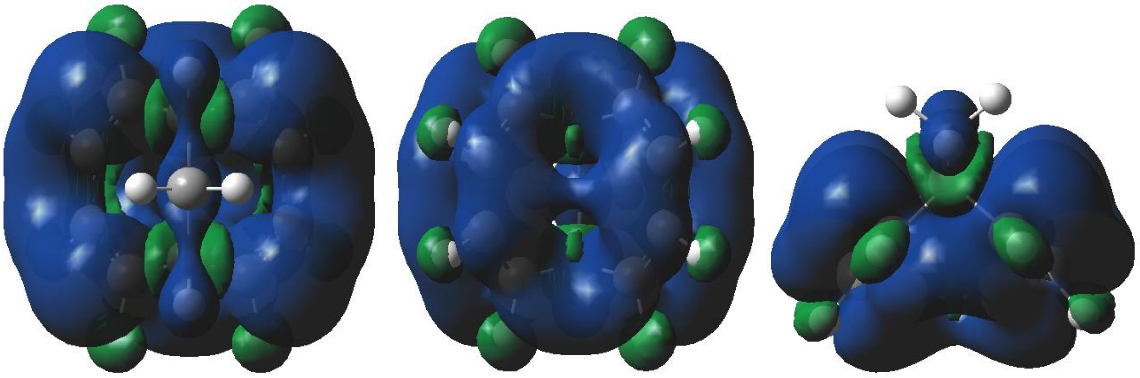

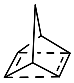

2a

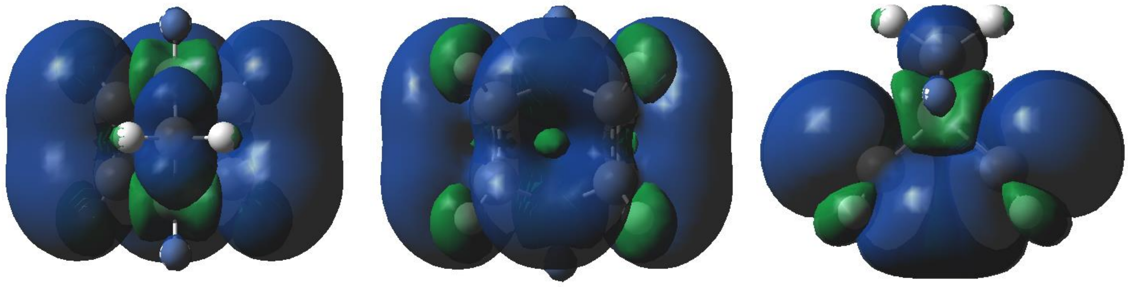

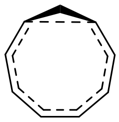

3

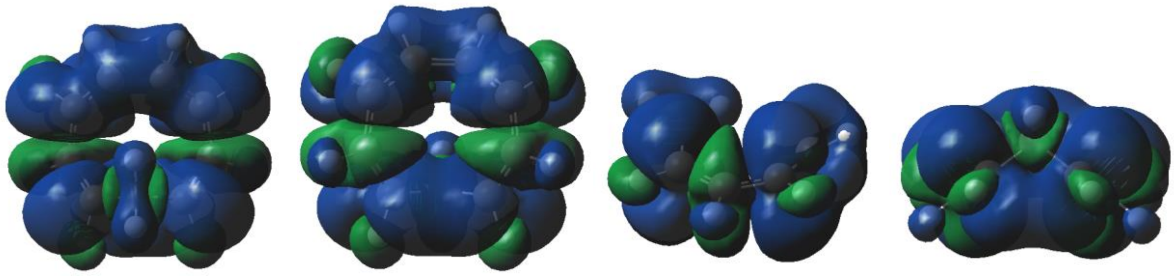

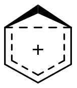

4

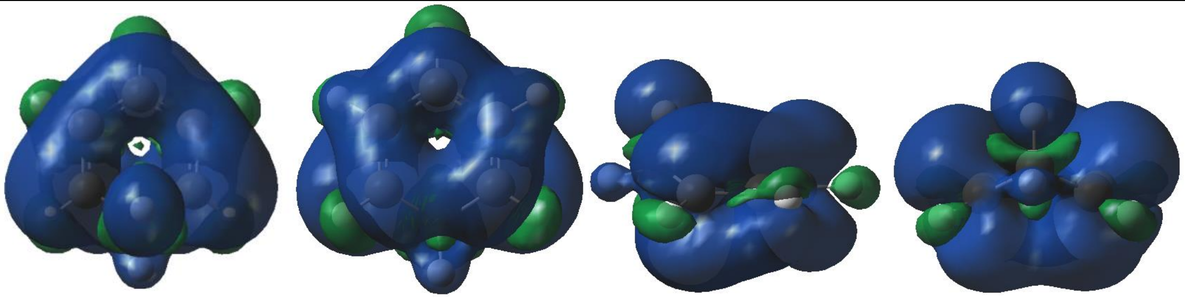

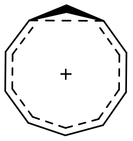

5a

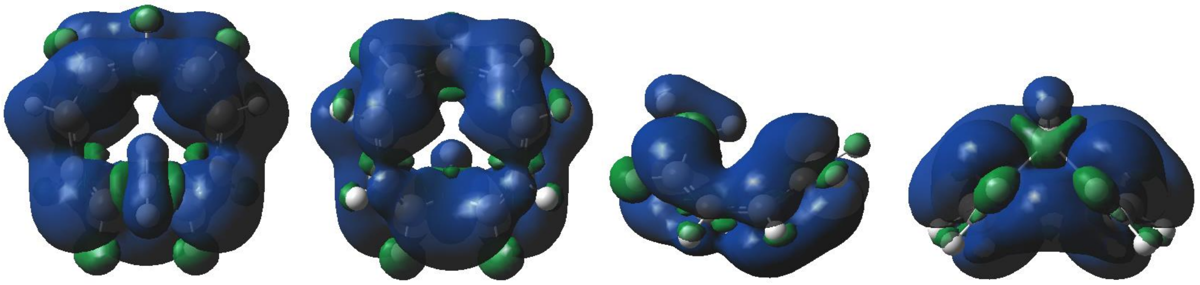

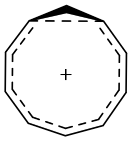

5b

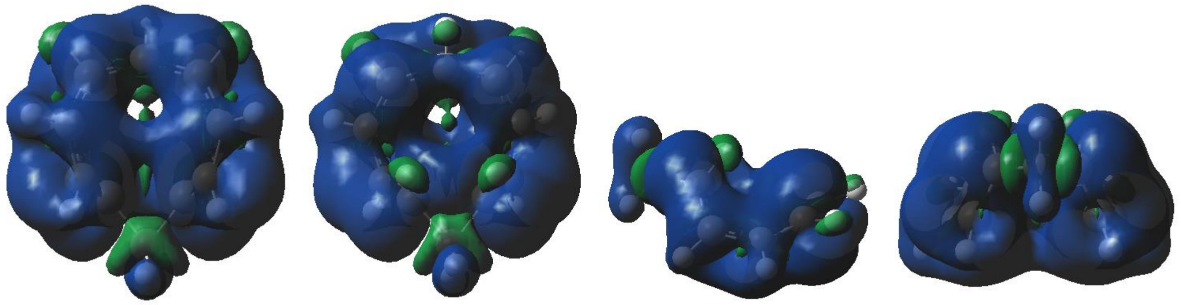

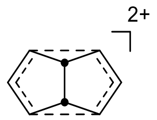

6

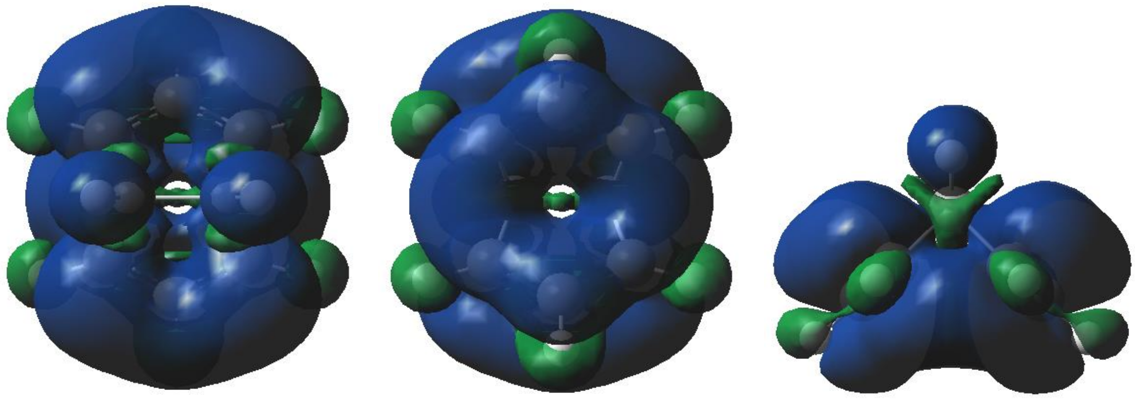

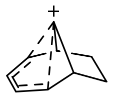

7

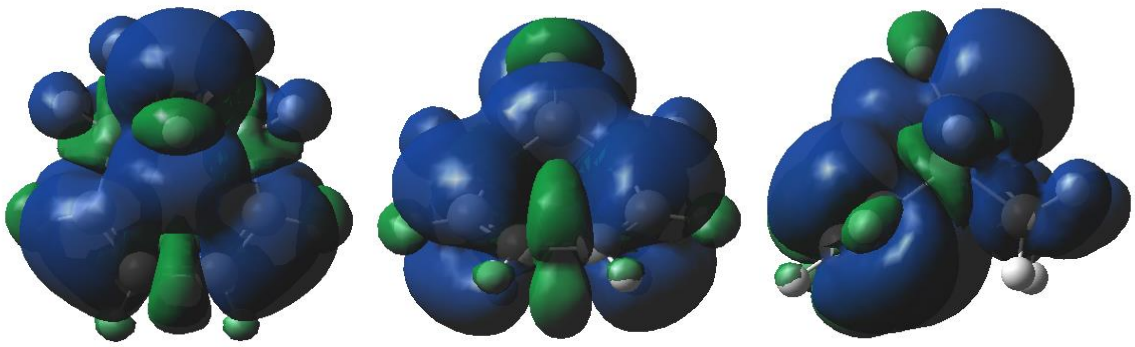

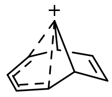

8a

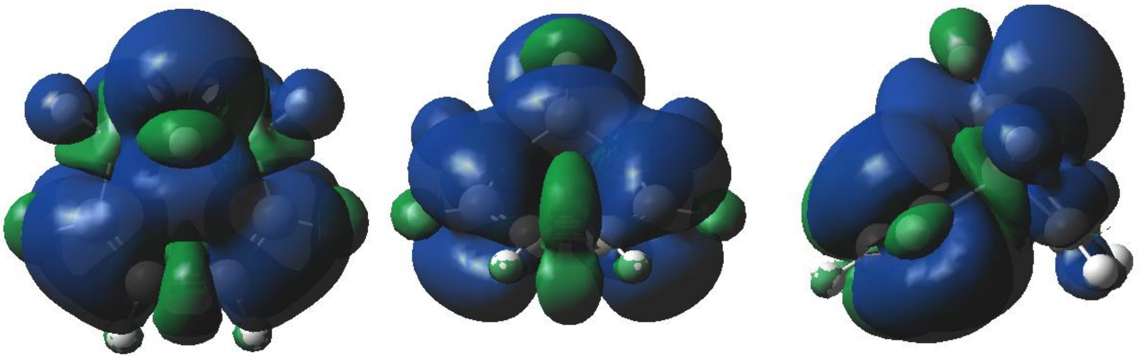

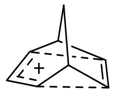

9

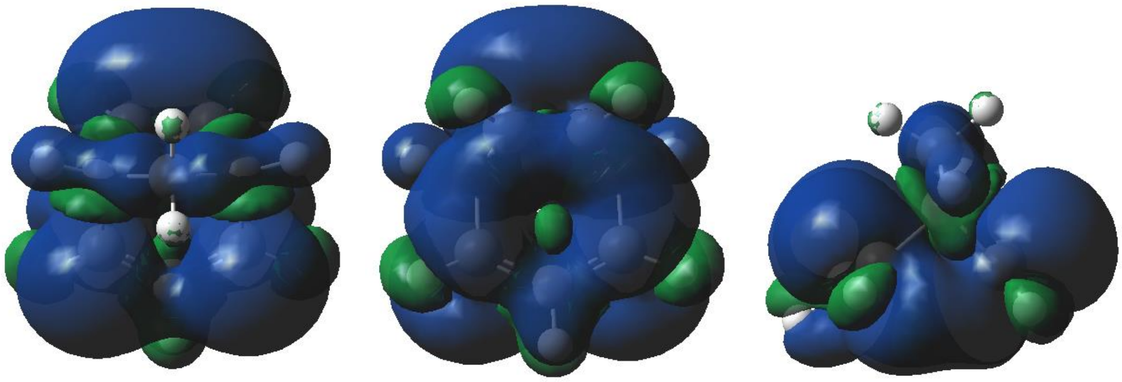

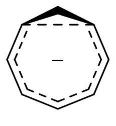

10

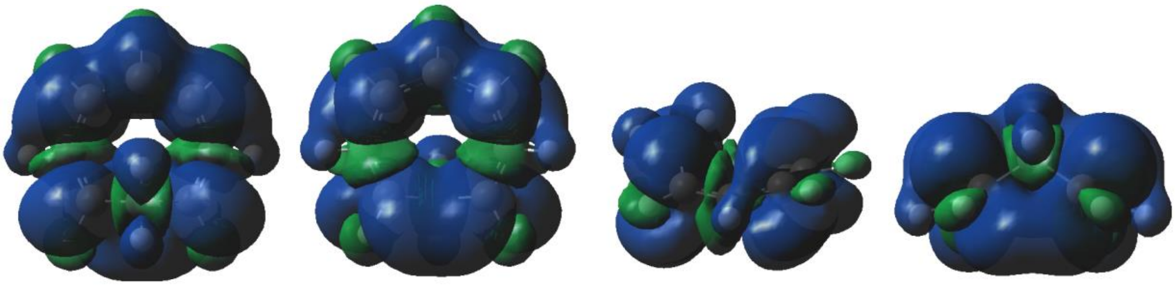

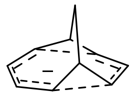

11

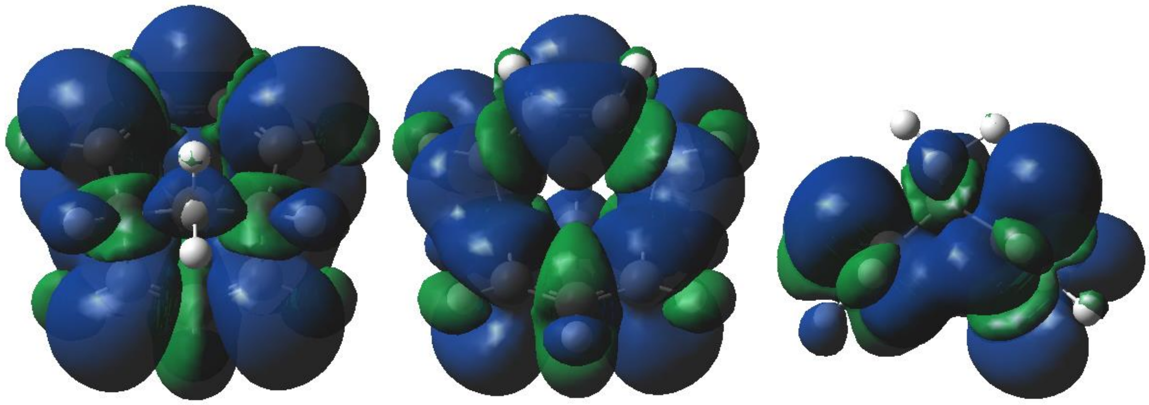

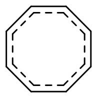

19

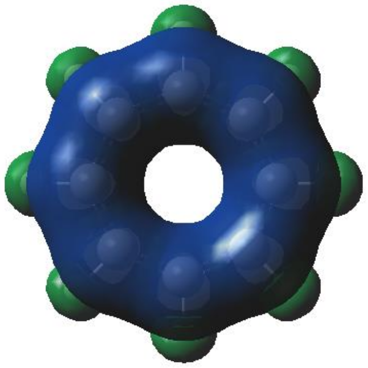

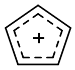

20

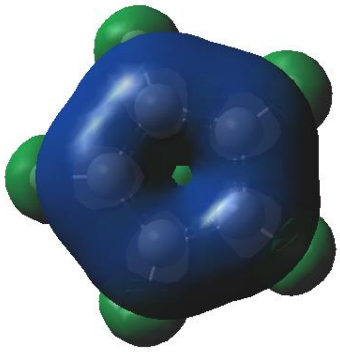

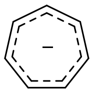

21

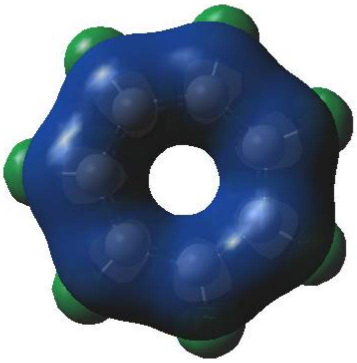

11 Method selection

During preliminary geometry optimizations, we noted that some methods gave homoaromatic minima for **1a** and **2a** while some did not. For these bicyclic systems, the homoaromatic minima are very shallow and it is not clear if they are true minima or flat parts of the PES in between other stationary points. Based on the comparison with higher-level methods we chose B2PLYP for our geometry optimizations. Further details are given below.

11.1 Method evaluation for compound 2

We performed an intrinsic reaction coordinate (IRC) calculation for the interconversion of homoaromatic **2a** and biradical **2b** with the τHCTH functional.[5] Then we continued with single-point calculations at the obtained geometries with ab initio wavefunction methods.

11.1.1 Comparison between DFT and ab initio methods

B2PLYP, B3LYP and OLYP are compared to CCSD(T) and CASPT2 in Figure S11. Both CASPT2 and CCSD(T) predict a minimum at the T_1 homoaromatic geometry, while OLYP and B3LYP give very shallow minima. Increasing the basis set from VDZP to VTZP for CASPT2 gives a shallower minimum, but the qualitative shape of the barrier remains the same. CASPT2 gives a lower barrier than CCSD(T). B2PLYP closely matches the shape of the CCSD(T) barrier and can be considered to give the best results both in energy and in barrier shape compared to CASPT2 and CCSD(T).

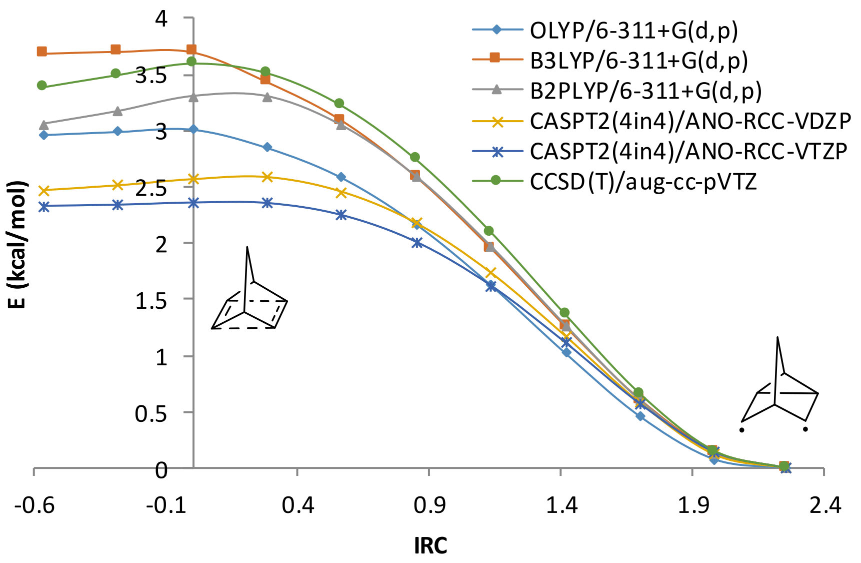

Figure S11. General method comparison for the IRC connecting **2a** and **2b** on the T_1 surface. The IRC was generated at the τHCTH/6-311+G(d,p) level.

11.1.2 Influence of multiconfigurational character

As indicated in Figure S12, the weight of the primary CASSCF configuration decreases when going from the biradical minimum **2b** to the homoaromatic minimum **2a**. The T_1 value is below 0.02, but is still quite high, indicating potential multireference character.

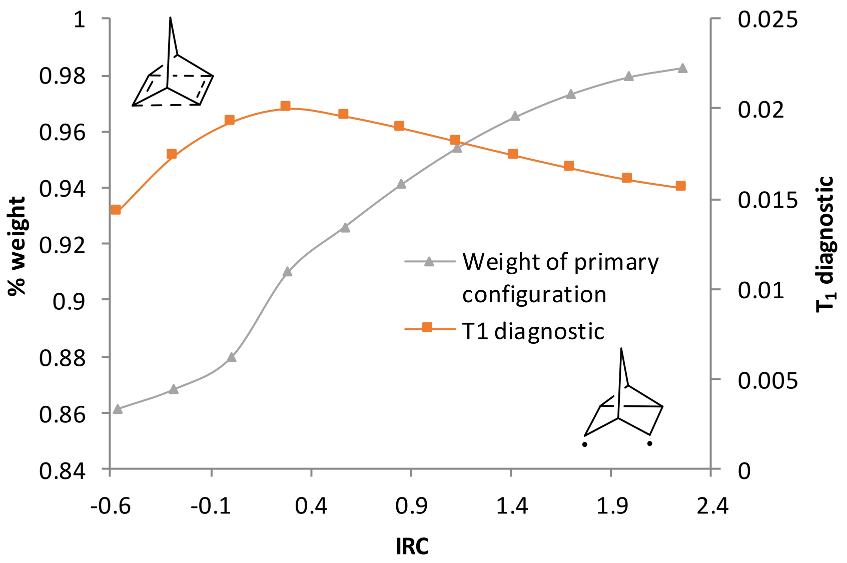

Figure S12. Weight of primary configuration in the CASSCF/ANO-RCC-VDZP calculations (left axis) and T_1 diagnostic for the CCSD/aug-cc-pVTZ wavefunction (right axis) as a function of the IRC between **2a** and **2b** on the T_1 surface.

BD(T), which is a method appropriate for treating multiconfigurational systems,[6] gives a flatter minimum than CCSD(T) (Figure S13). However, the shape of the PES is approximately the same and any multiconfigurational effects should be minor. Figure 4 also shows the basis set dependence of CCSD(T) and BD(T). 6-311+G(d,p) is clearly too small, while aug-cc-pVDZ gives results that are close to aug-cc-pVTZ. For **1**, we therefore used the aug-cc-pVDZ basis set for comparing energies (*vide infra*).

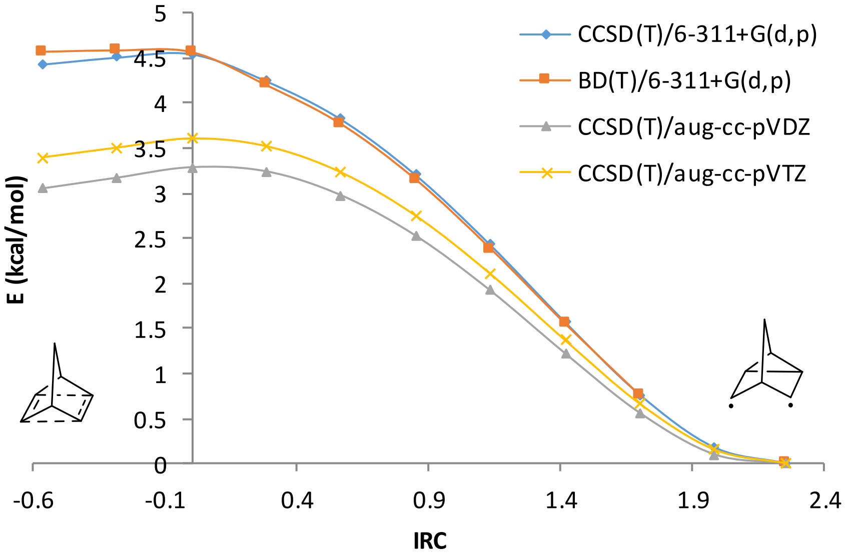

Figure S13. Comparison of CCSD(T) and BD(T) with different basis sets on the IRC connecting **2a** and **2b** on the T_1 surface.

11.1.3 Effect of basis set for CASPT2

The effect of changing the active space in CASPT2 is shown in Figure S14. Going from only including the π orbitals (4in4) to also including the σ part of the double bonds (8in8) or the four σ bonds from the sp^2 carbons to the bridgehead carbons (12in12) produce slight differences in the barrier heights but similar shapes. All active spaces indicate that the homoaromatic structure is a shallow minimum. Therefore, we conclude that it is appropriate to include only the π orbitals could be included in the active space. We here use the ANO-RCC-VDPZ basis set for comparison to save computational time.

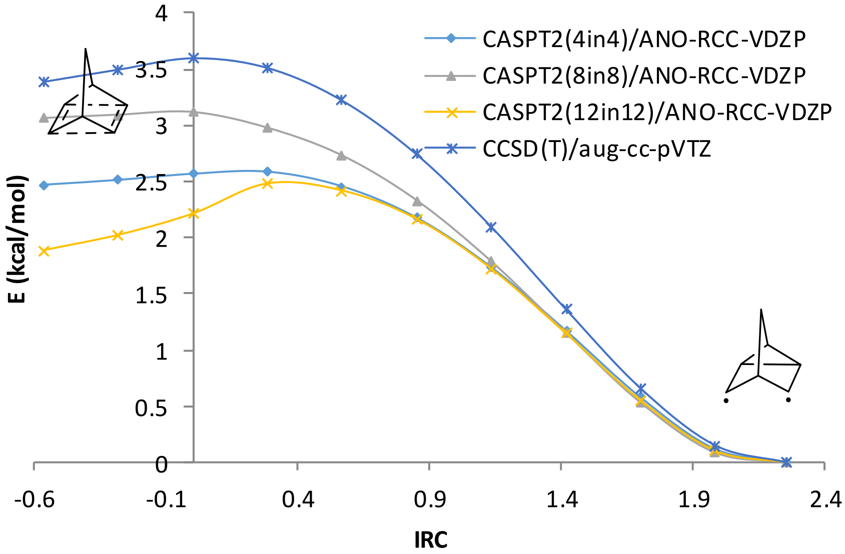

Figure S14. Comparison of CASPT2 with different active space on the IRC connecting **2a** and **2b** on the T_1 surface.

11.2 Method evaluation for compound 1

11.2.1 Comparison between DFT and ab initio methods

For **1**, the results of the single-point calculations along the τHCTH IRC are shown in Figure S15. The large discrepancy in barrier shape and energy difference between the two structures for B2PLYP as compared to the other methods is likely due to spin contamination (2.13 in **2b** vs. 2.02 in **2a**). Despite the spin contamination, the structure that B2PLYP gives for the diradical minimum is very close to those of B3LYP and OLYP.

The central question is if the homoaromatic structure is a minimum (which is predicted by B2PLYP and OLYP) or a maximum (as predicted by B3LYP) (Figure S16). CCSD(T) and CASPT2/ANO-RCC-VDZP gives a minimum, while CASPT2/ANO-RCC-VTZP does not.

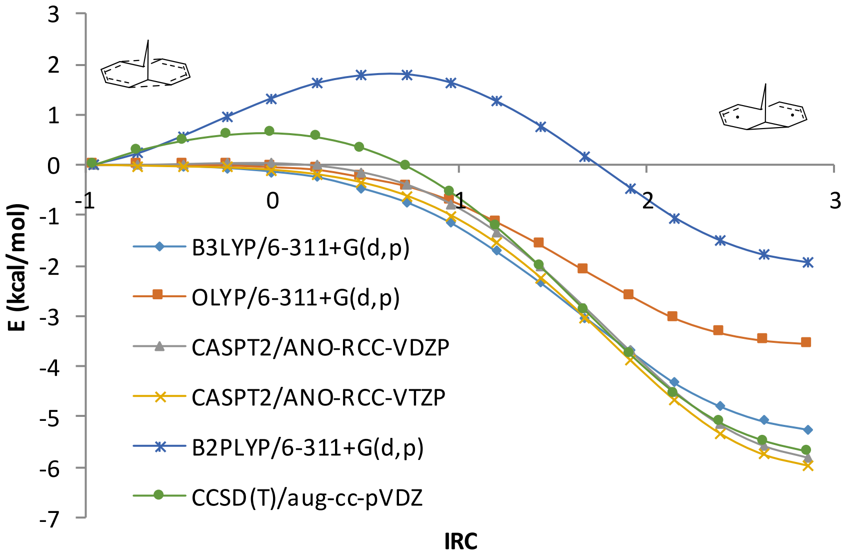

Figure S15. General method comparison for the IRC connecting **1a** and **1b** on the T_1 surface. The IRC was generated at the τHCTH/6-311+G(d,p) level.

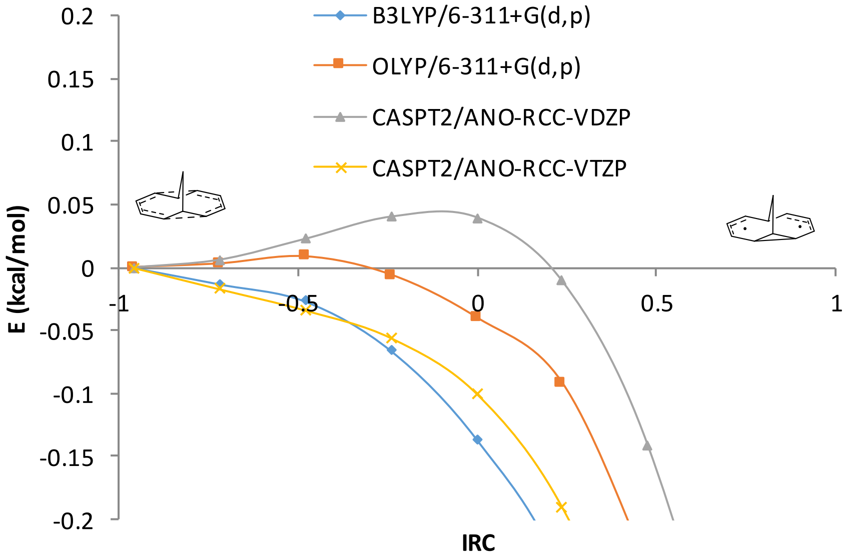

Figure S16. Close-up of general method comparison for the IRC connecting **1a** and **1b** on the T_1 surface. The IRC was generated at the τHCTH/6-311+G(d,p) level.

11.2.2 Influence of multiconfigurational character

The T_1 diagnostic is high at 0.027-0.031 along the IRC, indicating possible multireference character (Figure S17). This suspicion is further reinforced by the weight of the dominant configuration in CASSCF, which goes from 0.73 at the homoaromatic structure to 0.85 for the biradical structure.

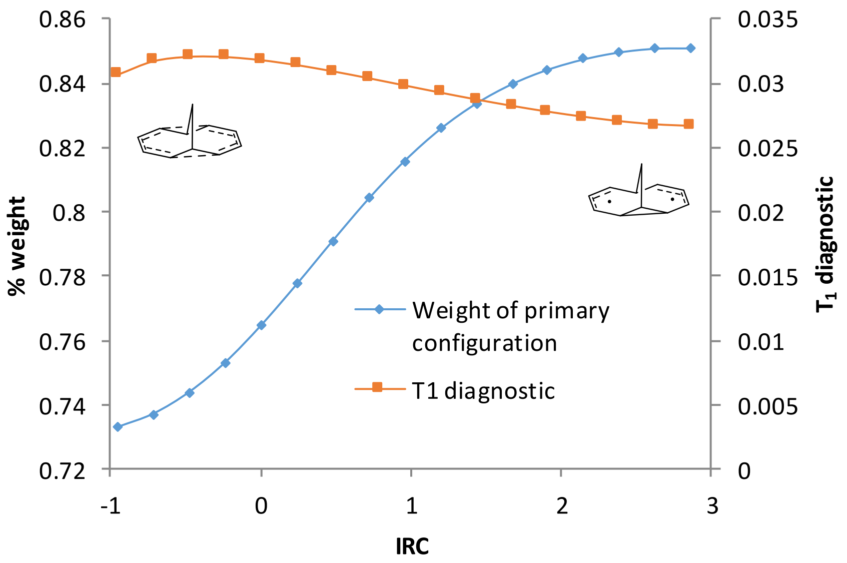

Figure S17. Weight of primary configuration in the CASSCF/ANO-RCC-VDZP calculations (left axis) and T_1 diagnostic for the CCSD/aug-cc-pVDZ wavefunction (right axis) as a function of the IRC between **2a** and **2b** on the T_1 surface.

To test further for multiconfigurational character, we compared CCSD(T) and BD(T). The main difference between BD(T) and CCSD(T) is a lowering of the barrier with BD(T) (Figure S17). As the difference in the profiles is so small, we conclude that usage of multiconfigurational methods is not needed. However, it is not clear whether there is a true minimum or not as CASPT2 and BD(T)/CCSD(T) calculations give different results.

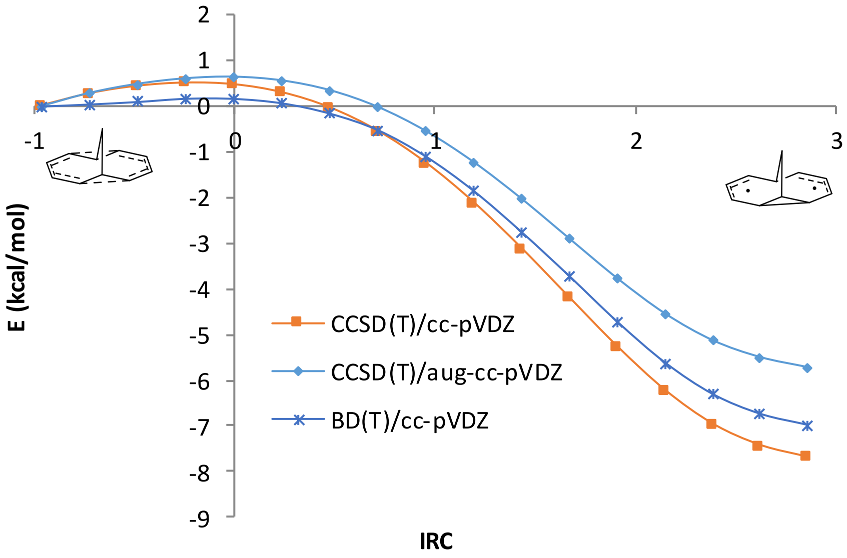

Figure S18. Comparison of CCSD(T) and BD(T) with different basis sets on the IRC connecting two minima on the T_1 surface of **1**.

11.3 Choice of functional

Based on the results above, we believe that the B2PLYP functional is preferred over OLYP and B3LYP for geometry optimization and evaluation of properties. The T_1-homoaromatic minima for the bicyclic systems **1** and **2** are shallow and it will depend on the method if they are really true minima or merely flat parts of the PES.

12 Energy differences with selected methods

The energies of T_1 and S_0 structures relative to the most stable S_0 structure are given in Table S3 for compounds **1-11**. The results for B2PLYP/6-311+G(d,p) are compared to B3LYP/6-311+G(d,p) and OLYP/6-311+G(d,p) optimized separately, as well as the high-level CASPT2/ANO-RCC-VTZP and CCSD(T)/aug-cc-pVTZ (for the small molecules) at the B2PLYP geometries. For CASPT2, all orbitals of π symmetry were included (or those corresponding to π orbitals in the homoaromatic isomer). Generally, B2PLYP gives relative energies which are closer to the ones given by CCSD(T) and CASPT2, further supporting its choice as the main method in this project. Additionally, for all structures, the OLYP/6-311+G(d,p) and B3LYP/6-311+G(d,p) geometries are very similar to B2PLYP/6-311+G(d,p).

Table S3: Energies relative to the most stable S_0 isomer. Results at the B3LYP/6-311+G(d,p), OLYP/6-311+G(d,p), B2PLYP/6-311+G(d,p), CASPT/ANO-RCC-VTZP//B2PLYP/6-311+G(d,p) and CCSD(T)-aug-cc-pVTZ//B2PLYP/6-311+G(d,p) levels.

	State	B3LYP	OLYP	B2PLYP	CASPT2	CCSD(T)
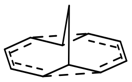 **1a**	T_1	47.1	42.0	47.3	51.5	-
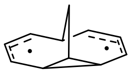 **1b**	T_1	41.8	38.4	45.4	45.6	-
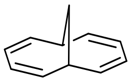 **1c**	S_0	0	0	0	0	-
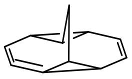 **1d**	S_0	20.8	14.8	18.8	14.1	-
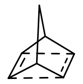 **2a**	T_1	58.9	55.1	60.0	61.5	62.1
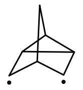 **2b**	T_1	55.2	52.1	56.9	59.2	58.7
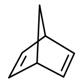 **2c**	S_0	0	0	0	0	0

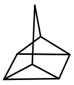 **2d**	S_0	23.4	14.7	24.0	21.0	23.7
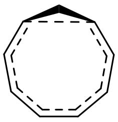 **3a**	T_1	25.6	24.0	30.5	33.8	-
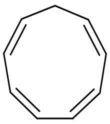 **3b**	S_0	0	0	0	0	-
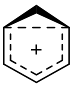 **4a**	T_1	50.4	50.6	52.1	54.0	52.9
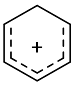 **4b**	S_0	0	0	0	0	0
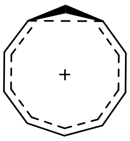 **5a**	T_1	18.0	17.1	20.3	22.2	-
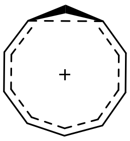 **5b**	T_1	27.8	24.7	28.1	28.6	-
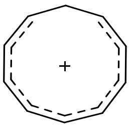 **5c**	S_0	0	0	0	0	-
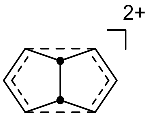 **6a**	T_1	49.1	46.5	49.2	47.1	-
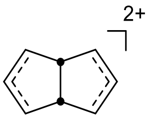 **6b**	S_0	0	0	0	0	-
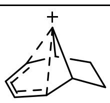 **7a**	T_1	38.2	41.5	43.1	*	-

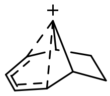 **7b**	S_0	0	0	0	*	-
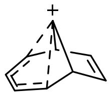 **8a**	T_1	18.1	21.9	22.3	28.0	-
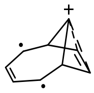 **8b**	T_1	48.2	47.5	50.5	52.4	-
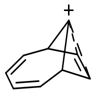 **8c**	S_0	0	0	0	0	-
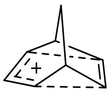 **9a**	T_1	27.8	26.7	29.7	28.8	31.2
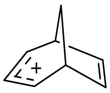 **9b**	S_0	0	0	0	0	0
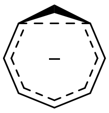 **10a**	T_1	30.6	31.4	31.2	31.9	-
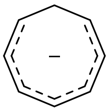 **10b**	S_0	0	0	0	0	-
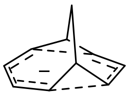 **11a**	T_1	16.8	16.9	20.1	21.2	-
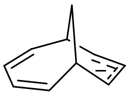 **11b**	S_0	0	0	0	0	-

* **7a** and **7b** were not calculated with CASSCF as it is difficult to choose a consistent active space (**7b** is a non-classical carbocation).

13 Analysis of triplet wavefunctions

A detailed analysis of the wavefunctions were conducted to determine if DFT is appropriate to study these systems (Table S4). We calculated T_1 diagnostic values for selected compounds to detect potential multireference character.[7] Although the values for **4** and **9** warrant caution,[8] CASSCF calculations reveal that the triplet states are dominated by a single configuration. $\langle S^2 \rangle$ values are generally low both for B2PLYP and B3LYP. The B2PLYP values for **3** (2.11) and **11** (2.09) are somewhat high, but as shown by Radom,[9] doubly hybrid DFT is insensitive to moderate amounts of spin contamination and much more resilient than HF or MP2. In summary, we are confident that DFT is suitable to study these compounds.

Table S4: $\langle S^2 \rangle$ values at the B2PLYP/6-311+G(d,p) and B3LYP/6-311+G(d,p) levels. T_1 diagnostics at the CCSD(T)/aug-cc-pVTZ level. CASSCF weights with the ANO-RCC-VTZP basis set

	**$\langle S^2 \rangle$ value**		**T_1 diag.**	**CASSCF weight**	
	**B3LYP**	**B2PLYP**		**Primary**	**Second highest**
**1**	2.01	2.02	-	0.74	0.08
**2**	2.01	2.02	0.013	0.86	0.09
**3**	2.03	2.11	-	0.82	0.01
**4**	2.01	2.01	0.024	0.94	0.01
**5**	2.01	2.02	-	0.83	0.01
**6**	2.01	2.01	-	0.90	0.03
**7**	2.01	2.04	-	0.95	0.02
**8**	2.01	2.05	-	0.91	0.03
**9**	2.01	2.02	0.030	0.93	0.03
**10**	2.01	2.05	-	0.90	0.01
**11**	2.02	2.09	-	0.89	0.03

14 Effect of dispersion-corrected DFT

To test for the effect of dispersion[10] on the DFT results, we optimized one neutral, one cationic and one anionic compound with B2PLYP-D3(BJ) and compared to the B2PLYP geometries (Figure S19). The differences are very small and therefore we conclude that the findings of this study would not be changed by inclusion of dispersion corrections in the calculations.

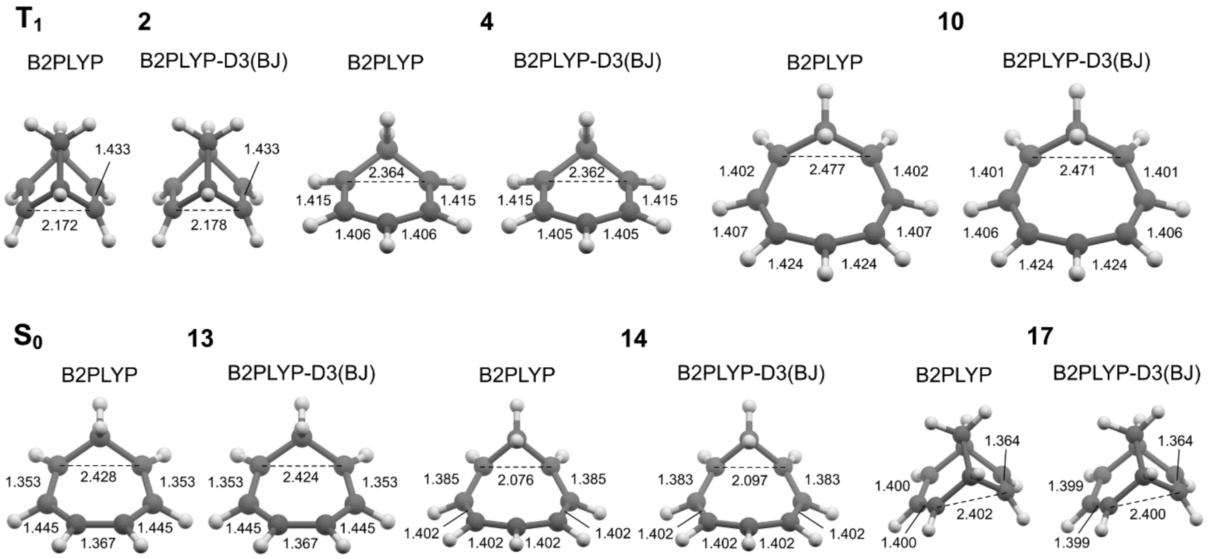

Figure S19. Comparison of B2PLYP and B2PLYP-D3(BJ) geometries for selected compounds. Basis set: 6-311+G(d,p).

15 ICSS plots

To investigate more closely the three-dimensional behaviour of the magnetic properties, we calculated iso-chemical- shielding surfaces (ICSSs) that clearly visualize regions of shielding/de-shielding.[11] We selected **3**, **5**, **8**, **13**, **14** and **18** as representative neutral, cationic and anionic homoaromatic compounds in S_0 and T_1. They display the out-of-plane shielding and in-plane de-shielding regions typical for aromatic compounds. Moreover, the shielding clouds above and below the ring are extensive, indicating that the results from one-dimensional NICS scans should not be overly sensitive to the exact direction or origin. This is in line with the results for **5b** above where two directions were tried.

	**3**	**5**	**8**
T_1	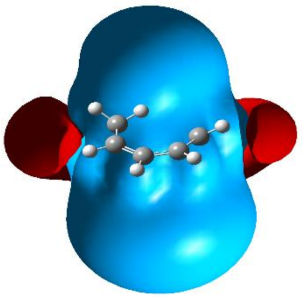	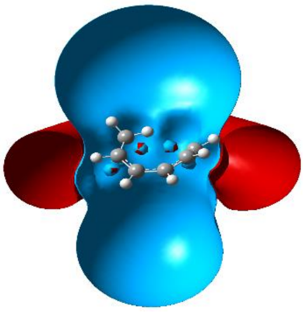	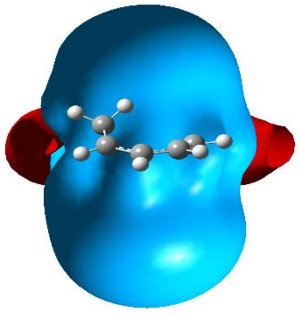
	**13**	**14**	**18**
S_0	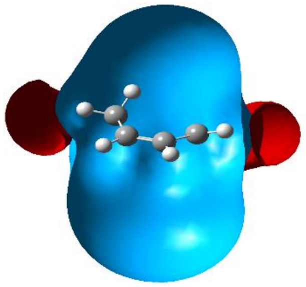	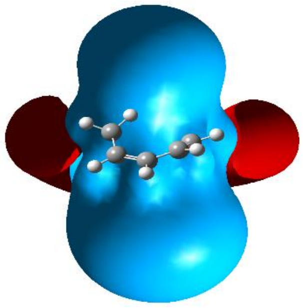	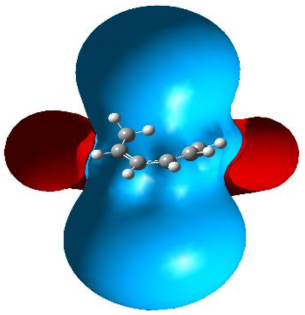

Figure S20. ICSS plots at the GIAO-B3LYP/6-311+G(d,p) level with a isosurface value of 0.2 ppm. Blue regions are shielding and red regions deshielding.

16 Atomic coordinates and eneriges

Cartesian coordinates are given at the B2PLYP/6-311+G(d,p) level of theory in a separate file. Coordinates for the bq atoms used in the NICS scans are also given where applicable.

17 References

Full Gaussian references

Gaussian 09, Revision D.01 and E.01, M. J. Frisch, G. W. Trucks, H. B. Schlegel, G. E. Scuseria, M. A. Robb, J. R. Cheeseman, G. Scalmani, V. Barone, B. Mennucci, G. A. Petersson, H. Nakatsuji, M. Caricato, X. Li, H. P. Hratchian, A. F. Izmaylov, J. Bloino, G. Zheng, J. L. Sonnenberg, M. Hada, M. Ehara, K. Toyota, R. Fukuda, J. Hasegawa, M. Ishida, T. Nakajima, Y. Honda, O. Kitao, H. Nakai, T. Vreven, J. A. Montgomery, Jr., J. E. Peralta, F. Ogliaro, M. Bearpark, J. J. Heyd, E. Brothers, K. N. Kudin, V. N. Staroverov, R. Kobayashi, J. Normand, K. Raghavachari, A. Rendell, J. C. Burant, S. S. Iyengar, J. Tomasi, M. Cossi, N. Rega, J. M. Millam, M. Klene, J. E. Knox, J. B. Cross, V. Bakken, C. Adamo, J. Jaramillo, R. Gomperts, R. E. Stratmann, O. Yazyev, A. J. Austin, R. Cammi, C. Pomelli, J. W. Ochterski, R. L. Martin, K. Morokuma, V. G. Zakrzewski, G. A. Voth, P. Salvador, J. J. Dannenberg, S. Dapprich, A. D. Daniels, Ö. Farkas, J. B. Foresman, J. V. Ortiz, J. Cioslowski, and D. J. Fox, Gaussian, Inc., Wallingford CT, 2009.

Gaussian 16, Revision A.03, M. J. Frisch, G. W. Trucks, H. B. Schlegel, G. E. Scuseria, M. A. Robb, J. R. Cheeseman, G. Scalmani, V. Barone, G. A. Petersson, H. Nakatsuji, X. Li, M. Caricato, A. V. Marenich, J. Bloino, B. G. Janesko, R. Gomperts, B. Mennucci, H. P. Hratchian, J. V. Ortiz, A. F. Izmaylov, J. L. Sonnenberg, D. Williams-Young, F. Ding, F. Lipparini, F. Egidi, J. Goings, B. Peng, A. Petrone, T. Henderson, D. Ranasinghe, V. G. Zakrzewski, J. Gao, N. Rega, G. Zheng, W. Liang, M. Hada, M. Ehara, K. Toyota, R. Fukuda, J. Hasegawa, M. Ishida, T. Nakajima, Y. Honda, O. Kitao, H. Nakai, T. Vreven, K. Throssell, J. A. Montgomery, Jr., J. E. Peralta, F. Ogliaro, M. J. Bearpark, J. J. Heyd, E. N. Brothers, K. N. Kudin, V. N. Staroverov, T. A. Keith, R. Kobayashi, J. Normand, K. Raghavachari, A. P. Rendell, J. C. Burant, S. S. Iyengar, J. Tomasi, M. Cossi, J. M. Millam, M. Klene, C. Adamo, R. Cammi, J. W. Ochterski, R. L. Martin, K. Morokuma, O. Farkas, J. B. Foresman, and D. J. Fox, Gaussian, Inc., Wallingford CT, 2016.

[1] M. Mandado, A. M. Graña and I. Pérez-Juste, *J. Chem. Phys.*, 2008, **129**, 164114.
[2] CYLview, 1.0b; Legault, C. Y., Université de Sherbrooke, 2009 (http://www.cylview.org)
[3] A. Stanger, *J. Org. Chem.*, 2006, **71**, 883–893.
[4] GaussView, Version 5, R. Dennington, T. Keith and J. Millam, *Semichem Inc*., Shawnee Mission KS, 2009.
[5] A. D. Boese and N. C. Handy, *J. Chem. Phys.*, 2002, **116**, 9559–9569.
[6] C. J. Cramer, *Essentials of computational chemistry: theories and models*, Wiley, Chichester, U. K., 2nd edn, 2004.
[7] T. J. Lee and P. R. Taylor, *Int. J. Quantum Chem.*, 1989, **36**, 199–207.
[8] C. J. Cramer, *Essentials of computational chemistry: theories and models*, Wiley, Chichester, U. K., 2nd edn, 2004.
[9] A. S. Menon and L. Radom, *J. Phys. Chem. A*, 2008, **112**, 13225–13230.
[10] S. Grimme, S. Ehrlich and L. Goerigk, *J. Comput. Chem.*, 2011, **32**, 1456–1465.
[11] S. Klod and E. Kleinpeter, *J. Chem. Soc. Perkin Trans. 2*, 2001, 1893–1898.
